# Evolution of Self-Organized Task Specialization in Robot Swarms

**DOI:** 10.1371/journal.pcbi.1004273

**Published:** 2015-08-06

**Authors:** Eliseo Ferrante, Ali Emre Turgut, Edgar Duéñez-Guzmán, Marco Dorigo, Tom Wenseleers

**Affiliations:** 1 Laboratory of Socio-Ecology and Social Evolution, Zoological Institute, KU Leuven, Leuven, Belgium; 2 Mechanical Engineering Department, Middle East Technical University, Ankara, Turkey; 3 IRIDIA–CoDE, Université Libre de Bruxelles, Brussels, Belgium; Indiana University, UNITED STATES

## Abstract

Division of labor is ubiquitous in biological systems, as evidenced by various forms of complex task specialization observed in both animal societies and multicellular organisms. Although clearly adaptive, the way in which division of labor first evolved remains enigmatic, as it requires the simultaneous co-occurrence of several complex traits to achieve the required degree of coordination. Recently, evolutionary swarm robotics has emerged as an excellent test bed to study the evolution of coordinated group-level behavior. Here we use this framework for the first time to study the evolutionary origin of behavioral task specialization among groups of identical robots. The scenario we study involves an advanced form of division of labor, common in insect societies and known as “task partitioning”, whereby two sets of tasks have to be carried out in sequence by different individuals. Our results show that task partitioning is favored whenever the environment has features that, when exploited, reduce switching costs and increase the net efficiency of the group, and that an optimal mix of task specialists is achieved most readily when the behavioral repertoires aimed at carrying out the different subtasks are available as pre-adapted building blocks. Nevertheless, we also show for the first time that self-organized task specialization could be evolved entirely from scratch, starting only from basic, low-level behavioral primitives, using a nature-inspired evolutionary method known as Grammatical Evolution. Remarkably, division of labor was achieved merely by selecting on overall group performance, and without providing any prior information on how the global object retrieval task was best divided into smaller subtasks. We discuss the potential of our method for engineering adaptively behaving robot swarms and interpret our results in relation to the likely path that nature took to evolve complex sociality and task specialization.

## Author Summary

Many biological systems execute tasks by dividing them into finer sub-tasks first. This is seen for example in the advanced division of labor of social insects like ants, bees or termites. One of the unsolved mysteries in biology is how a blind process of Darwinian selection could have led to such highly complex forms of sociality. To answer this question, we used simulated teams of robots and artificially evolved them to achieve maximum performance in a foraging task. We find that, as in social insects, this favored controllers that caused the robots to display a self-organized division of labor in which the different robots automatically specialized into carrying out different subtasks in the group. Remarkably, such a division of labor could be achieved even if the robots were not told beforehand how the global task of retrieving items back to their base could best be divided into smaller subtasks. This is the first time that a self-organized division of labor mechanism could be evolved entirely de-novo. In addition, these findings shed significant new light on the question of how natural systems managed to evolve complex sociality and division of labor.

## Introduction

The “major transitions in evolution”, whereby cells teamed up to form multicellular organisms or some animals went on to live in societies, are among the keys to the ecological success of much life on earth [1]. The efficiency of both organisms and animal societies frequently depends on the presence of an advanced division of labor among their constituent units [2–4]. The most celebrated examples can be found in social insects, which exhibit astonishing levels of social organization and are ecologically dominant in many natural ecosystems [5,6]. Through division of labor, social insects can perform complex tasks by dividing them up into smaller sub-tasks carried out by different sets of individuals [7–10]. Although the adaptive benefits of division of labor are evident, the way in which it can evolve is more enigmatic, since an effective division of labor requires the simultaneous co-occurrence of several complex traits, including self-organized mechanisms to decompose complex tasks into simpler subtasks, mechanisms to coordinate the execution of these tasks, mechanisms to allocate an appropriate number of individuals to each task, and the ability of individuals to effectively carry out each of the subtasks [4]. The complexity of this co-evolutionary problem is further exacerbated by the fact that division of labor should also be flexible to be able to cope with changing environmental conditions [4,10,11].

To date, most analytical and individual-based simulation models of division of labor [4,9,10,12–16] have focused merely on determining the optimal proportion of individuals engaging in different tasks [12] or on determining optimal task allocation mechanisms [4,9,10,13,16], sometimes in relation to particular levels of intragroup genetic variation [14,15]. These studies implicitly assume that pre-optimized behaviors to carry out each of the different subtasks, which we refer to as “pre-adapted behavioral building blocks”, are already present in nonsocial ancestors [17], and that division of labor merely involves the rewiring of these behaviors. Empirical support for this hypothesis can be found for example in the somatic cell differentiation in multicellular organisms, which is derived from a genetic switch involved in the induction of diapause during stress periods in unicellular ancestors [2,18]. Similarly, in insect societies, worker brood care is thought to be derived from ancestral parental care [19], and reproductive division of labor as well as worker task specialization may be derived from mechanisms that initially regulated reproduction and foraging in solitary ancestors [17,20–22].

A limitation of traditional analytical modeling approaches to division of labor [4,10], however, is that they can only consider a finite and pre-specified number of behavioral strategies. In recent years, artificial evolution of teams of embodied agents has been used to enable the study of social traits in more detail, taking into account more realistic physical constraints and a much larger set of allowable behaviors and strategies [23–25]. In evolutionary swarm robotics, for example, this framework has been used to study the evolution of the origin of communication [26,27], collective transport [28], collective motion [29], aggregation [30–32] and chain formation [33] (reviewed in [23,24,34–37]). Nevertheless, to date, no study in evolutionary swarm robotics has succeeded in evolving complex, self-organized division of labor entirely de novo [38,39]. This may be due to the fact that most evolutionary robotics studies have made use of neural network-based approaches [23–25,36], which have been shown to scale badly to more complex problems [38,40].

The main aim of our study was to test if other nature-inspired evolutionary methods than traditionally used in evolutionary swarm robotics would be able to achieve complex task specialization in social groups. Analogously to the situation in nature where subtask behaviors may or may not be recycled from pre-adapted behavioral building blocks, we do this using one of two approaches, in which we either do or do not pre-specify the behaviors required for carrying out the different subtasks. Evidently, we expected that task specialization could evolve much more easily when pre-adapted behavioral building blocks were present, but we were also interested to see if a self-organized mechanism of task specialization could be evolved entirely de-novo using our recently developed method of Grammatical Evolution [41]. This nature-inspired evolutionary method allows a set of low-level behavioral primitives to be recombined and evolved into complex, adaptive behavioral strategies through the use of a generative encoding scheme that is coupled with an evolutionary process of mutation, crossover and selection [41].

**Fig 1:**
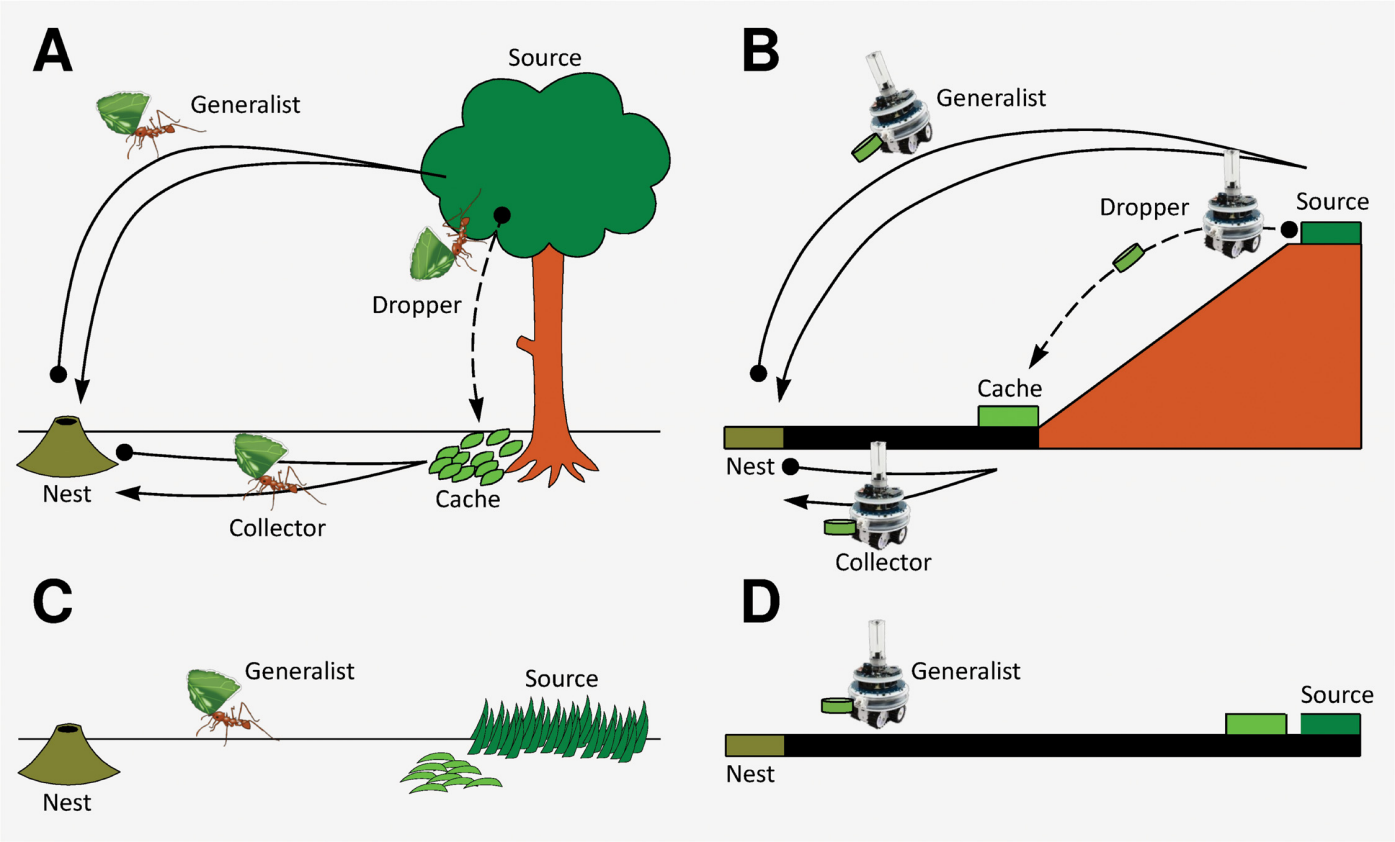
Task partitioning in insects and robots. (a) Task partitioned retrieval of leaf fragments, as found in most *Atta* leafcutter ants that harvest leaves from trees [7,43]. Dropper ants cut leaves which then accumulate in a cache, after which the leaves are retrieved by collectors and brought back to the nest, where they serve as a substrate for a fungus which is farmed as food. Ants also occasionally use a generalist strategy whereby both tasks are performed by the same individuals. (b) Analogous robotics setup, whereby items have to be transported across a slope using the coordinated action of droppers, collectors and possibly generalists. (c) Grass cutting leafcutter ants cutting leaf fragments in a flat environment without task partitioning, using a generalist foraging strategy [49]. (d) Analogous robotics setup, with robots being required to collect items in a flat arena.

**Fig 2:**
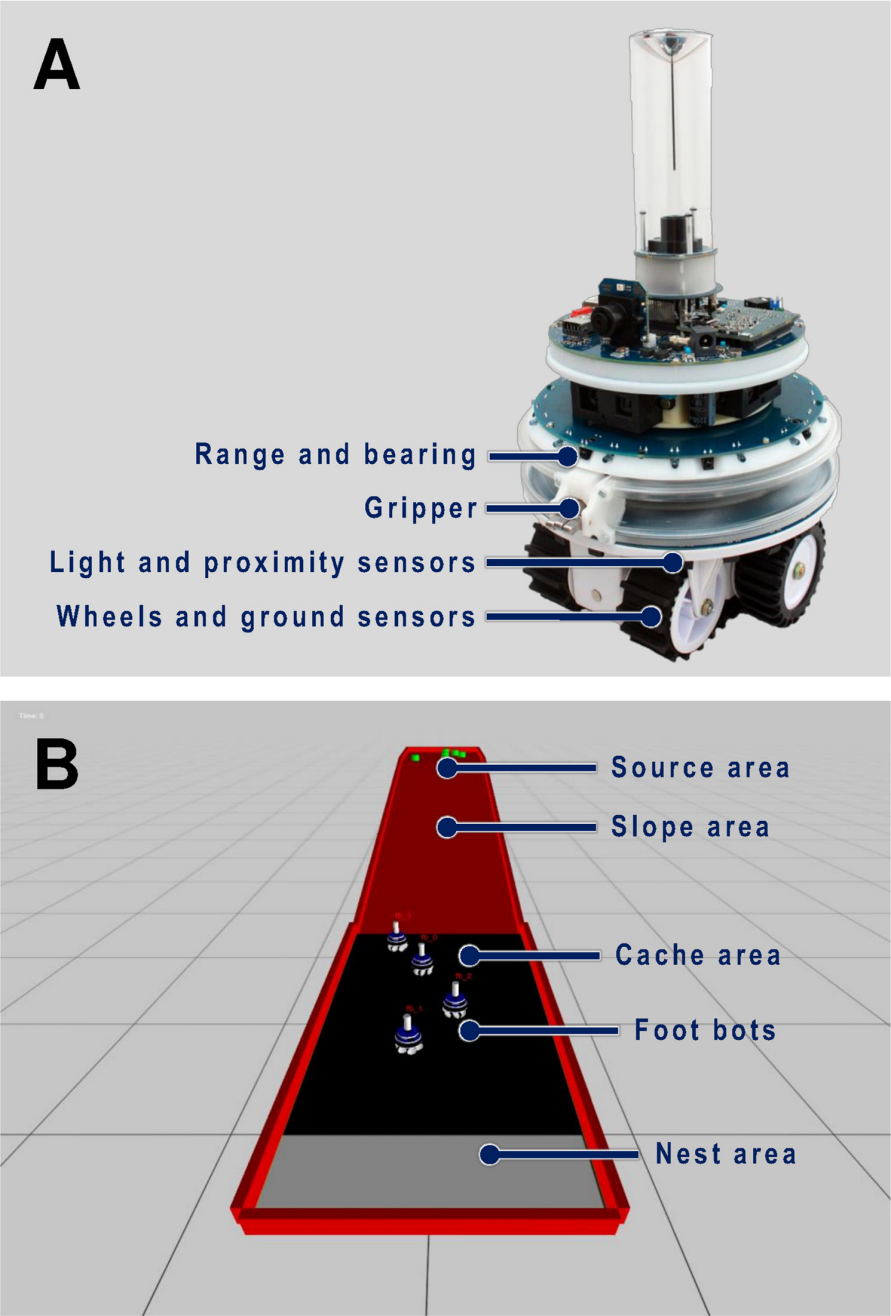
Foot-bot robots and ARGoS simulation platform. (a) The foot-bot robot [44] and its sensors and actuators. (b) A snapshot of the ARGoS [45], the physics-based simulator used in our experiments. The snapshot shows the different elements composing our experimental setup. having a width of 1.75 m and a length of 9.75 m. The inclination of the slope is 8 degrees.

The type of division of labor we consider in our set-up is known as “task partitioning”, and requires different tasks to be carried out in sequence by different sets of individuals [7]. In particular, our experimental scenario was inspired by a spectacular form of task partitioning found in some leafcutter ants, whereby some ants (“droppers”) cut and drop leaf fragments into a temporary leaf storage cache and others (“collectors”) specialize in collecting and retrieving the fragments back to the nest [42,43] (Fig 1). In our analogous robotics setup, we used a team of robots [44] simulated in-silico using an embodied swarm robotics simulator [45] (Fig 2) and required the robots to collect items and bring them back to the nest in either a flat or sloped environment (see Fig 1 and Fig 2B and Material and Methods). In this setup, task specialization should be favored whenever some features of the environment (in our case, the presence of a slope) can be exploited by the robots to achieve faster foraging (“economic transport”, [46]) and reduce switching costs between different locations [9,47]. The results of these experiments show for the first time that complex, self-organized task specialization and task allocation could be evolved in teams of robots. Nevertheless, a fitness landscape analysis also demonstrates that task specialization was much easier to evolve when pre-evolved behavioral building blocks were present. We use these findings as a starting point to speculate about the likely path that nature took to evolve complex sociality and division of labor. Furthermore, we discuss the potential of our nature-inspired evolutionary method for the automated design of swarms of robots displaying complex forms of coordinated, social behavior.

## Materials and Methods

### The task and the environment

Our experimental setup is inspired by the type of task partitioning observed in *Atta* leafcutter ants [42,43], that collect leaves and other plant material as a substrate for a fungus that is farmed as food (Fig 1A). In these insects, particularly in species that harvest leaves from trees, leaf fragments are retrieved in a task partitioned way, whereby some ants (“droppers”) specialize in cutting and dropping leaf fragments to the ground, thereby forming a leaf cache, and others specialize in collecting leaves from the cache to bring them back to the nest (“collectors”) [42,43]. In addition, another strategy is known whereby the whole leaf cutting and retrieval task is carried out by single individuals (“generalists”), without any task partitioning [42,43]. Task partitioning in this scenario is thought to be favored particularly in situations where the ants forage on leaves from trees, due to the fact that the leaf fragments can then be transported purely by gravity, which saves the ants the time to climb up and down the tree, and the fact that there are few or no costs associated with material loss thanks to the large supply of leaves [7,43,48] (Fig 1A). This theory is supported by the fact that species living in more homogeneous grassland usually retrieve leaf fragments in an unpartitioned way, without first dropping the leaves (Fig 1C), particularly at close range to the nest [43,49].

In the corresponding robotic setup, we substituted the tree with a *slope area* and leaves with cylindrical items. A team of robots then had to collect these items from what we call the *source area* and bring them back to what we refer to as the *nest area* (Fig 1B). Simulations were carried out using the realistic, physics-based simulator ARGoS [45]. As demonstrated in the past, controllers developed within ARGoS can be directly transferred to real robots with minimal or no intervention [50,51]. The robots involved in the experiments were a simulated version of the foot-bot robot, a version of the MarXbot robot [44], which is a differential-drive, non-holonomic, mobile robot (Fig 2A). A screen-shot of a simulation instant is shown in Fig 2B. We used a setup whereby 5 items were always present in the source area. The 5 items were replaced and put in a random position within the source area each time a robot picked up one of them. This is justified by the fact that leaf availability in the natural environment is often virtually unlimited. A light source was placed at a height of 500 m, 500 m away from the nest, in the direction of the source area. The light allowed the robots to navigate in the environment, since phototaxis allowed them to go towards the item source, whereas anti-phototaxis allowed them to return to the nest. The slope area had an inclination of about 8 degrees. The linear velocity of the robots on the flat part of the arena was 0.15 m/s, but this reduced to a maximum speed of 0.015 m/s when they had to climb up the slope, and increased to 0.23 m/s when they came down from the slope. If an item was dropped in the slope area, it slid down the slope at a speed of 1 m/s until it reached the *cache area*, where it was stopped due to friction and to the impact with other items in the cache. This was done to simulate leaves being dropped from the tree, as in Fig 1A. In addition, in some of the experiments, we considered a flat environment of the same length and width as the one described above (Fig 1D), to mirror the case in nature where ants forage in a flat, homogeneous environment (Fig 1C).

### Evolution of task-partitioning from pre-adapted building blocks

In a first set of experiments, we assumed that the behavioral strategies required to carry out each of the subtasks (dropper or collector behavior, as well as generalist, solitary foraging) were available to the robots as pre-adapted behavioral building blocks and then determined the optimal mix of each of the strategies [12]. This setup, therefore, matched some evolutionary scenarios proposed for the origin of division of labor in biological systems based on co-opting pre-adapted behavioral patterns [2,17–22]. In addition, this scenario allowed us to determine under which environmental conditions task partitioning is favored, and provided a fitness benchmark for the second scenario below, where task partitioning was evolved entirely de-novo.

In this first set of experiments, dropper, collector and generalist foraging strategies were implemented as follows:

1. ***Dropper strategy*:** A dropper robot is a robot that climbs the slope area and never descends it again, continuously collecting items from the source area and dropping them to the slope area.
2. ***Collector strategy*:** A collector robot is a robot that never climbs the slope area. Instead, it continuously collects items from the cache (when present) and brings them back to the nest. If it cannot find any items, the collector robot keeps exploring the cache area by performing random walk, until an item is found.
3. ***Generalist strategy*:** A generalist robot is a robot that performs a standard foraging task. It climbs the slope and explores the source area, collects items, and brings them all the way back to the nest. The generalist robot does not explore the cache area, but in case it finds an item at the cache while going towards the source, it collects it and brings it back to the nest.

The rules that we employed to implement these strategies are shown in S1 Table. We also assumed that the robots would specialize for life in each of these available strategies according to a particular evolved allocation ratio. This was equivalent to assuming that in nature, these behavioral strategies would already have evolved due to selection in their ancestral environment, and that natural selection would favor a particular hard-wired individual allocation between the different sets of tasks, e.g. through fine-tuning of the probability of expression of the gene-regulatory networks coding for the different behavioral patterns. For these experiments, we used teams of 4 robots, to match the evolutionary experiments with fine-grained building blocks (cf. next section). Subsequently, a fitness landscape analysis was used to determine the optimal mix between the three strategies in one of two possible environments, a flat or a sloped one (Fig 1B and 1D). This was done via exhaustive search, that is, by testing all possible ratio combinations and determining the corresponding fitness values in the two environments, rather than using an evolutionary algorithm. This was possible due to the relatively small search space, which gave access to the full fitness landscape. Group performance, measured by the total number of items retrieved to the nest over a period of 5,000 simulated seconds, for each possible mix of the three strategies, was measured in 10 simulated runs and then averaged.

### Evolution of task-partitioning from first principles

In a second set of experiments, we considered an alternative scenario where both task specialization and task allocation could evolve entirely de-novo, starting only from basic, low-level behavioral primitives. These primitives were simply navigational behaviors allowing robots to either go towards the source or towards the nest, as well as a random walk behavior:

1. ***PHOTOTAXIS*:** uses the light sensor to make the robot go towards the direction with the highest perceived light intensity.
2. **ANTI-PHOTOTAXIS**: uses the light sensor to make the robot go towards the lowest perceived light intensity.
3. **RANDOM WALK**: makes the robot move forward for a random amount of time and then turn to a random angle, repeating this process while the block is activated, without using any sensors.

In addition, a mechanism of obstacle avoidance, based on the robot’s range and bearing and proximity sensors, was switched on at all times to avoid that the robots would drive into each other or into the walls of the foraging arena. Finally, two instantaneous actions were allowed, namely picking up and dropping an item. To be able to evolve adequate behavioral switching mechanisms, we allowed the robots to perceive their position in space, that is, whether they were in the source, slope, cache or nest, based on sensorial input from the ground and light sensors, as well as perceive whether or not they were currently holding an item.

The fine-grained behavioral building blocks were combined together using a method known as grammatical evolution [52] as implemented in GESwarm [41], in order to evolve rule-based behaviors representing more complex strategies. GESwarm was developed for the automatic synthesis of individual behaviors consisting of rules leading to the desired collective behavior in swarm robotics. These rules were represented by strings, which in turn were generated by a formal grammar. The space of strings of such a formal grammar was used as a behavioral search space, and mutation, crossover and selection were then used to favor controllers that displayed high group performance.

The individual behavior of a given robot was expressed by a set R composed of an arbitrary number *n*_*R*_ of *rules R*_*i*_:

$$R=\left\{R_{i}\right\},i\in \{1,\ldots ,n_{R}\}.$$

Each rule was composed of three components:

$$R_{i}=P_{i}\times B_{i}\times A_{i},$$

where $B_{i}$ denotes a subset of all possible fine-grained behavioral building blocks (phototaxis, anti-phototaxis and random walk), $A_{i}$ denotes a subset of all possible instantaneous *actions* (pickup, drop, change behavior or change an internal state variable) and $P_{i}$ denotes a subset of all possible *preconditions*. The preconditions were specified as logical conditions with respect to the current value of a number of state variables, which included both sensorial input (the environment they were in and whether or not they were carrying an item) and internal state variables (a state variable that specified whether they wanted to pick up an item or not and two memory state variables, with evolvable meaning).

If all the preconditions in $P_{i}$ were met, and if a given robot was executing any of the low-level behaviors present in $B_{i}$, all actions contained in $A_{i}$ were executed with evolvable probability *p*_*l*_. In this way, we could allow the evolution of probabilistic behaviors, which have been extensively used both in the swarm robotics literature [53,54] and as microscopic models of the behavior of some social animals [55,56]. Finally, each robot executed all rules and actions in their order of occurrence.

To be able to generate the rules above, we devised a grammar using the Extended Backus-Naur Form notation [57]. Within the framework of grammatical evolution [41,52], a genotype represented a sequence of production rules to be followed to produce a valid string (in our case a set of rules) starting from that grammar. Mutation and crossover acted at the level of this genotype, modifying the sequence of production rules. The full grammar of GESwarm is described in [41].

Biologically speaking, our GESwarm rule-based controllers can be considered analogous to gene-regulatory networks or to logic circuits in the brain, and the internal memory state variables in our model can be seen as analogous to epigenetic states or memory states in the brain. Furthermore, as in biological systems, we use a generative encoding (a string coding for a series of conditional rules, similar to a DNA sequence coding for conditionally expressed gene regulatory networks) and evolve our system using mutation and crossover. One departure in our setup from biological reality, however, was that we used genetically homogeneous teams, as is common in evolutionary swarm robotics [58], but different from the situation in most social insects, where sexual reproduction tends to be the norm. This choice was made because homogeneous groups combined with team-level selection has been shown to be the most efficient approach to evolve tasks that require coordination [28]. Nevertheless, this setup can still be considered analogous to the genetically identical cells of multicellular organisms [59] or the clonal societies of some asexually reproducing ants [60] that both display complex forms of division of labor.

We executed a total of 22 evolutionary runs on a computer cluster, of which we used 100 to 200 nodes in parallel. The number 22 was chosen to meet the total amount of computational resources we had at our disposal (3 months of cluster time) and was statistically speaking more than adequate. All evolutionary runs were carried out for 2,000 generations using 100 groups of 4 robots and were each evaluated 3 times. This relatively small number of robots was chosen to limit the computational burden of the evolutionary runs. Nevertheless, we also verified if the evolved controllers could be scaled to larger teams of 20 robots each. In this case, the foraging arena was scaled in direct proportion with the number of robots. We used single-point crossover with crossover probability 0.3 and mutation probability 0.05. We chose a generational replacement with 5% elitism, in order to exploit parallel evaluation of multiple individuals on a computer cluster. We used roulette-wheel selection, that is, the probability to select a given genotype was proportional to its fitness relative to the average fitness of all genotypes in the population. As fitness criterion we used group performance, measured as the total number of items retrieved to the nest over a period of 5,000 seconds. During post-evaluation, this same fitness criterion was used to evaluate the evolved controllers. We also assessed the average absolute linear speed of the robots along the long axis of the arena, measured as a percentage of the theoretical maximum speed, and the degree of task specialization, measured as the proportion of items that were retrieved through the action of multiple robots (i.e. by task specialists).

## Results

### Evolution of task-partitioning from pre-adapted building blocks

In the first set of simulations, we assumed that each robot could specialize for life to one among the three possible preexisting behavioral strategies required for task partitioning, dropper, collector and generalist, and determined the optimal mix between the three strategies based on an exhaustive search of the full fitness landscape (Fig 1B and 1D). These simulations were performed both in a flat and a sloped environment. As proposed for natural systems [7,43,48], our a priori hypothesis was that task partitioning would be favored particularly in the sloped environment, and that maximal group performance would be achieved when some robots would specialize in dropping items in a cache and others in collecting items from the cache. This is because, in such an environment, some of the robots would be able to avoid the costly traversal of the slope area (i.e. avoid switching costs) and because gravity could also help to move items across the slope, thereby resulting in more economical transport (Fig 1).

**Fig 3:**
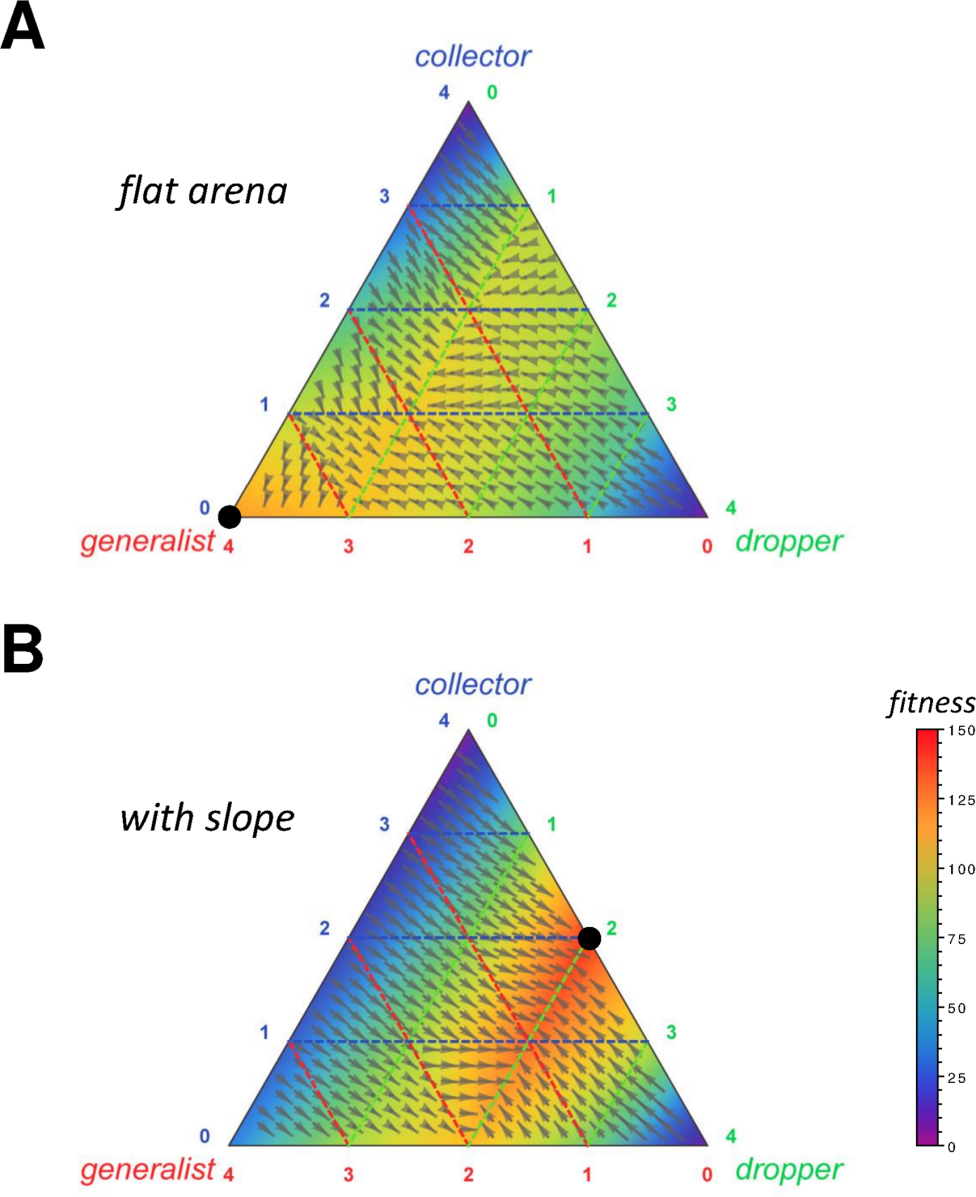
Optimal group composition in 4 robot teams using pre-adapted dropper, collector or generalist foraging strategies (cf. hand-coded rules shown in S1 Table). Ternary plots show group performance (total number of items retrieved to the nest over a period of 5,000 simulated seconds averaged over 10 simulation runs, color coded) as a function of the number of collectors (blue), droppers (green) and generalist foragers (red) in the 4 robot teams (black dot = optimum). In a flat environment **(a)**, teams of generalist foragers achieve optimal performance (cf. S2 Video), whereas in a sloped arena **(b)**, a mix of 2 droppers and 2 collectors is most optimal (cf. S1 Video). Both of these optima are global attractors in their respective fitness landscapes (cf. vectors which represent the phase portrait).

Examination of the obtained fitness landscapes reveals that there was one globally attracting optimum in each of the two environments considered (Fig 3A and 3B). As expected, this optimum involved task partitioning in the sloped environment (Fig 3B), with a mix of 50% droppers and 50% collectors being most efficient, but only generalist foraging in the flat environment (Fig 3A, S1 and S2 Videos). In addition, our fitness landscape analysis showed that when pre-adapted behavioral building blocks can be used in the evolutionary process, the fitness landscape tends to be very smooth, thereby making task specialization easily evolvable, without the risk of the system getting trapped in suboptimal local optima. It should also be noted that in our setup, the absolute group performance was significantly higher (*t*-test, *t* = -16.6, d.f. = 18, *p*<10^−11^) in the sloped environment (144.1 ± 4.3 *S*.*D*. items collected in 5,000 s, *n* = 10) than in the flat one (120.2 ± 1.4 *S*.*D*. items collected in 5,000 s), due to the fact that in the first case, gravity helped to move the items towards the source.

### Evolution of task-partitioning from first principles

**Fig 4:**
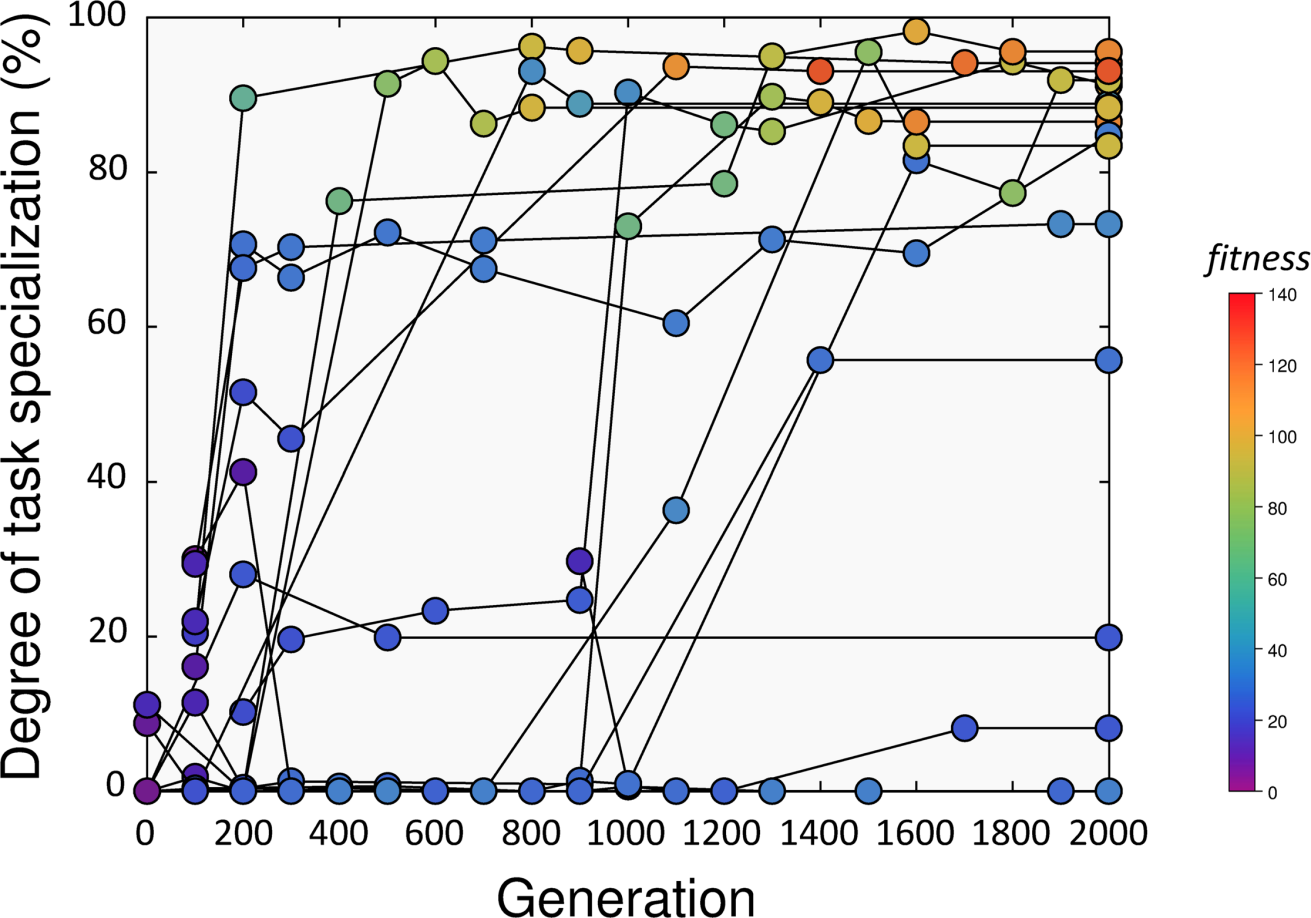
Group performance and degree of task specialization displayed by 4 robot teams over subsequent generations for each of the 22 evolutionary runs. The degree of task specialization (Y axis) is measured as the proportion of items retrieved by more than one robot over the total number of items retrieved. The group fitness (color-coded) is the total number of items retrieved to the nest over a period of 5,000 simulated seconds averaged over 2 simulation runs. The degree of task specialization and the group fitness of the best evolved controller in each generation is shown over subsequent generations for each of the 22 evolutionary runs. High task partitioning was evolutionarily stable, since any transition to high task partitioning never reverted back to generalist foraging in later generations. Some controllers, however, did not evolve task partitioning as a result of being trapped in local optima.

**Fig 5:**
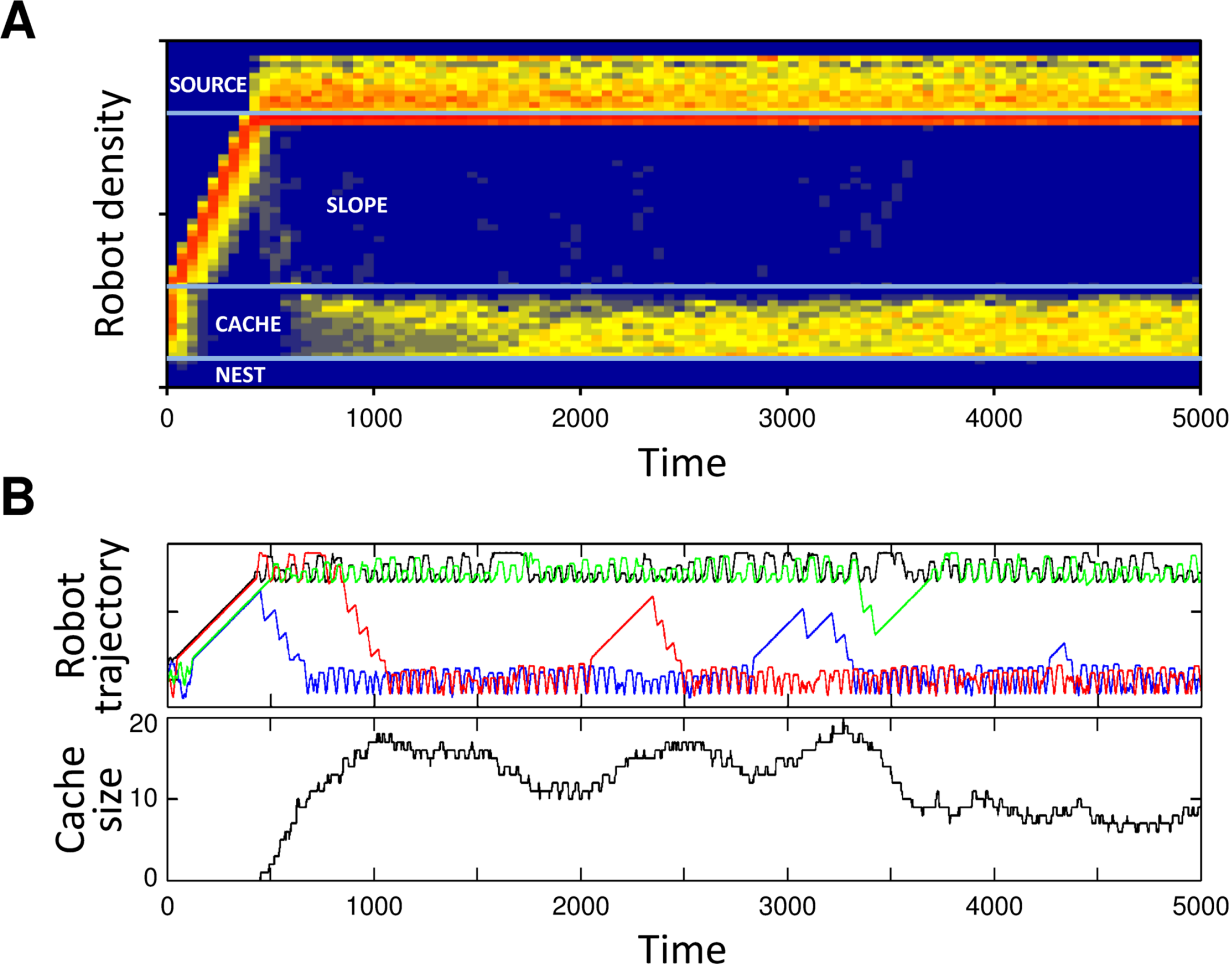
Self-organized task specialization and task allocation displayed by a controller evolved from first principles using Grammatical Evolution (cf. S3 Video and evolved rules shown in S2 Table). (a) Robot densities in the experimental arena of as a function of time (average of 30 runs). Despite having identical controllers, robots segregate quickly between the source and cache areas, thereby avoiding the costly traverse of the slope. (b) Robot trajectory on the arena and cache size in a typical evaluation run. All robots first move to the source to collect items, but after 500–1000 s into the simulation, the robot teams self-organize to have two droppers pushing items off the slope and two robots collecting items from the cache, without these tasks having been explicitly rewarded during the evolutionary runs.

In a second set of experiments, we used GESwarm [41] to evolve task specialization and task allocation entirely de-novo, starting only from basic, low-level behavioral primitives (see Materials and Methods). Surprisingly enough, these evolutionary experiments demonstrated that task partitioning and fully self-organized task specialization and task allocation could also emerge entirely from scratch by selecting purely on overall group performance (number of items retrieved to the nest). In particular, our experiments show that in 59% (13 out of 22) of the runs, the majority of the items were retrieved by the robots in a task-partitioned way in the final evolved controller obtained after 2,000 generations (Fig 4, S3 and S4 and S5 Videos). In these cases, most of the items were first dropped by one robot and later picked up by another one. In contrast to the case with predefined behavioral strategies, however, the task specialization that was seen in these controllers did not entail fixed roles, but instead was characterized by a dynamic allocation in response to the size of the cache. An example of a controller (nr. 2) displaying such behavior is shown in S3 Video, where the majority of the robots first exploit the source to act as droppers, but then move down the slope as the cache fills up to act as collectors (the evolved rules of this controller are shown in S2 Table). The robots shown in these simulations used simple probabilistic rules to switch from the source to the cache area, while the cache itself was exploited to switch from the cache area back to the source area. We observed that the latter mechanism was also very simple and based on stigmergy, i.e. robots would collect from the cache whenever objects were found on the way, but would continue all the way to the source when cache items were not encountered. Thanks to these simple mechanisms, the robots could dynamically switch roles in response to the size of the cache. The same adaptive specialization dynamics are apparent in Fig 5A, where the density of the robot positions across the arena is shown across the 30 runs used for post-evaluation of the same controller, and in Fig 5B, which displays the individual trajectories of the four robots in a typical evaluation run (the spatial segregation and robot trajectories for all other evolved controllers are shown in S1 Fig).

That such self-organized task specialization and task allocation could evolve from first principles by selecting purely on group performance is significant, given that we started from a random controller that barely achieved any foraging during the first few generations (Fig 4, S1 Video). As in the case without pre-adapted building blocks that we considered in the previous section, also here the presence of a slope was sufficient for the evolution of task partitioning. Indeed, when we conducted the very same experiments in a flat environment, none of the controllers evolved task partitioning and generalist foraging was the favored strategy [41].

Significantly, the evolved rules for both generalist foraging [41] and task partitioned object retrieval scaled very well also to larger teams of robots. An example is shown in S1 Video, where one of the evolved controllers from a 4 robot team is implemented in a team of 20 robots. In this case, the achieved group performance scaled almost perfectly with the increase in group size (457 ± 72 *S*.*D*. in the 20 robot team vs. 103 ± 24 *S*.*D*. in the 4 robot one). Excellent scalability properties were also shown by the fact that for the 8 best evolved controllers, the performance ratio of the rules when implemented in the 20 robot teams relative to that in the 4 robot ones in which the rules were first evolved was very close to the expected linear scaling factor of 5 (4.4, *S*.*D*. 0.14, see S3 Table).

**Fig 6:**
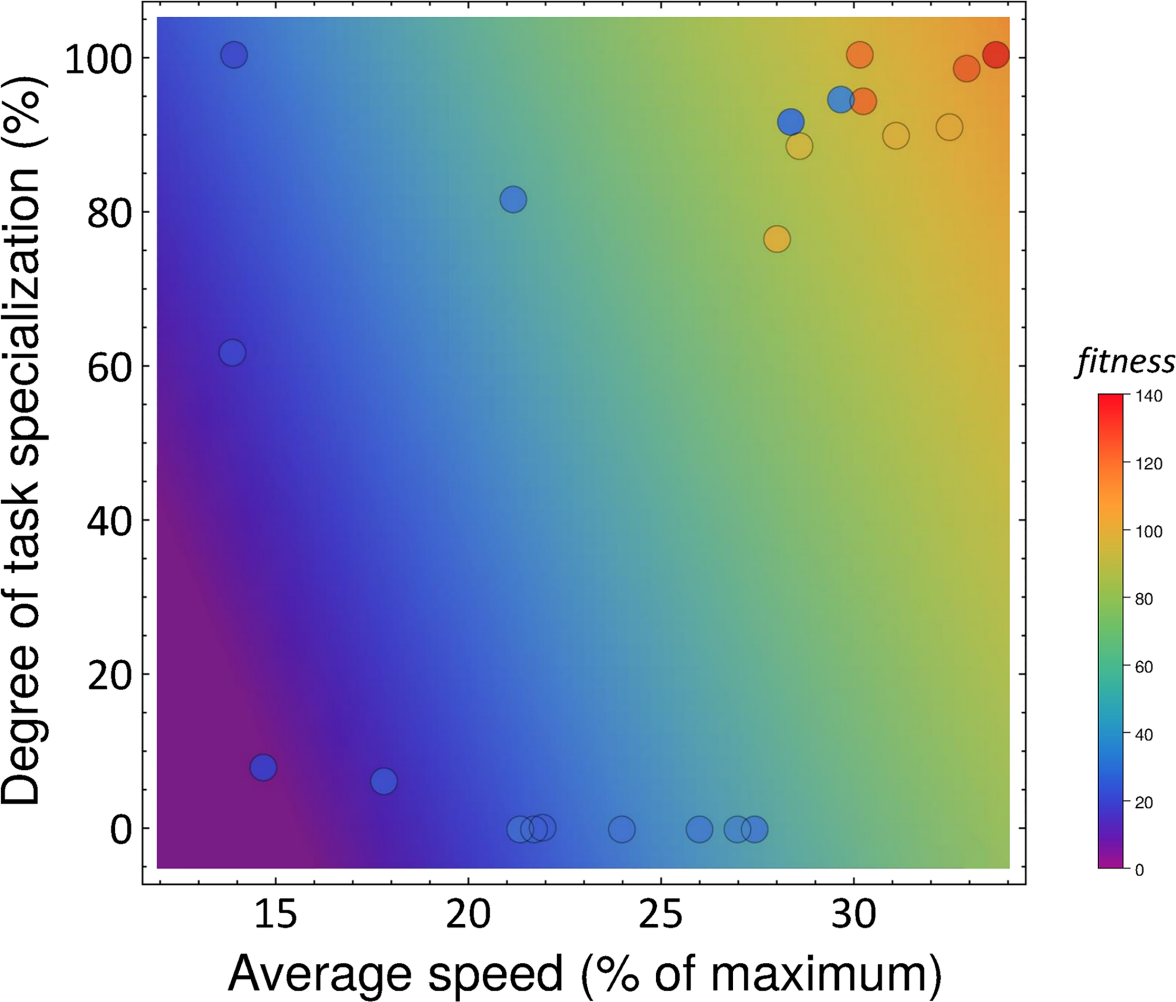
The effect of the degree of task specialization (Y axis, proportion of items retrieved through the action of multiple robots) and average linear speed (absolute average linear speed of the robots along the long axis of the arena as a percentage of the theoretical maximum speed) on the fitness performance of the 22 controllers evolved from first principles. A high degree of task partitioning and high speed significantly increased group fitness (color code, multiple regression analysis: *p*<0.01 and *p<*10^−5^; color gradient represents the best-fit plane, average of 30 runs).

Although the lack of fixed roles precluded an analysis in terms of behavioral roles similar to that presented in the section above, it turned out that both increased amounts of task partitioning and higher average linear speeds significantly increased group fitness (multiple regression analysis, *p*<0.01 and *p*<10^−5^, respectively, *n* = 22, Fig 6). In fact, all 8 evolved controllers displaying a high group performance (top 35%, >ca. 100 items collected) had very high levels of task partitioning (92% ± 0.08 *S*.*D*. of all items retrieved in a task partitioned way) and achieved a high average linear speed (31% ± 0.6% *S*.*D*. of the theoretical maximum). Significantly, out of these 8, the performance of the best evolved controller (135 ± 14 *S*.*D*., *n* = 30 items retrieved) was not significantly different from the optimal 2 dropper-2 collector mix obtained in the experiment using hand-coded behavioral strategies above (144.1 ± 4.3 *S*.*D*., *t*-test, *t* = 2.01, d.f. = 38, *p* > 0.05). Among these 8 best controllers, between 4 and 11 rules were used to switch between the different allowed behaviors and instantaneous actions (cf. evolved rules shown in S2 Table). Interestingly, in 3 of these best controllers, the rules employed as a precondition a memory state variable that was increased or decreased as a result of some of the actions performed in other rules. In principle, the use of these state variables could have allowed for the evolution of mechanisms akin to the response threshold model, which has been extensively used in studies on division of labor [4,9,10,16]. Nevertheless, none of our controllers succeeded in evolving this particular mechanism, and task allocation instead appeared to be based purely on probabilistic and stigmergic switching, as explained above.

A detailed analysis of the fitness and behavior of the final evolved controllers demonstrated that there was one global optimum characterized by a high level of task partitioning and high linear speed (Fig 6). Nevertheless, some runs were trapped in suboptimal regions of the search space. For example, some controllers merely displayed generalist foraging, which was suboptimal in our setup (Fig 6, bottom right points). Similarly, other controllers were characterized by defective locomotory skills, even if some actually achieved task partitioning (Fig 6, left blue points). Finally, two evolved controllers were characterized by a high degree of task partitioning and a decent speed, but nevertheless had low overall performance due to the use of a suboptimal dropping strategy, characterized by a continuous dropping and picking up in all the regions of the environment, which affected performance but not speed and degree of task partitioning (Fig 6, two blue points in the upper-right corner). These outliers, however, did not change the fact that fitness was strongly correlated with both the degree of task specialization and the linear speed of the robots.

Despite the variation in performance of the final evolved controllers, an analysis of fitness and degree of task partitioning over the course of the evolutionary runs (Fig 4) clearly demonstrates that high task partitioning was evolutionarily stable, since any transition to high task partitioning never reverted back to generalist foraging in later generations.

## Discussion

One of the unsolved mysteries in biology is how a blind process of Darwinian selection could have led to the hugely complex forms of sociality and division of labor as observed in insect societies [4]. In the present paper, we used simulated teams of robots and artificially evolved them to achieve maximum team performance in a foraging task. Remarkably, we found that, as in social insects, this could favor the evolution of a self-organized division of labor, in which the different robots automatically specialized into carrying out different subtasks in the group. Furthermore, such a division of labor could be achieved merely by selecting on overall group performance and without pre-specifying how the global task of retrieving items would best be divided into smaller subtasks. This is the first time that a fully self-organized division of labor mechanism could be evolved entirely de-novo. Overall, these findings have several important implications. First, from a biological perspective, they yield novel evidence for the adaptive benefits of division of labor and the environmental conditions that select for it [4], provide a possible mechanistic underpinning to achieve effective task specialization and task allocation [4] and provide possible evolutionary pathways to complex sociality. Second, from an engineering perspective, our nature-inspired evolutionary method of Grammatical Evolution clearly has significant potential as a method for the automated design of adaptively behaving teams of robots.

In terms of the adaptive benefits of division of labor and the environmental conditions that select for it, our results demonstrated that task partitioning was favored only when features in the environment (in our case a slope) could be exploited to achieve more economic transport and reduce switching costs, thereby causing specialization to increase the net efficiency of the group. Previous theoretical work has attributed the evolution of task specialization to several ultimate factors, some of which are hard to test empirically [61]. Duarte et al. [4], for example, reviewed modeling studies that showed that the adaptive benefits of a behaviorally-defined division of labor could be linked to reduced switching costs between different tasks or locations in the environment, increased individual efficiency due to specialization, increased behavioral flexibility or reduced mortality in case only older individuals engage in more risky tasks (“age polyethism”). Out of these, there is widespread agreement on the role of switching costs and positional effects as key factors in promoting task specialization [4,10,47,62], and our work confirms this hypothesis. Indeed, in our set-up, task partitioning greatly reduced the amount of costly switching required between environmental locations. Furthermore, our work also confirms the economic transport hypothesis, i.e. that task partitioning results in more economical transport, which in our case was due to the fact that gravity acted as a helping hand to transport the items. Previously, this hypothesis had also found significant empirical support [7,43,46,48], e.g. by the fact that in leafcutter ants, species that collect leaves from trees tend to engage in task partitioned leaf retrieval, whereas species living in more homogeneous grassland usually retrieve leaf fragments in an unpartitioned way, without first dropping the leaves, particularly at close range to the nest [43,49].

A surprising result in our evolutionary experiments was that adaptive task specialization was achieved despite the fact that the robots in each team all had identical controllers encoded by the same genotype. This implies that a combination of individual experience, stigmergy and stochastic switching alone were able to generate adaptive task specialization, akin to some of the documented mechanisms involved in behavioral task specialization in some asexually reproducing ants [63] and in cell differentiation in multicellular organisms and clonal bacterial lineages [59,64,65]. The choice of using homogeneous, clonal groups of robots with an identical morphology precluded other mechanisms of division of labor observed in nature from evolving, based, for instance, on morphological [4,12] or genetic [4] role specialization. Such mechanisms, however, could be considered in the future if one allowed for genetically heterogeneous robot teams [28] or evolvable robot morphologies. Lastly, the grammar we used did not specifically allow for recruitment signals to evolve, such as those observed in leafcutting ants, where both trail pheromones and stridulation are used as mechanisms to recruit leaf cutters [66,67], or the ones in honeybees, where the tremble dance is used to regulate the balance between number of foragers and nectar receivers inside the colony [68,69]. Nevertheless, including low-level primitives for communication behavior into the grammar, which we plan to do in future work, would readily allow for the evolution of such mechanisms, and would likely boost the performance of the evolved controllers even further (cf. [26,27]).

In terms of the mechanisms of task specialization and task allocation evolved, our work is important in that it alleviates one of the limitations of existing models on the evolution of task specialization, namely, that they normally take pre-specified subtasks and an existing task allocation model (e.g. the response threshold model) as point of departure [4], thereby greatly constraining the path of evolution. Our work is an important cornerstone in establishing, to the best of our knowledge, the first model that bridges the gap between self-organization and evolution without significantly constraining the behavioral strategies and coordination mechanisms that can be obtained to achieve optimal task specialization and task allocation. In fact, compared to other previous studies on evolution of task specialization [47,62,70–72], our work is the first to consider non-predefined sub-tasks that could evolve de-novo and combine into complex individual behavioral patterns.

Although our experiments demonstrate that division of labor and behavioral specialization in teams of identical robots could evolve in both the scenarios we considered, fitness landscape analyses showed that optimal task allocation could be achieved more easily if optimized behaviors capable of carrying out the different subtasks were available as pre-adapted behavioral building blocks. This leads us to suggest that when building blocks are solidified in earlier stages of the evolution, complex coordination strategies such as task specialization are more likely to evolve as the fitness landscape becomes smoother and also easier to explore due to its greatly reduced size. In addition, it brings further support for the hypothesis that, in nature, the evolution of division of labor in social groups and other transitions in the evolution of sociality also tends to be based on the co-option of pre-existing behavioral patterns, as opposed to requiring the de-novo evolution of many entirely new social traits [17]. Our results, therefore, match and can be integrated with available evidence with respect to the importance of preadaptations in the origin of advanced forms of sociality [2,17–22,73]. For example, reproductive division of labor and worker task specialization are thought to be derived from mechanisms that initially regulated reproduction and foraging in solitary ancestors [17,20–22], sibling care is thought to be derived from ancestral parental care [19], and reproductive altruism (i.e., a sterile soma) in some multicellular organisms evolved via the co-option of a reproduction-inhibiting gene expressed under adverse environmental conditions [73]. Furthermore, it confirms other studies that have examined the building block hypothesis with various digital systems, for example in the context of genetic algorithms [74], evolution of single robot morphologies [75] and the open-ended evolution of simple computer programs [76].

From an engineering perspective our study is the first to achieve a complex form of division of labor using an evolutionary swarm robotics approach, and the first to use the method of Grammatical Evolution to evolve complex, non-trivial behavioral patterns. This result is novel in the field of evolutionary swarm robotics, where, few exceptions aside, most studies have used non-incremental and non-modular approaches, e.g. based on monolithic neural networks [38,77]. In fact, previously, the only other studies which evolved a rudimentary task allocation in swarms of robots were those of Tuci *et al*. [78], which used a neural network controller combined with a fitness function favoring a required preset task allocation [78], of Duarte *et al*. [40], which used evolved neural network controllers capable of carrying out particular subtasks, which were then combined with a manually engineered decision tree, and the work of refs. [79–81], which used open-ended evolution and a simplified robotic scenario to evolve heterogeneous behaviors for collective construction [79,80] and pursuit [81] tasks in presence of a pre-specified set of three sub-tasks. Typically, the behavioral complexity that could be reached in these artificial neural network-based studies was quite limited, making the evolution of self-organized task specialization in homogeneous groups out of reach for these methods. In fact, the evolution of self-organized task specialization would clearly require a non-standard neural network approach, involving recurrent neural connections to keep track of the internal state (e.g. the current direction of motion to be able to perform phototaxis), a mechanism to achieve modularity and a mechanism to switch stochastically between these modules. Extending the neural network approach used in evolutionary swarm robotics to this level of complexity would be an interesting task for the future. Other studies on task allocation and task partitioning in swarm robotics typically used traditional, manually engineered approaches [82–88] (reviewed in [89]). All these methods are significantly less general than ours, given that we used a nature-inspired automatic design method with a single fitness criterion, group performance, without any pre-engineered decision-making mechanisms, and simultaneously evolved a self-organized task decomposition and task allocation mechanism as well as optimized behaviors to carry out each of the evolved subtasks. We therefore believe that GESwarm and grammatical evolution will play a key role in the future of evolutionary swarm robotics.

In conclusion, our work and the results we obtained are therefore important both to explain the origin of division of labor and complex social traits in nature, as well as to advance the field of evolutionary swarm robotics, as we showed that the novel methodological and experimental tools we developed were able to synthetize controllers that were beyond the level of complexity achieved to date in the field.

## Supporting Information

S1 Fig The different types of dynamics displayed by all 22 controllers evolved from first principles using Grammatical Evolution (cf. S4 and S5 Videos and evolved rules shown in S2 Table). The figures are ordered based on performance, from the best to the worst. (a) Robot densities in the experimental arena as a function of time (average of 30 runs). (b) Robot trajectory on the arena and cache size in a typical evaluation run.
(PDF)

S1 Table Rules used to encode the dropper, collector and generalist foraging strategies in the experiments with pre-adapted building blocks. Most of the rules are used by more than one behavioral building block (rules R1 and R4-R6 are used by droppers, rules R2-R3, R5 and R7-R8 are used by collectors and rules R1, R4-R5 and R7-R8 are used by generalists). For each rule: the first row contains the list of preconditions, each denoted by the syntax *P*_*NAME*_ = *True|False* where *NAME* is the intuitive name of the precondition; the second row contains the list of fine-grained behavioral building blocks (*B*_*RANDOM_WALK*,_ *B*_*PHOTOTAXIS*,_ *B*_*ANTI-PHOTOTAXIS*_, c.f. Materials and Methods); the remaining rows contain the list of actions (one per row), where the first column indicates the type of the action (*A*_*B*_ are actions that change the currently-executed behavior, while *A*_*IS*_ are all other actions), the second column indicates the execution probability, and the third column indicates the effect of the action (either the new behavior to switch to in case of *A*_*B*_ or the new value of the internal state *IS*_*NAME*_ in case of *A*_*IS*_). Memory states were set as follows: *P*_*STAY_DOWN*_ = *True* and *P*_*STAY_UP*_ = *False* for collectors, *P*_*STAY_DOWN*_ = *False* and *P*_*STAY_UP*_ *= True* for droppers and *P*_*STAY_DOWN*_ = *False* and *P*_*STAY_UP*_ = *False* for generalists.
(PDF)

S2 Table Rules evolved via Grammatical Evolution in the 22 evolutionary runs. Controllers are sorted from high to low group performance.
(PDF)

S3 Table Performance of the 22 evolved controllers and degree of task partitioning observed in the 4 robot teams and in the 20 robot teams used during post-validation. Controllers are sorted from high to low group performance.
(PDF)

S1 Video Video of the optimal behavior displayed by the controller with pre-adapted building blocks in the sloped environment. In this case, an allocation of 50% droppers and 50% collectors resulted in maximal group performance.
(MP4)

S2 Video Video of the optimal behavior displayed by the controller with pre-adapted building blocks in the flat environment. In this case, an allocation of 100% generalist foragers resulted in maximal group performance.
(MP4)

S3 Video Example of task partitioning behavior evolved during evolutionary run number 2. From initially random behavior, the robots first evolve generalist foraging after 500 generations. Subsequently, after 500 more generations, the robots evolve task partitioning, which gets further perfected over the following 1000 generations. We conclude by showing how the controller evolved in a 4 robot team scaled up when tested in a swarm of 20 robots. The full HD video is available at https://www.youtube.com/watch?v=8mlHXcCNzjg.
(AVI)

S4 Video Behavior displayed by the first best 11 evolved controllers. Videos are sorted from high to low group performance.
(ZIP)

S5 Video Behavior displayed by the next best 11 evolved controllers. Videos are sorted from high to low group performance.
(ZIP)
