# Correction: Evolution of Self-Organized Task Specialization in Robot Swarms

**DOI:** 10.1371/journal.pcbi.1004996

**Published:** 2016-06-16

**Authors:** The PLOS Computational Biology Staff

The funding statement for this article should read as follows:

“EF acknowledges the Fund for Scientific Research (FWO)–Flanders (grant 12N7515N). EF, AET and TW acknowledge the European Science Foundation "H2Swarm" program (http://www.esf.org/) and the KU Leuven (www.kuleuven.be) for the IDO-BioCo3 project and KU Leuven Excellence Center project PF/2010/007. EDG acknowledges the KU Leuven for the Grant F+/11/033. MD acknowledges the Fonds de la Recherche Scientifique—FNRS (F.R.S.–FNRS—www.fnrs.be) and the European Research Council (ERC—erc.europa.eu) ERC Advanced Grant “E-SWARM: Engineering Swarm Intelligence Systems” (grant 246939). AET acknowledges the Scientific and Technological Research Council of Turkey (Tubitak—www.tubitak.gov.tr/en) grant TUBITAK-2219. The funders had no role in the study design, data collection and analysis, decision to publish, or preparation of the manuscript.”
