# Supplementary material for: Evolution of Self-Organized Task Specialization in Robot Swarms
Source: PLoS Comput Biol. 2015 Aug 6;11(8):e1004273. doi: 10.1371/journal.pcbi.1004273 (PMC4527708; doi:10.1371/journal.pcbi.1004273)
Supplement: S1 Fig — The figures are ordered based on performance, from the best to the worst. (a) Robot densities in the experimental arena as a function of time (average of 30 runs). (b) Robot trajectory on the arena and cache size in a typical evaluation run. (PDF) [file pcbi.1004273.s001.pdf]

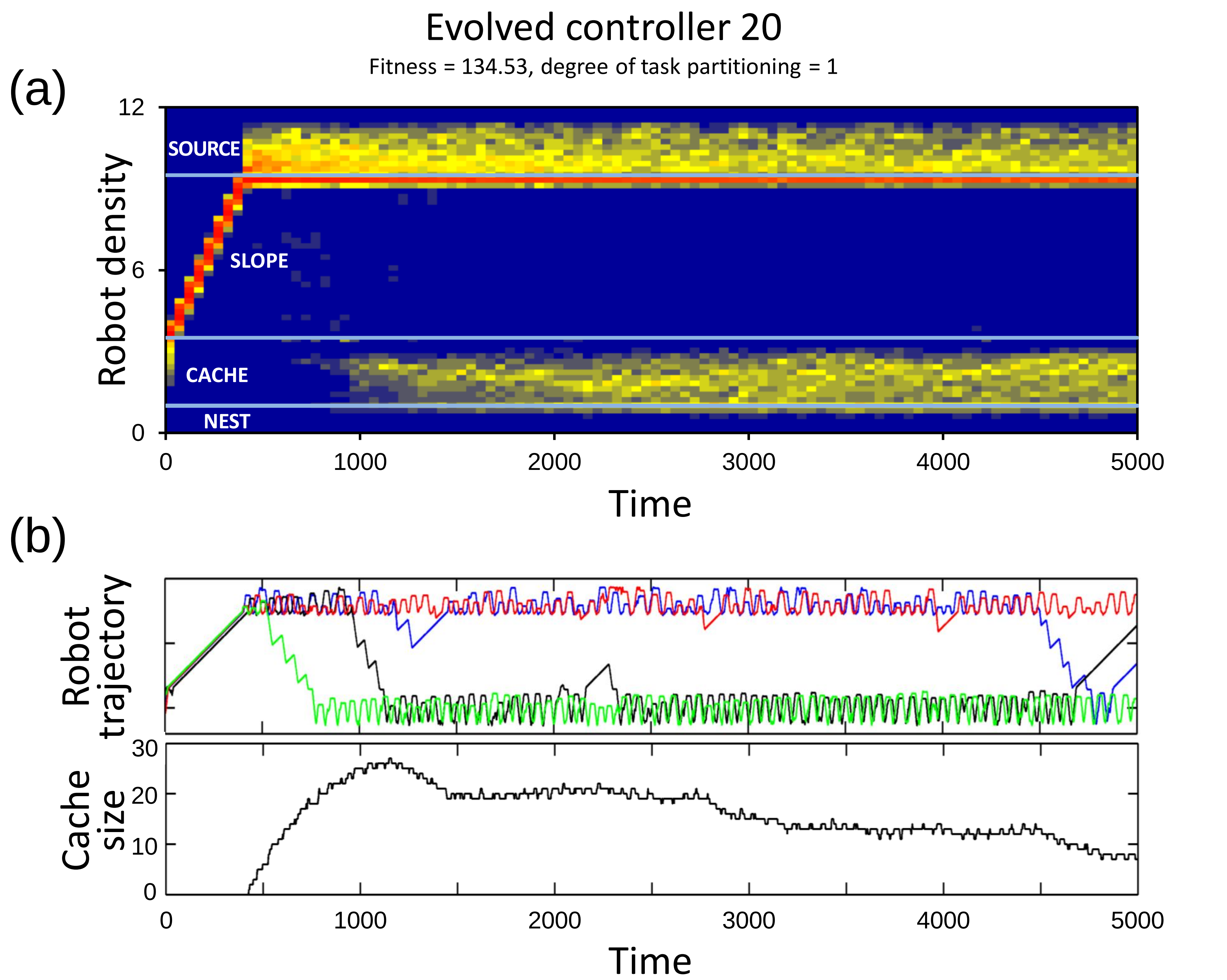

Evolved controller 20
Fitness = 134.53, degree of task partitioning = 1
(a)
Robot density
12
6
0
SOURCE
SLOPE
CACHE
NEST
0
1000
2000
3000
4000
5000
Time
(b)
Robot trajectory
Cache size
30
20
10
0
0
1000
2000
3000
4000
5000
Time

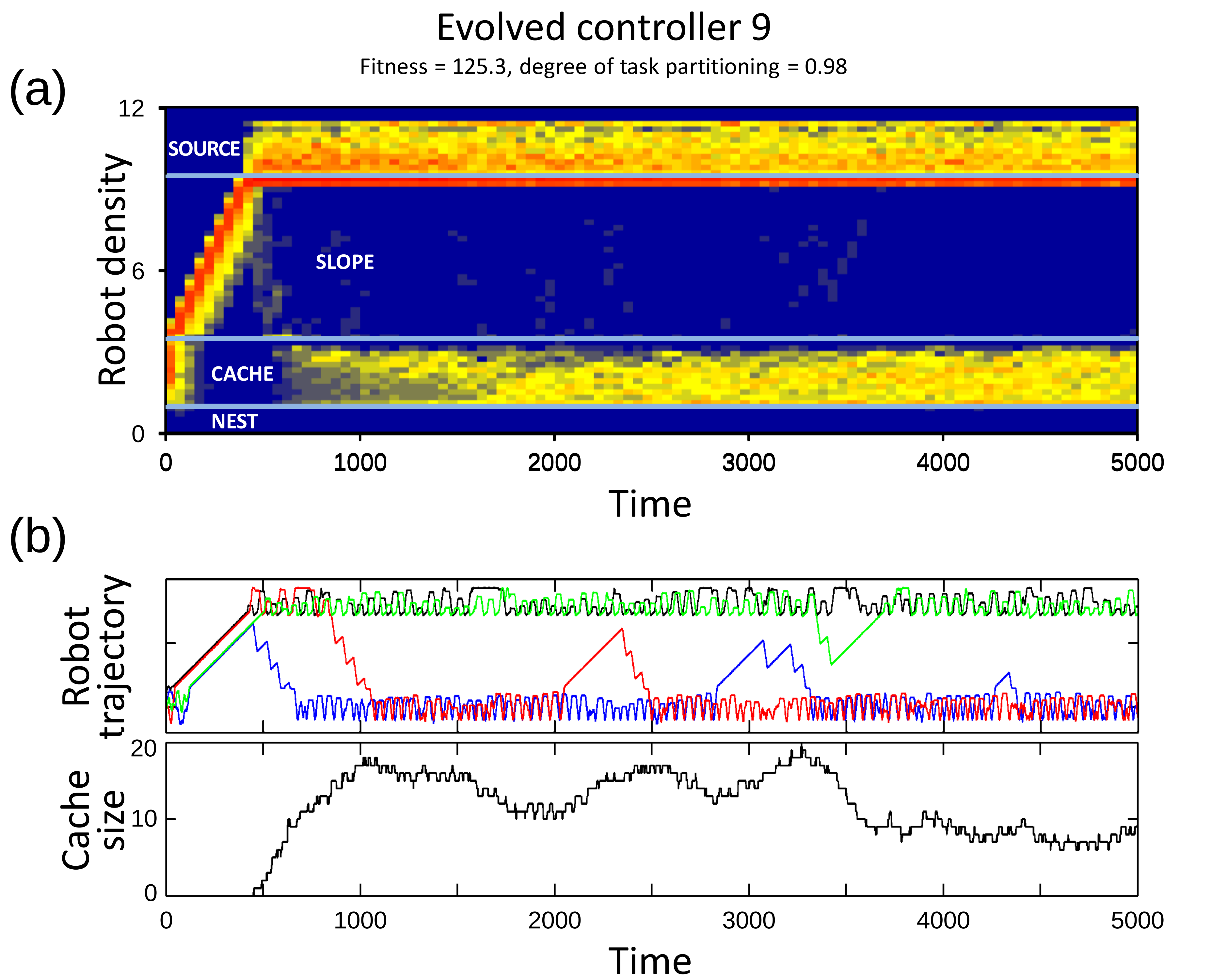

Evolved controller 9
Fitness = 125.3, degree of task partitioning = 0.98
(a)
Robot density
SOURCE
SLOPE
CACHE
NEST
Time
(b)
Robot trajectory
Cache size
Time

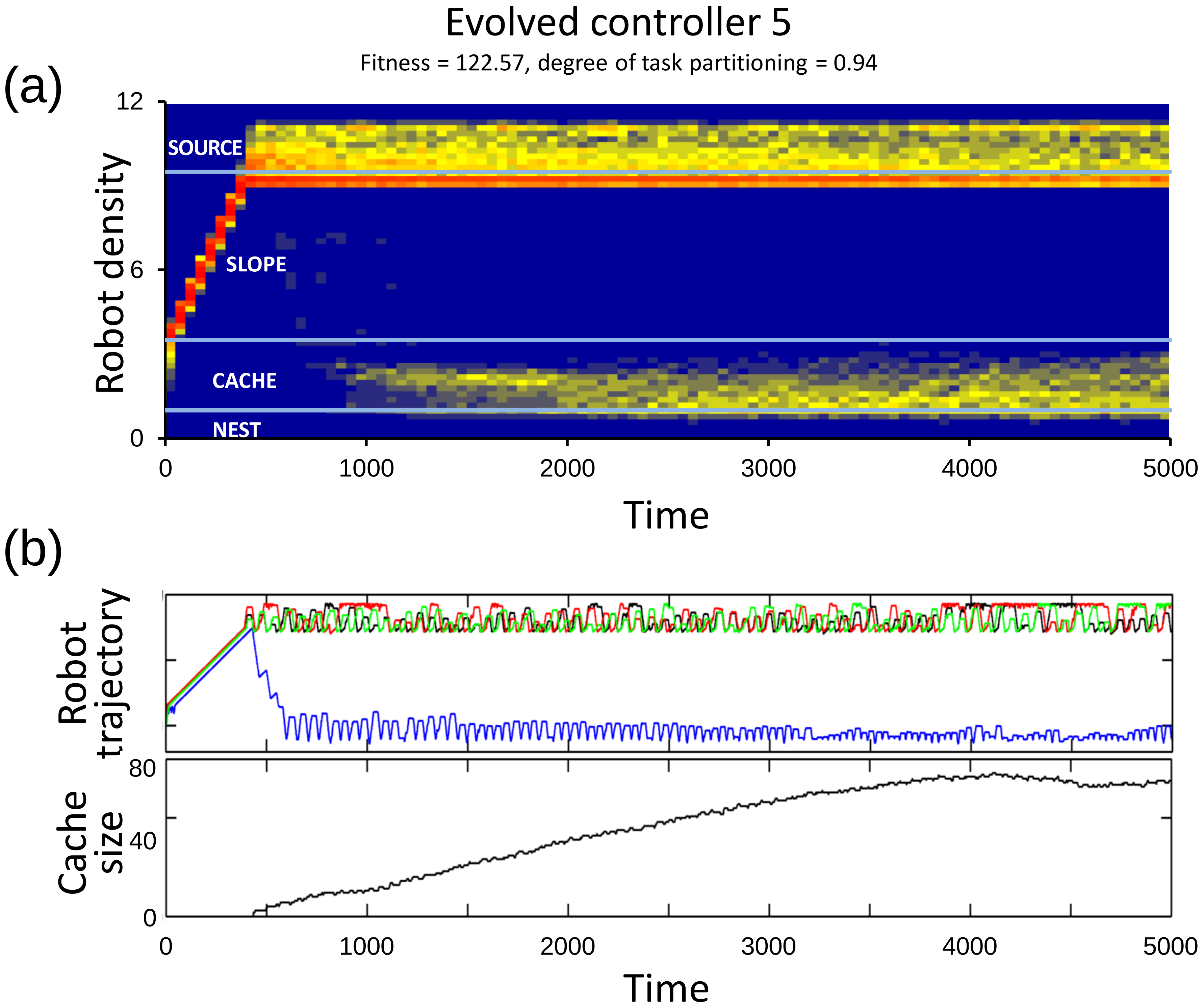

Evolved controller 5
Fitness = 122.57, degree of task partitioning = 0.94
(a)
Robot density
12
6
0
SOURCE
SLOPE
CACHE
NEST
0
1000
2000
3000
4000
5000
Time
(b)
Robot trajectory
Cache size
80
40
0
0
1000
2000
3000
4000
5000
Time

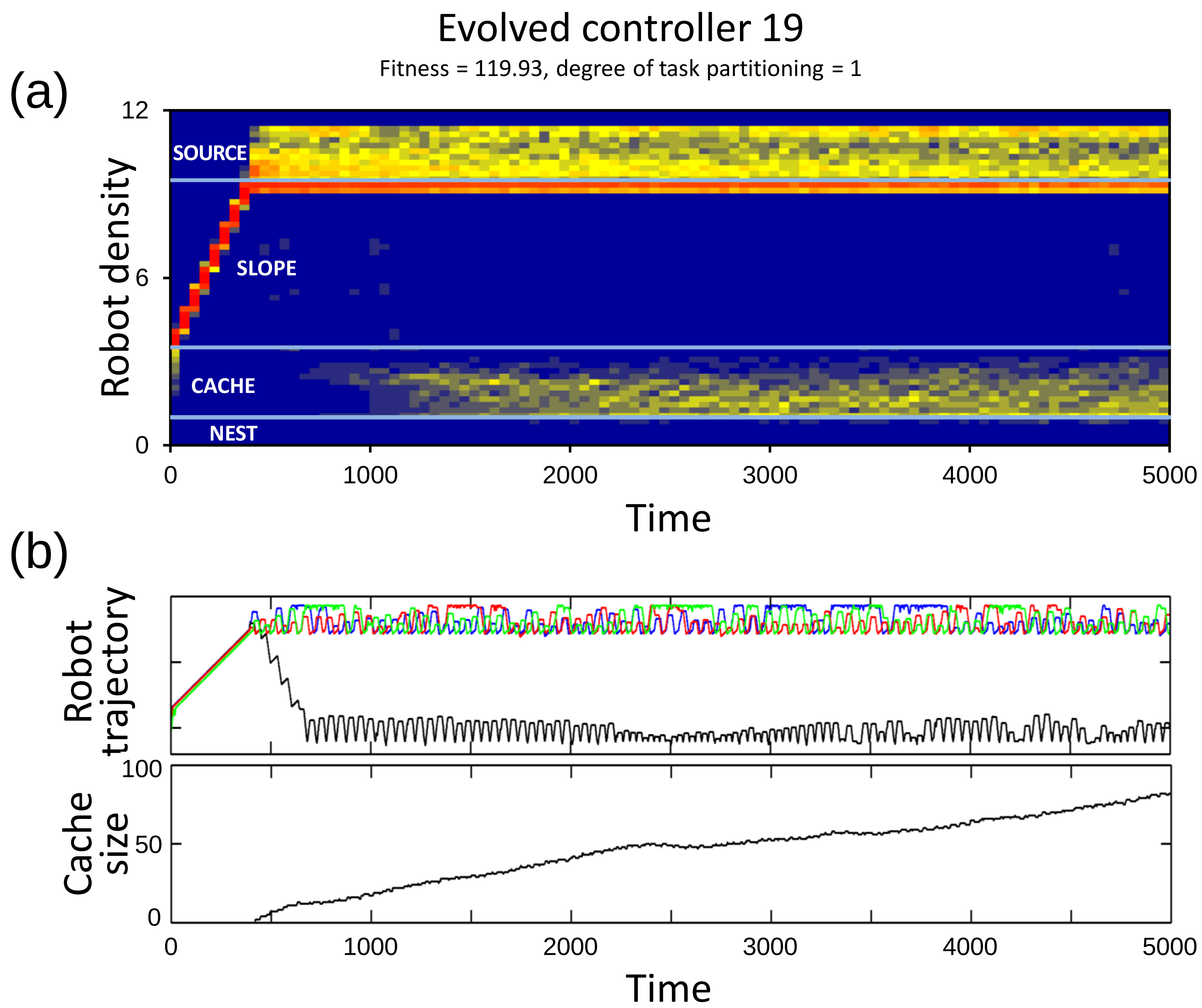
Evolved controller 19
Fitness = 119.93, degree of task partitioning = 1
(a)
Robot density
12
6
0
SOURCE
SLOPE
CACHE
NEST
0
1000
2000
3000
4000
5000
Time
(b)
Robot trajectory
Cache size
100
50
0
0
1000
2000
3000
4000
5000
Time

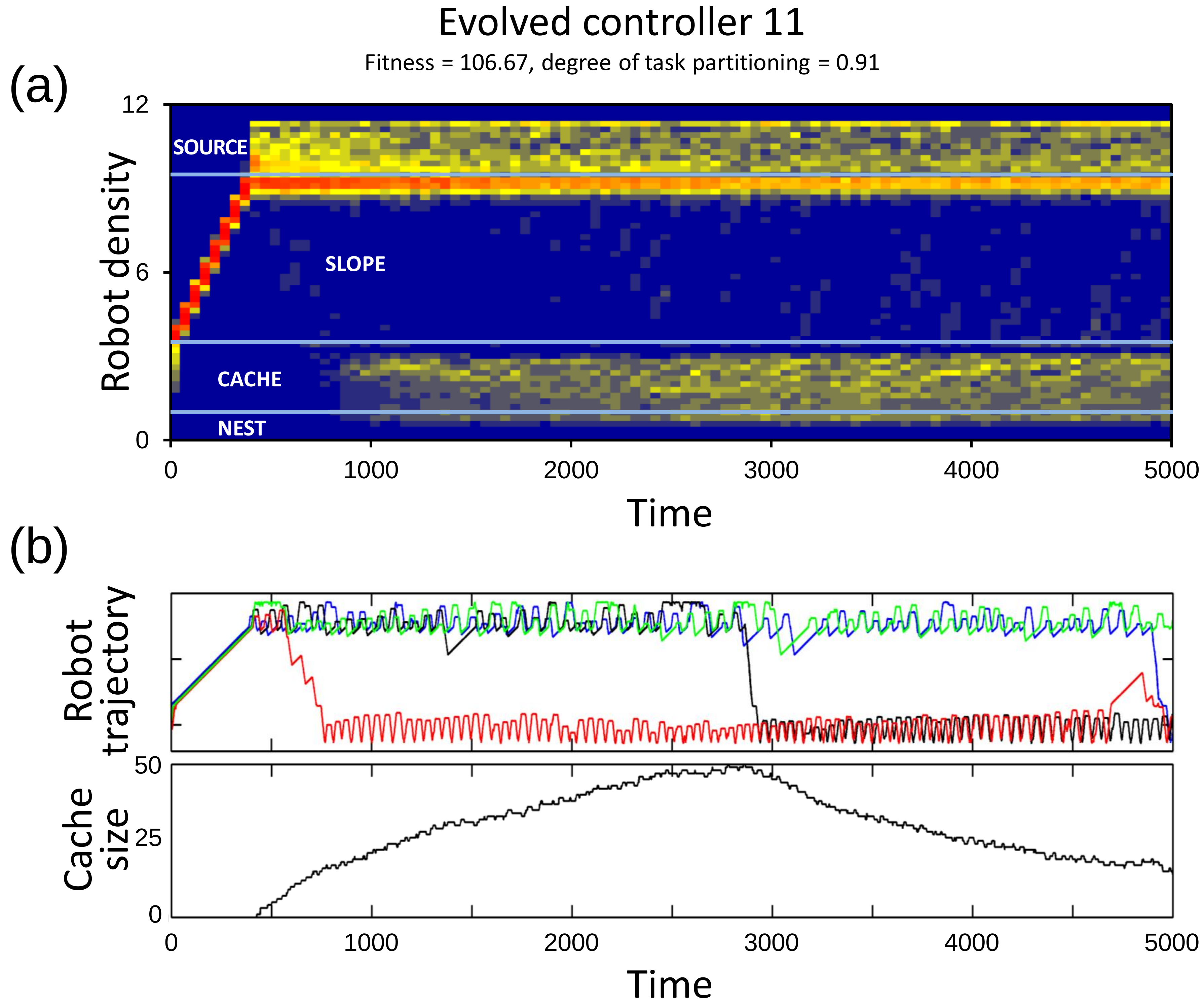

Evolved controller 11
Fitness = 106.67, degree of task partitioning = 0.91
(a)
Robot density
SOURCE
SLOPE
CACHE
NEST
12
6
0
0
1000
2000
3000
4000
5000
Time
(b)
Robot trajectory
Cache size
50
25
0
0
1000
2000
3000
4000
5000
Time

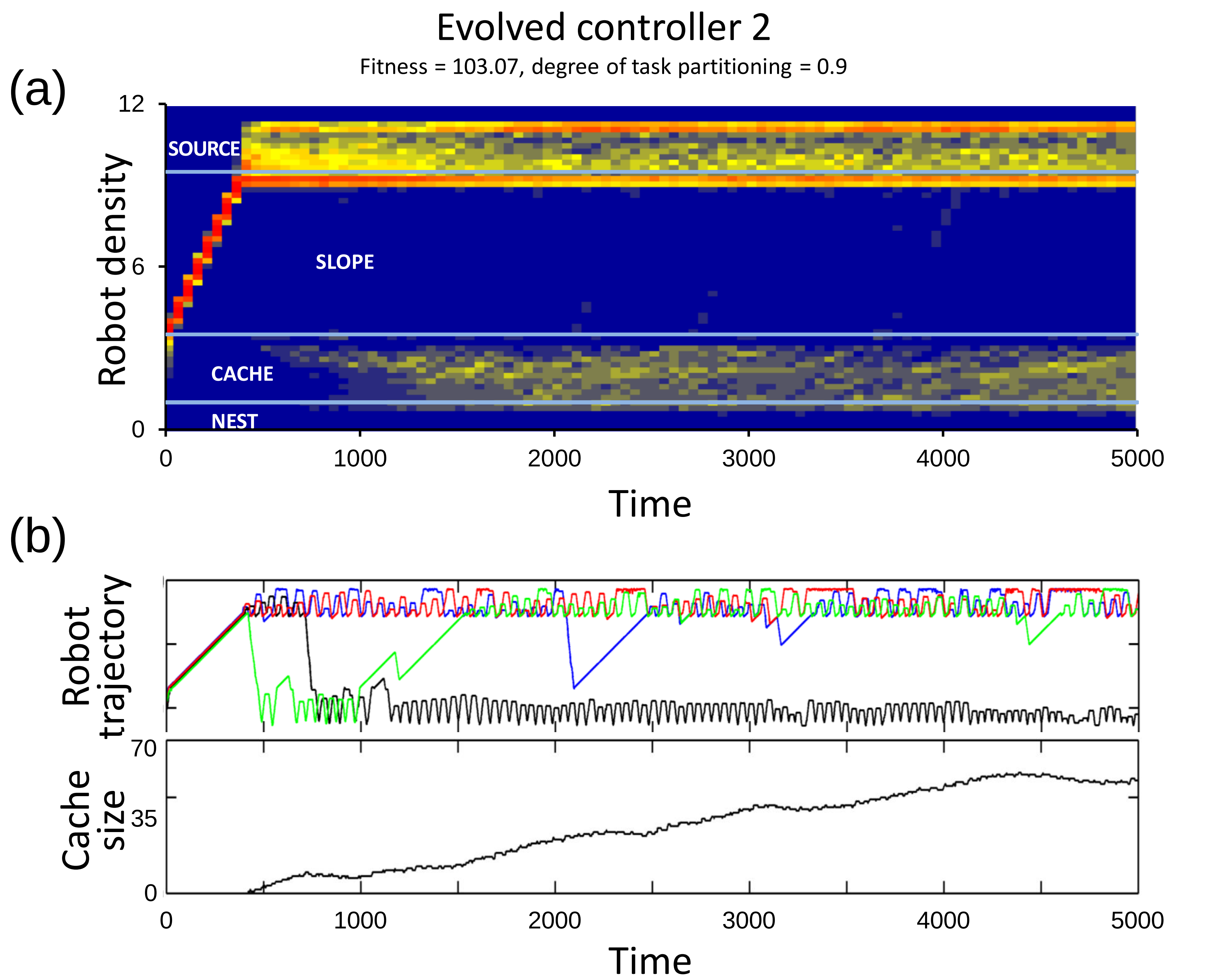

Evolved controller 2
Fitness = 103.07, degree of task partitioning = 0.9
(a)
Robot density
12
6
0
SOURCE
SLOPE
CACHE
NEST
0
1000
2000
3000
4000
5000
Time
(b)
Robot trajectory
Cache size
70
35
0
0
1000
2000
3000
4000
5000
Time

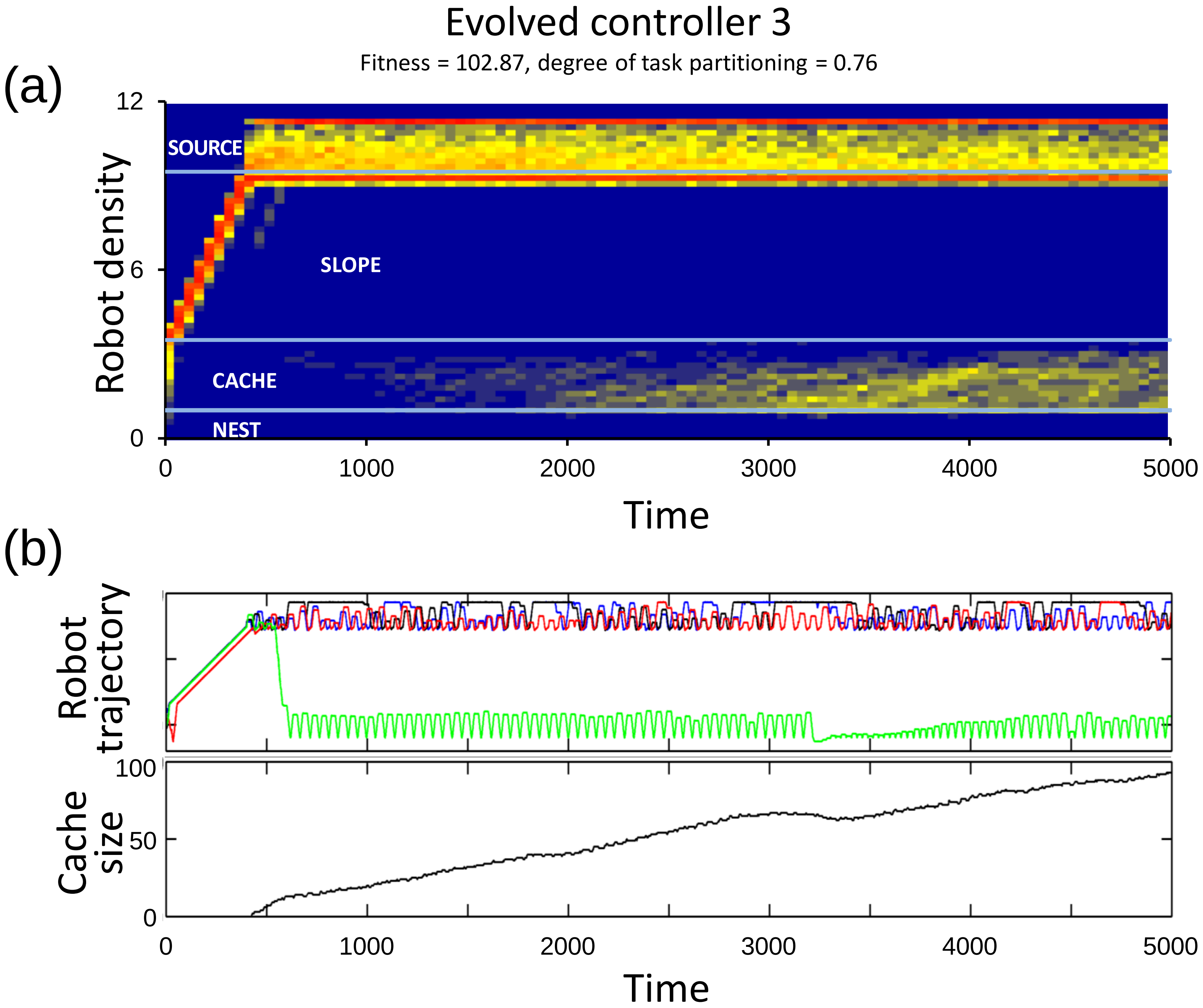

Evolved controller 3
Fitness = 102.87, degree of task partitioning = 0.76
(a)
Robot density
12
6
0
SOURCE
SLOPE
CACHE
NEST
0
1000
2000
3000
4000
5000
Time
(b)
Robot trajectory
Cache size
100
50
0
0
1000
2000
3000
4000
5000
Time

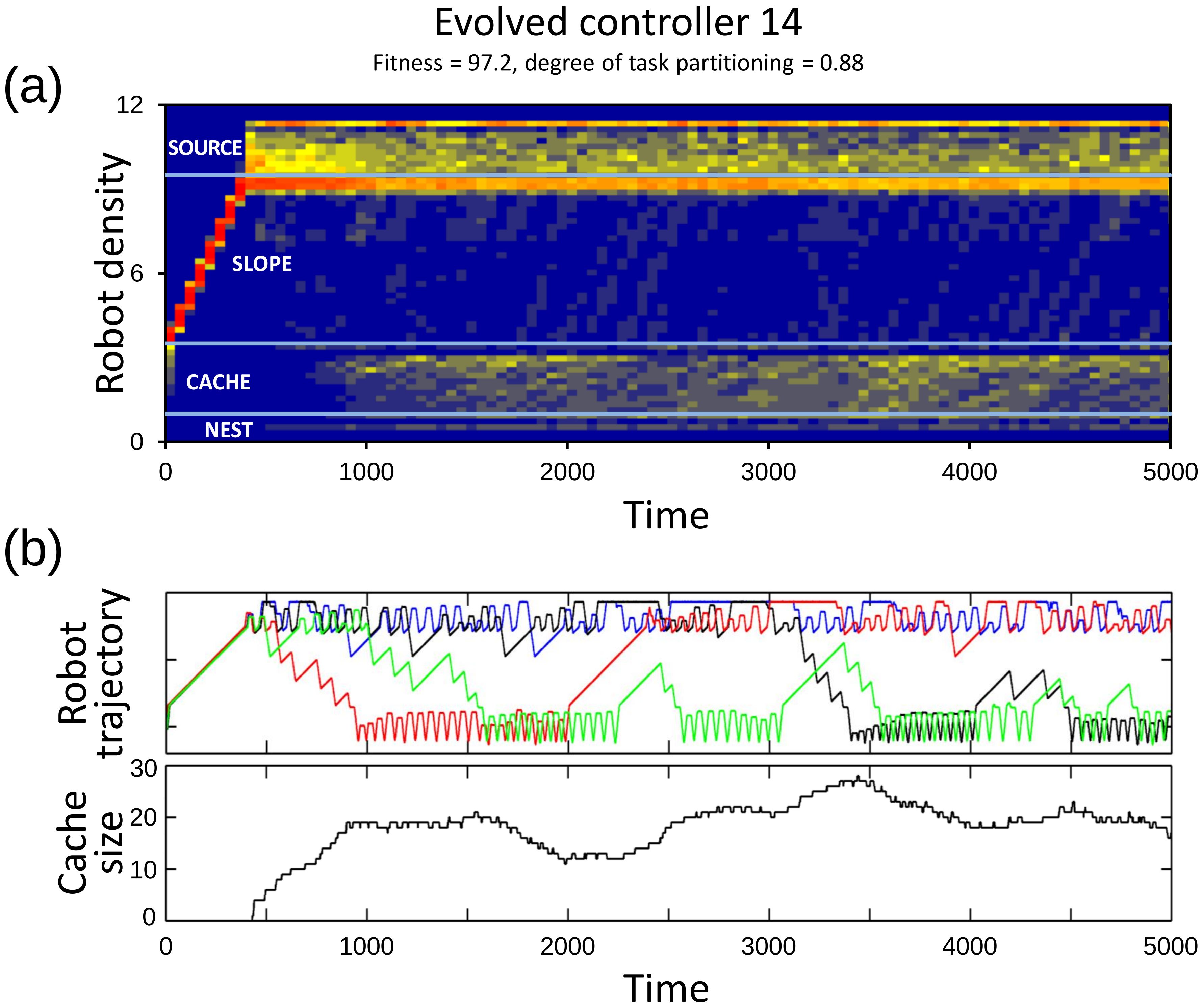

Evolved controller 14
Fitness = 97.2, degree of task partitioning = 0.88
(a)
Robot density
SOURCE
SLOPE
CACHE
NEST
12
6
0
0
1000
2000
3000
4000
5000
Time
(b)
Robot trajectory
Cache size
30
20
10
0
0
1000
2000
3000
4000
5000
Time

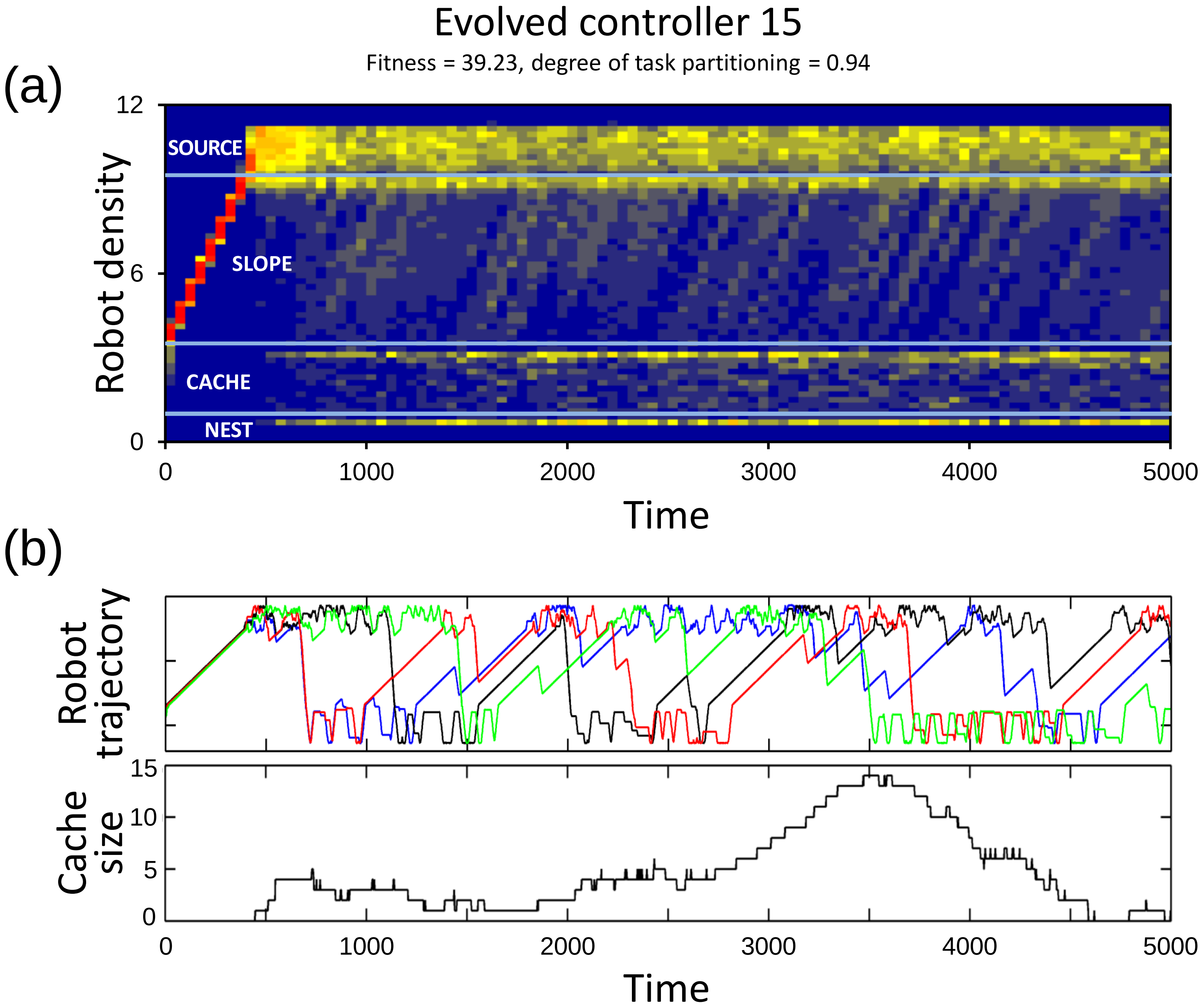

Evolved controller 15
Fitness = 39.23, degree of task partitioning = 0.94
(a)
Robot density
12
6
0
SOURCE
SLOPE
CACHE
NEST
0
1000
2000
3000
4000
5000
Time
(b)
Robot trajectory
Cache size
15
10
5
0
0
1000
2000
3000
4000
5000
Time

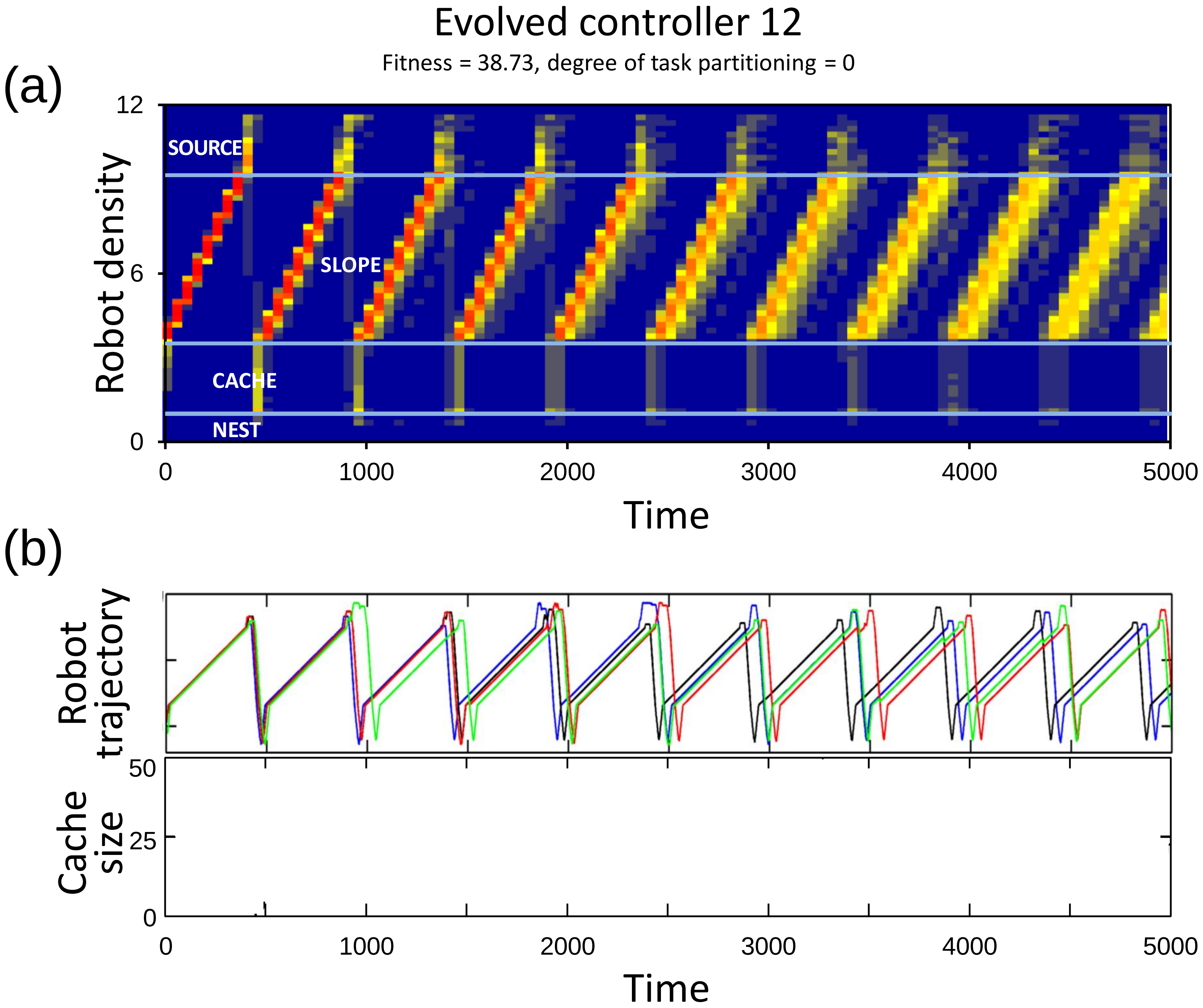
Evolved controller 12
Fitness = 38.73, degree of task partitioning = 0
(a)
Robot density
12
6
0
SOURCE
SLOPE
CACHE
NEST
0
1000
2000
3000
4000
5000
Time
(b)
Robot trajectory
Cache size
50
25
0
0
1000
2000
3000
4000
5000
Time

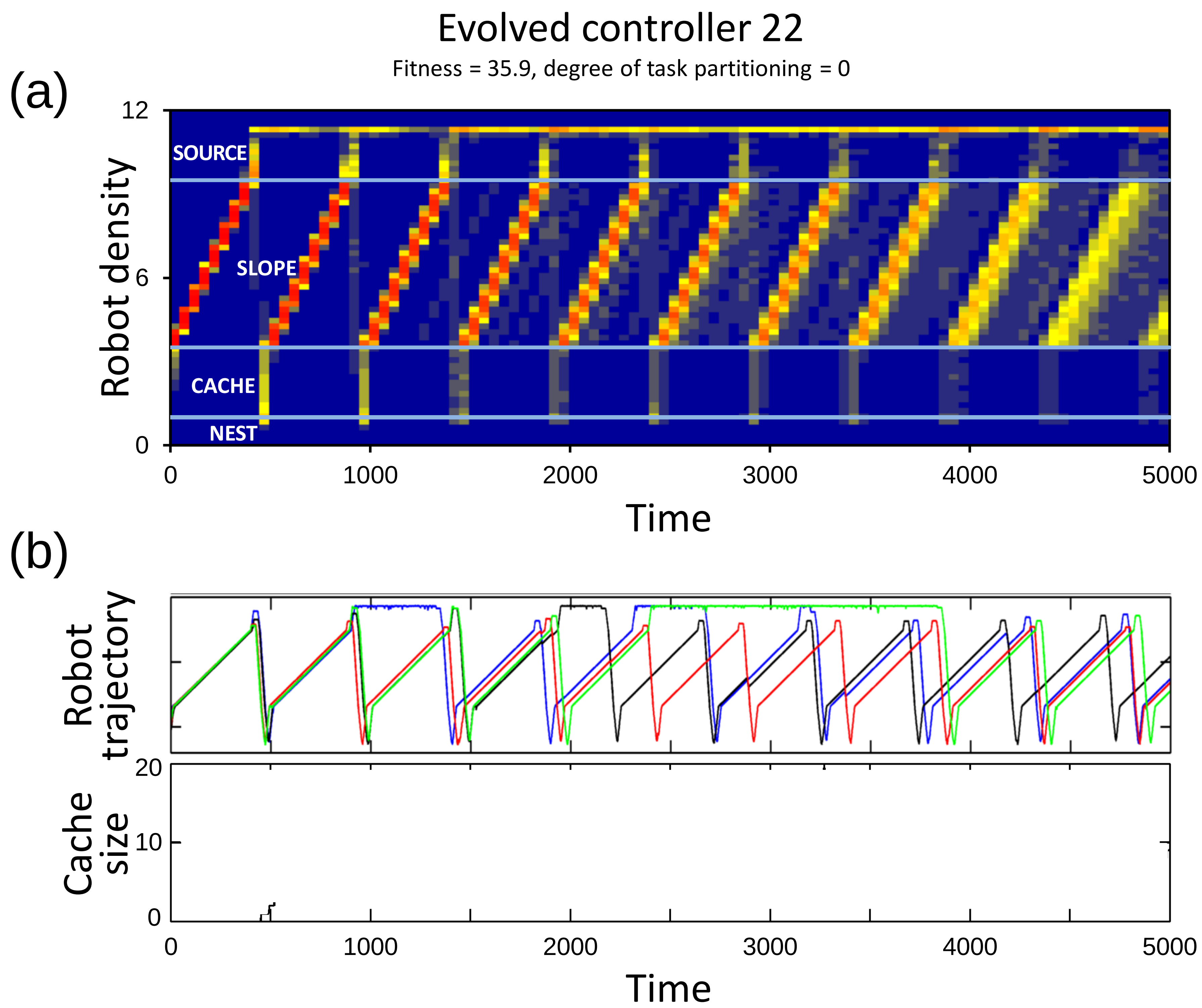

Evolved controller 22
Fitness = 35.9, degree of task partitioning = 0
(a)
Robot density
12
6
0
SOURCE
SLOPE
CACHE
NEST
0
1000
2000
3000
4000
5000
Time
(b)
Robot trajectory
Cache size
20
10
0
0
1000
2000
3000
4000
5000
Time

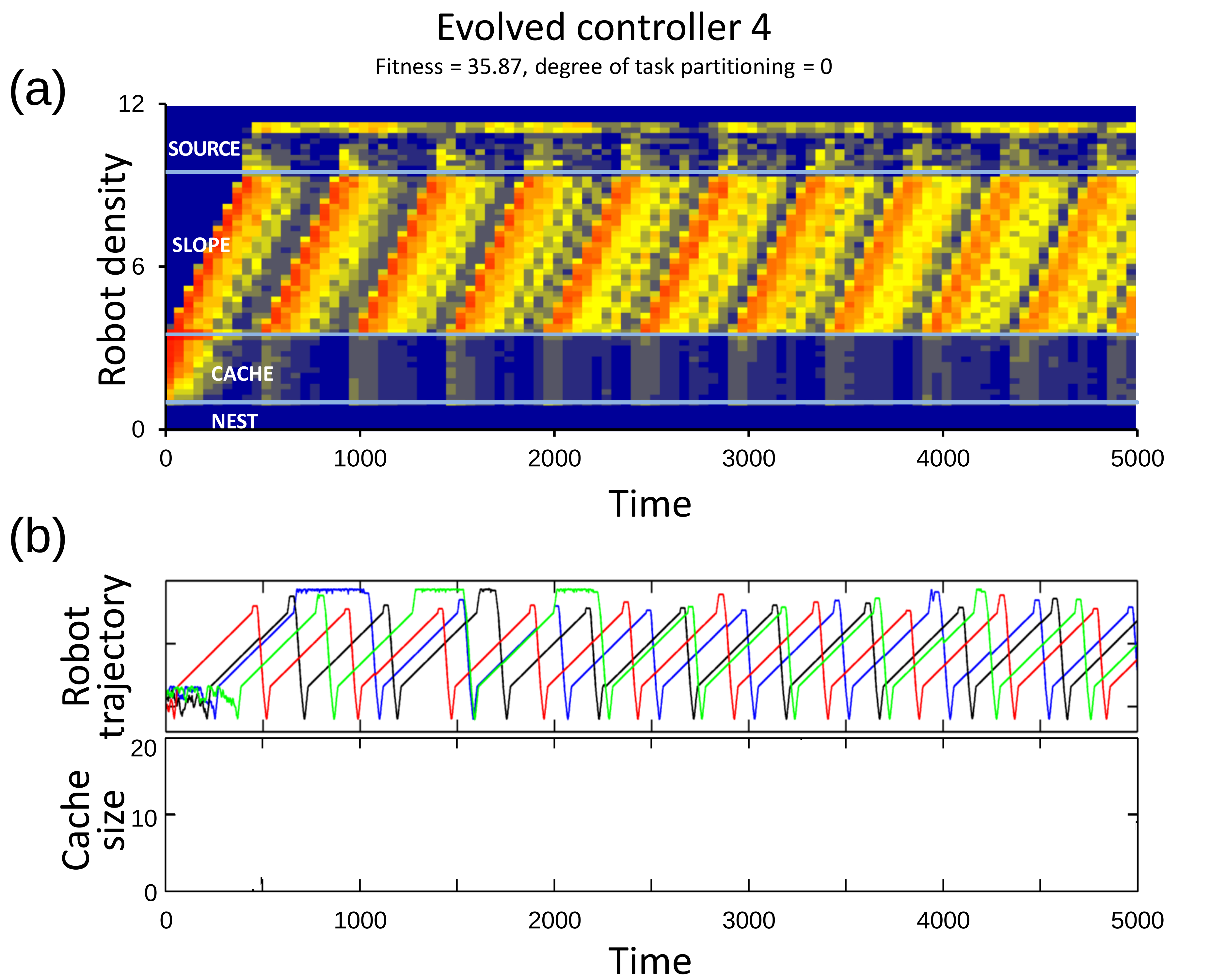
Evolved controller 4
Fitness = 35.87, degree of task partitioning = 0
(a)
Robot density
12
6
0
SOURCE
SLOPE
CACHE
NEST
0
1000
2000
3000
4000
5000
Time
(b)
Robot trajectory
Cache size
20
10
0
0
1000
2000
3000
4000
5000
Time

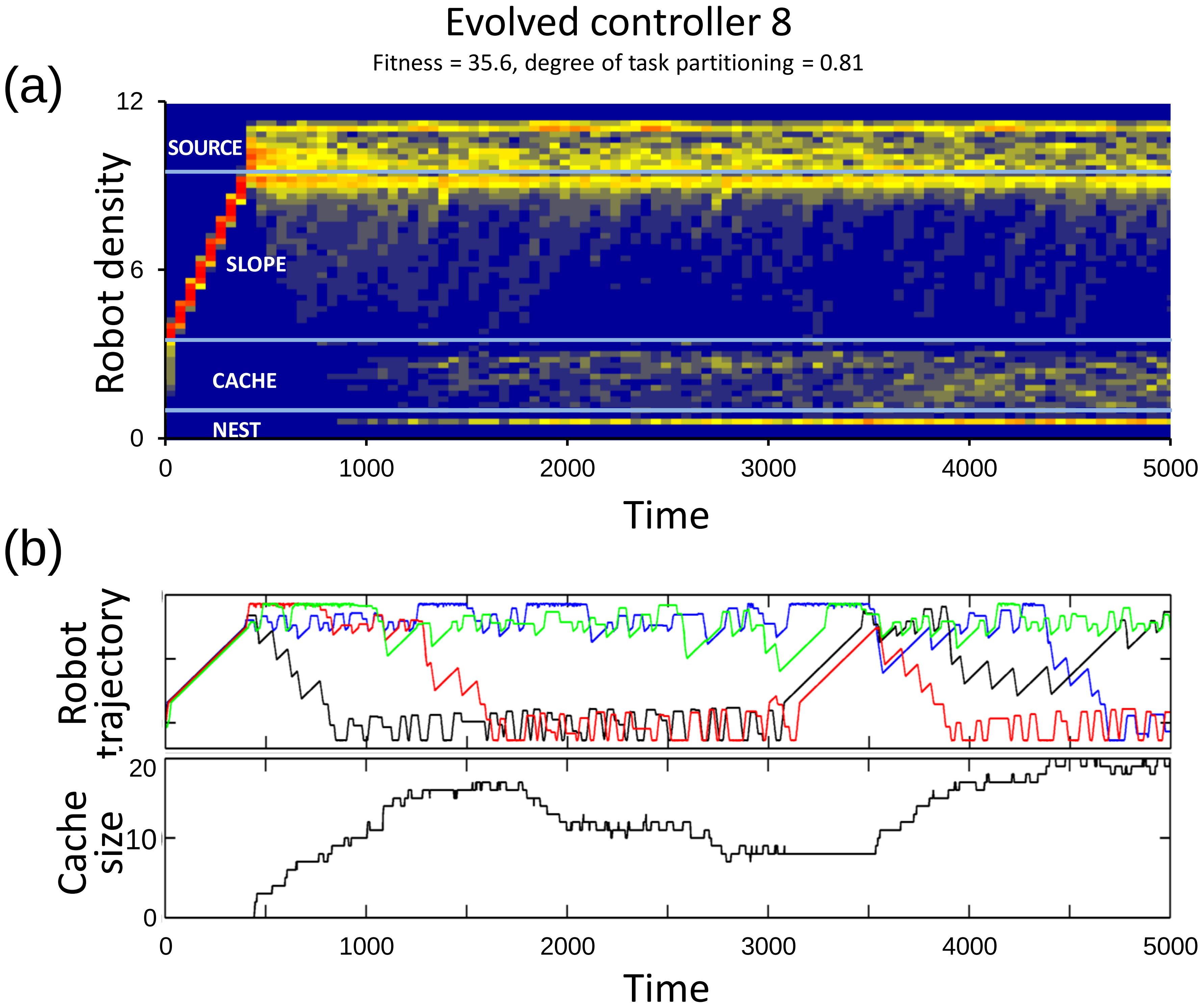

Evolved controller 8
Fitness = 35.6, degree of task partitioning = 0.81
(a)
Robot density
12
6
0
SOURCE
SLOPE
CACHE
NEST
0
1000
2000
3000
4000
5000
Time
(b)
Robot trajectory
Cache size
20
10
0
0
1000
2000
3000
4000
5000
Time

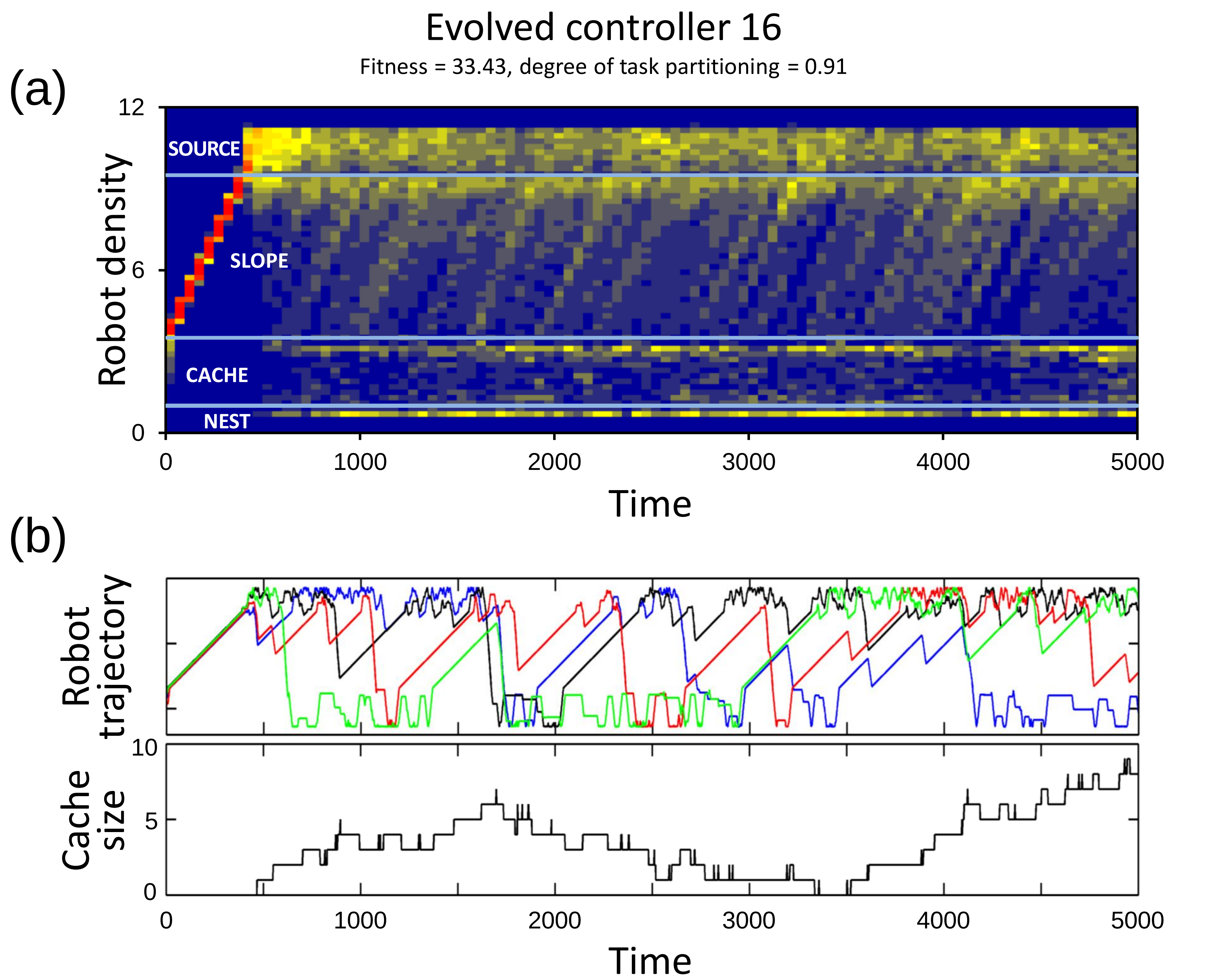

Evolved controller 16
Fitness = 33.43, degree of task partitioning = 0.91
(a)
Robot density
12
6
0
SOURCE
SLOPE
CACHE
NEST
0
1000
2000
3000
4000
5000
Time
(b)
Robot trajectory
Cache size
10
5
0
0
1000
2000
3000
4000
5000
Time

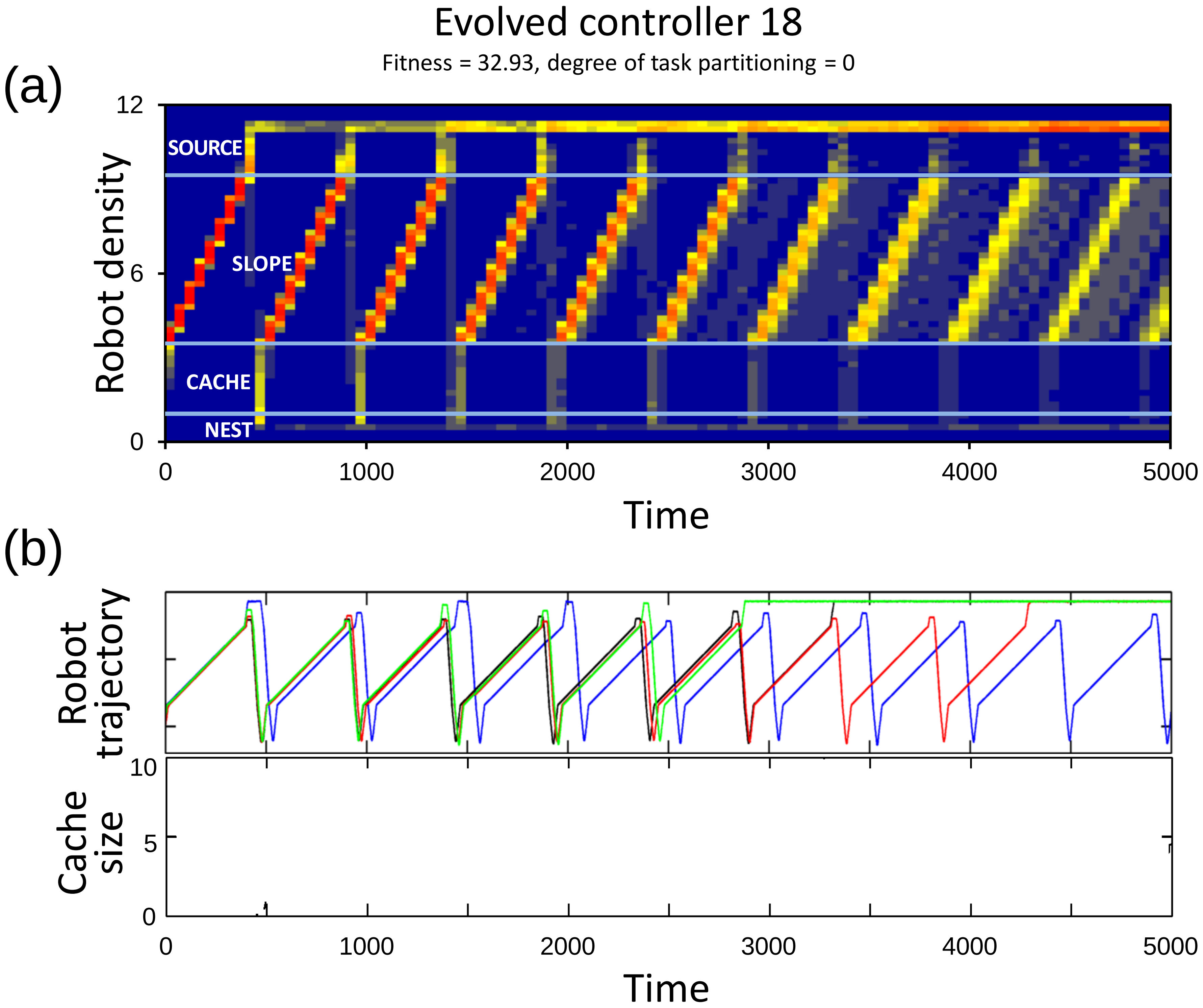

Evolved controller 18
Fitness = 32.93, degree of task partitioning = 0
(a)
Robot density
12
6
0
SOURCE
SLOPE
CACHE
NEST
0
1000
2000
3000
4000
5000
Time
(b)
Robot trajectory
Cache size
10
5
0
0
1000
2000
3000
4000
5000
Time

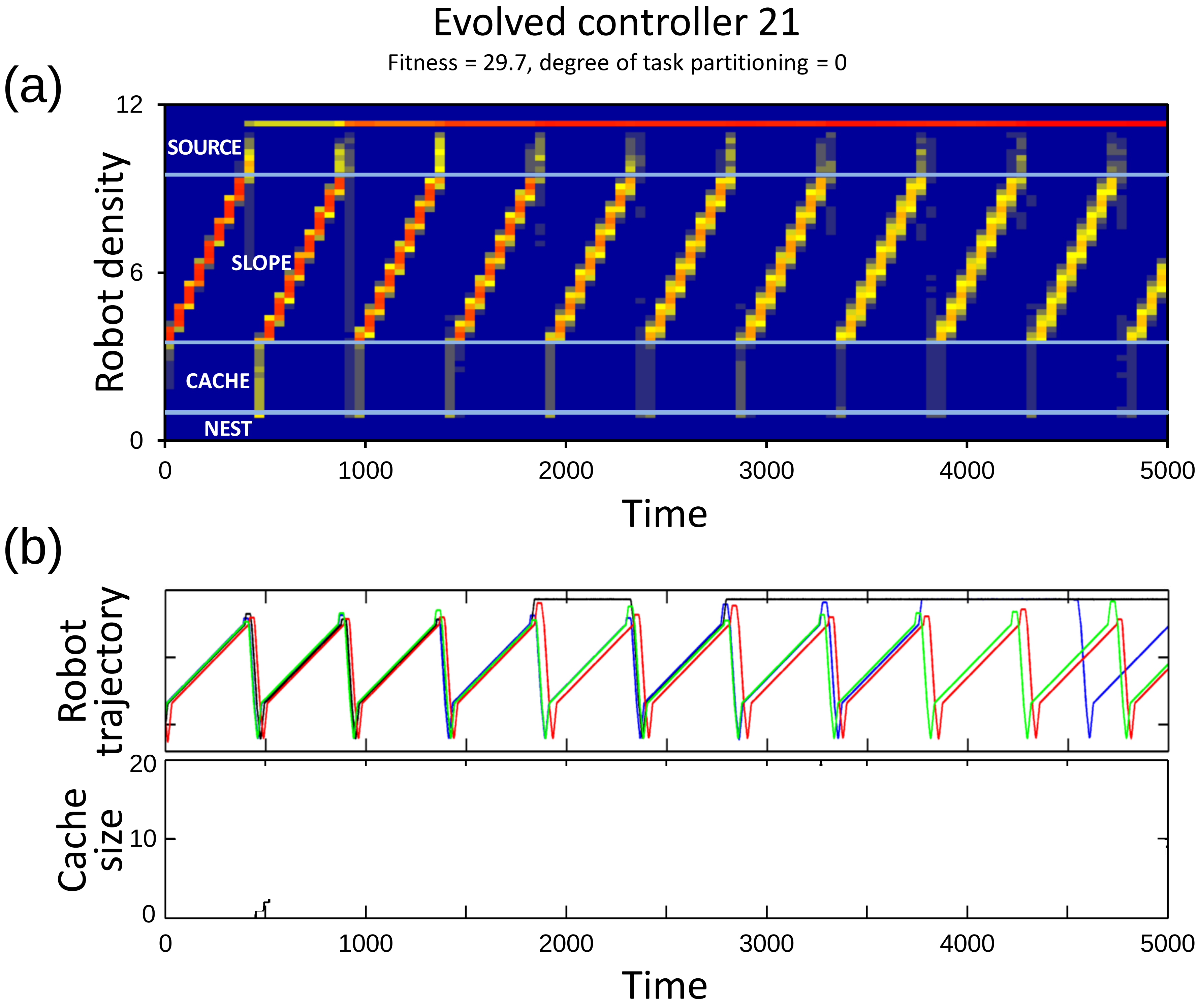

Evolved controller 21
Fitness = 29.7, degree of task partitioning = 0
(a)
Robot density
SOURCE
SLOPE
CACHE
NEST
12
6
0
0
1000
2000
3000
4000
5000
Time
(b)
Robot trajectory
Cache size
20
10
0
0
1000
2000
3000
4000
5000
Time

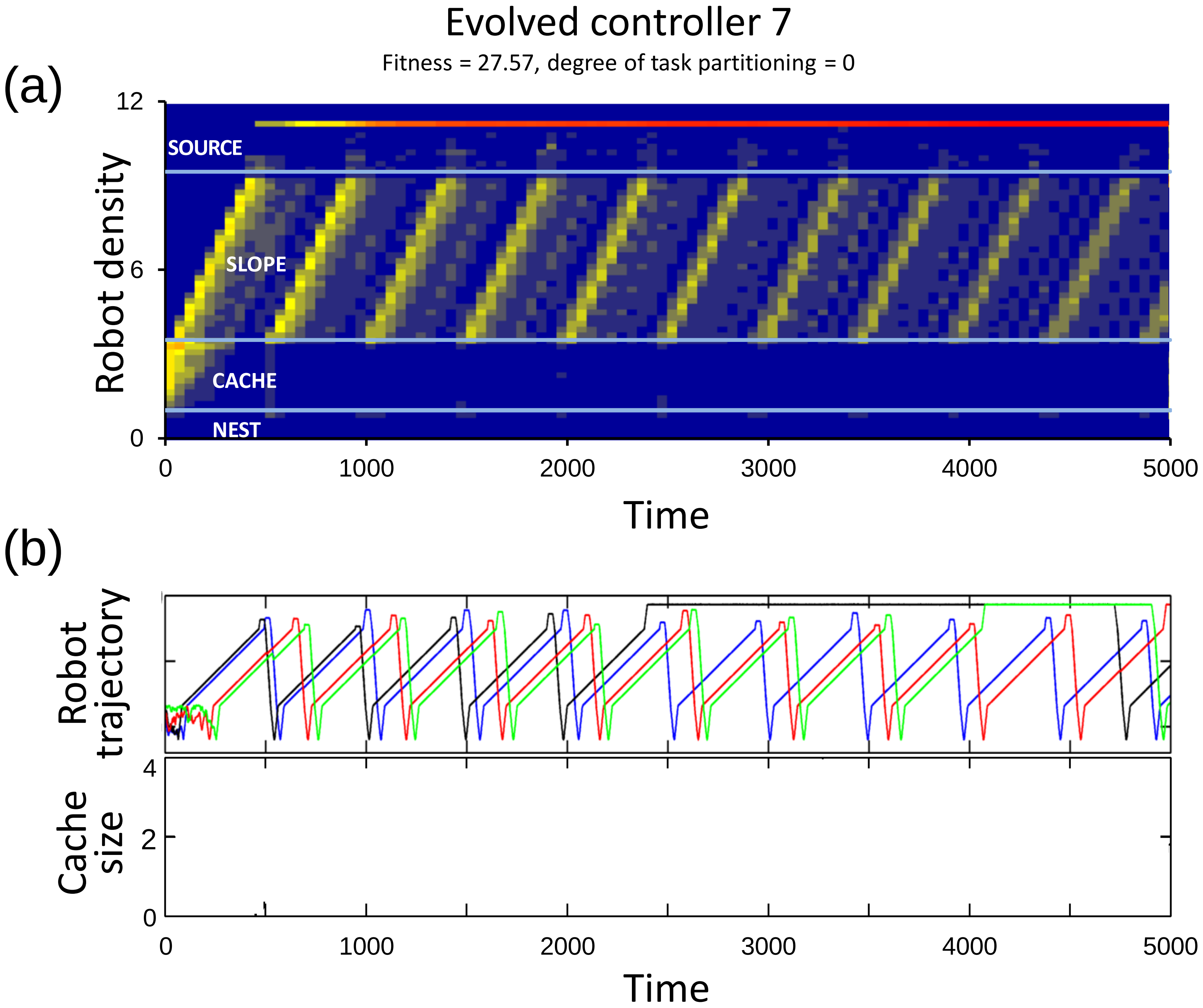

Evolved controller 7
Fitness = 27.57, degree of task partitioning = 0
(a)
12
6
0
Robot density
SOURCE
SLOPE
CACHE
NEST
0
1000
2000
3000
4000
5000
Time
(b)
Robot trajectory
4
2
0
Cache size
0
1000
2000
3000
4000
5000
Time

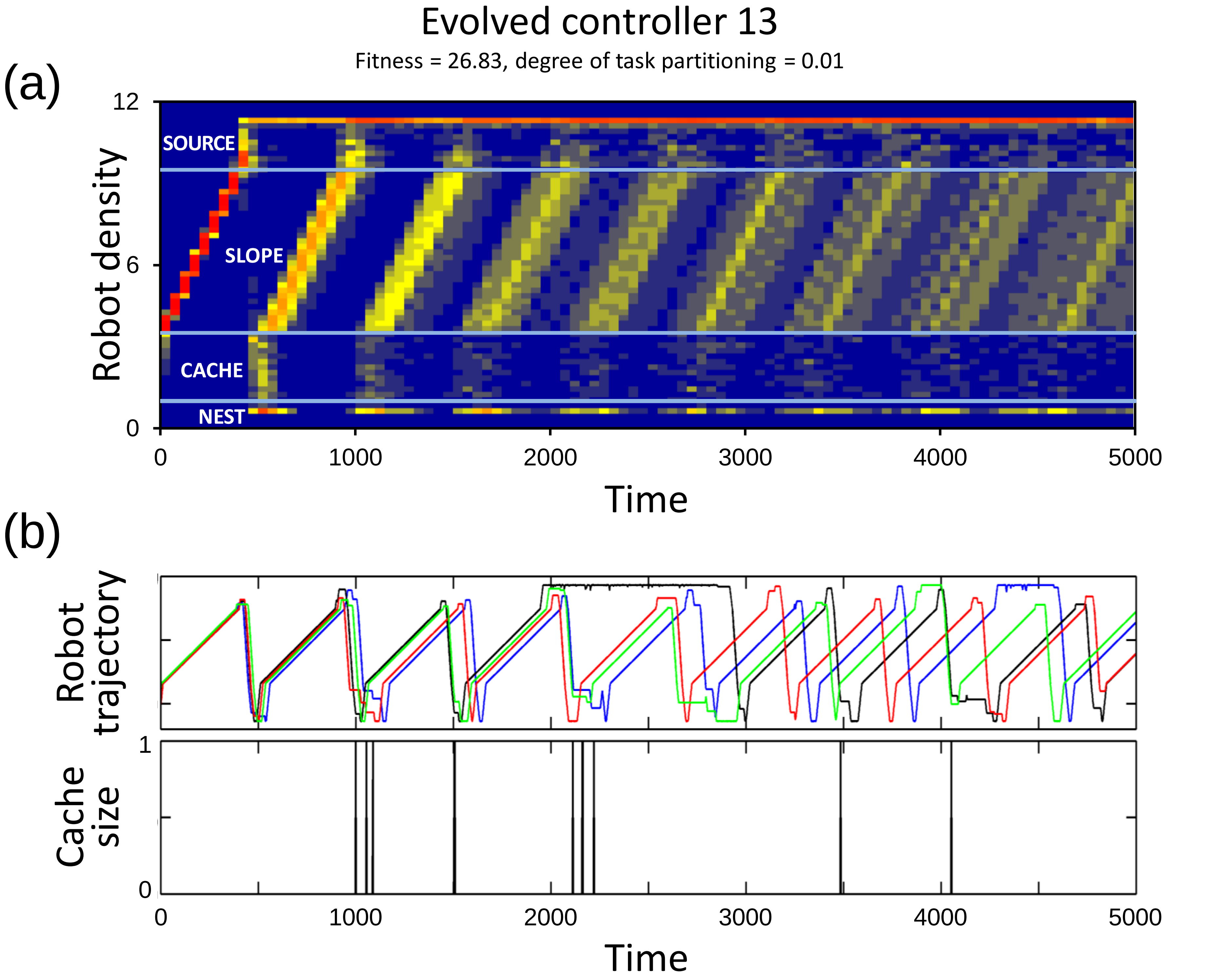

Evolved controller 13
Fitness = 26.83, degree of task partitioning = 0.01
(a)
Robot density
SOURCE
SLOPE
CACHE
NEST
12
6
0
0
1000
2000
3000
4000
5000
Time
(b)
Robot trajectory
Cache size
1
0
0
1000
2000
3000
4000
5000
Time

Evolved controller 6

Fitness = 22.87, degree of task partitioning = 0.06

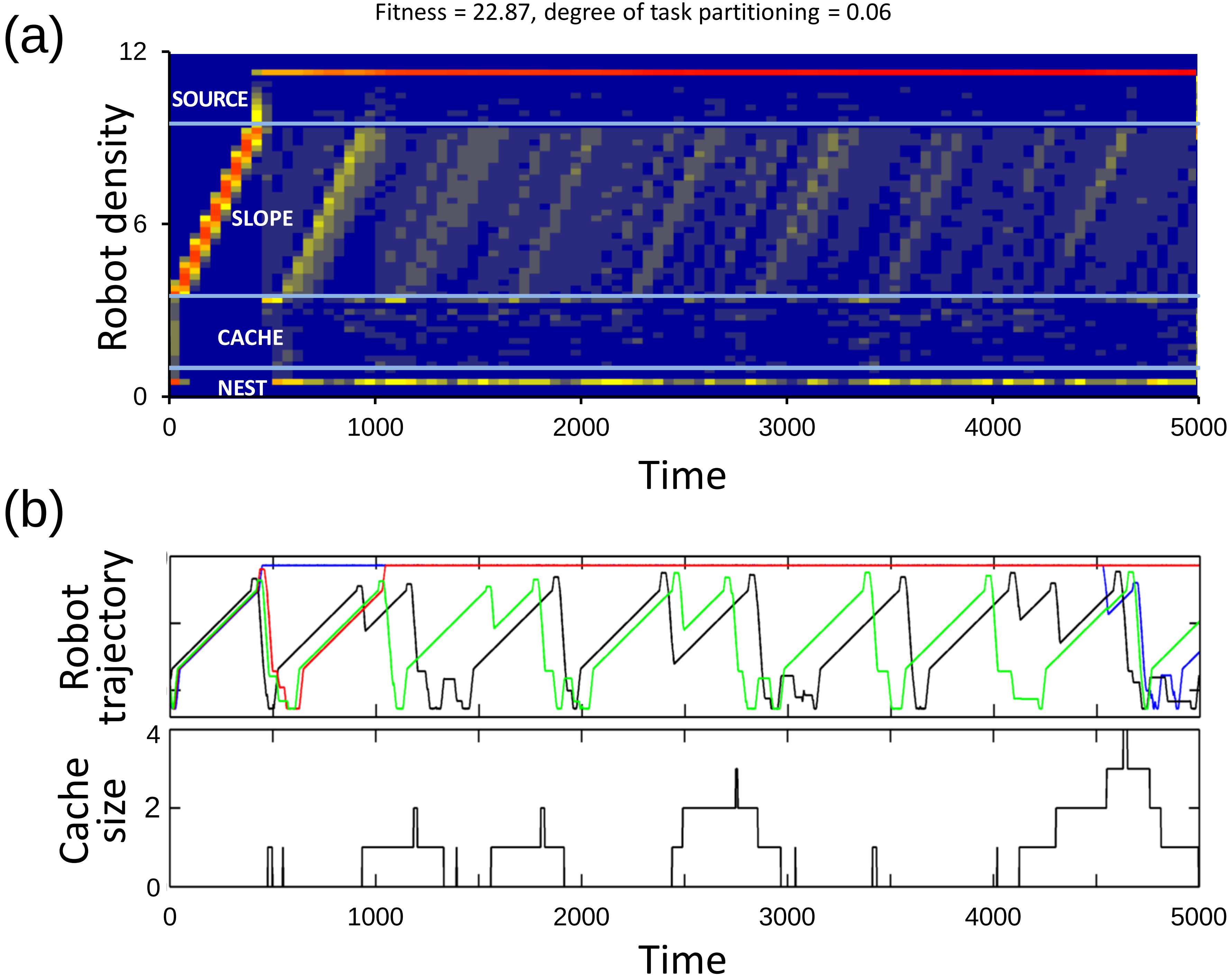

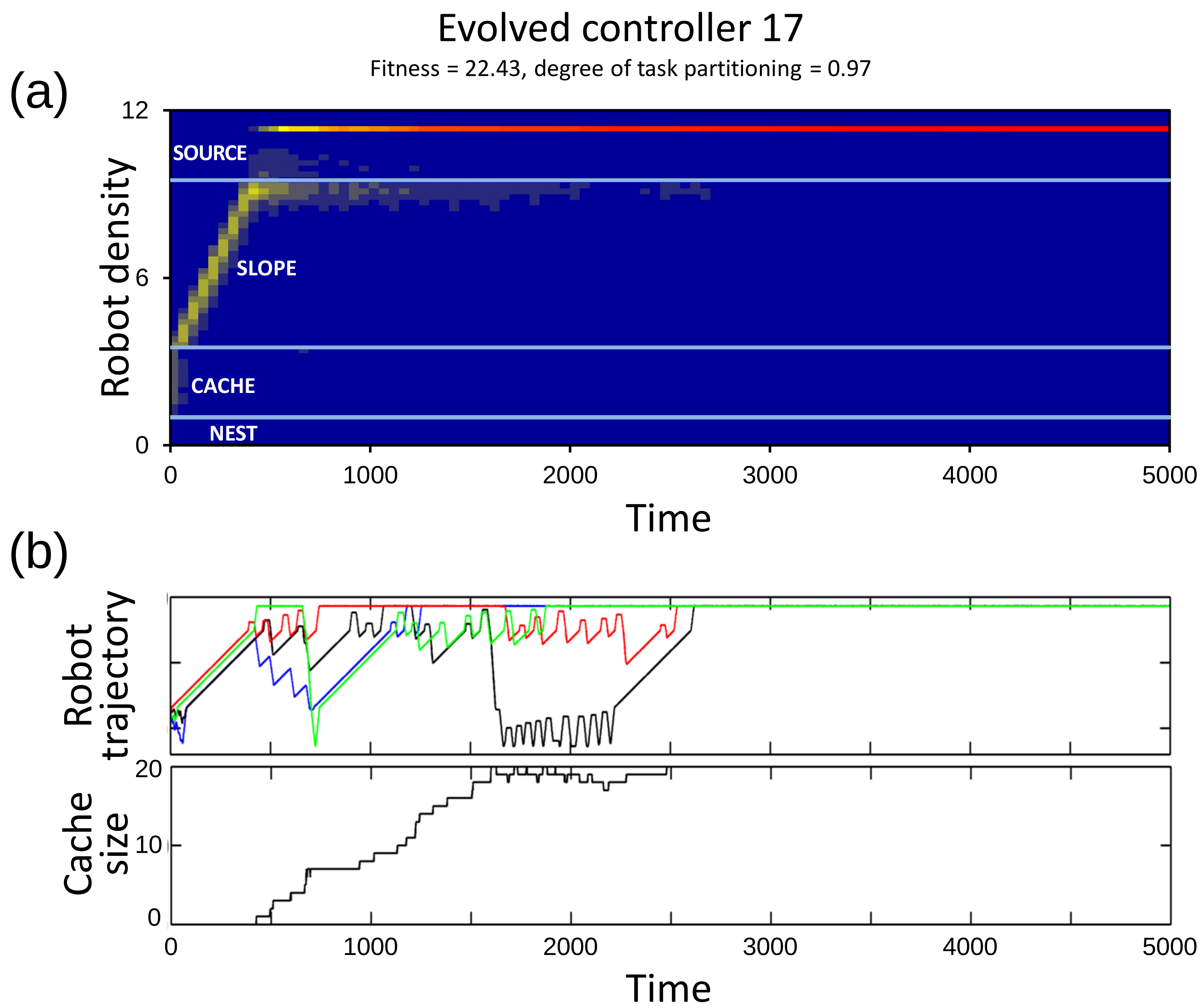
Evolved controller 17
Fitness = 22.43, degree of task partitioning = 0.97
(a)
Robot density
SOURCE
SLOPE
CACHE
NEST
12
6
0
0
1000
2000
3000
4000
5000
Time
(b)
Robot trajectory
Cache size
20
10
0
0
1000
2000
3000
4000
5000
Time

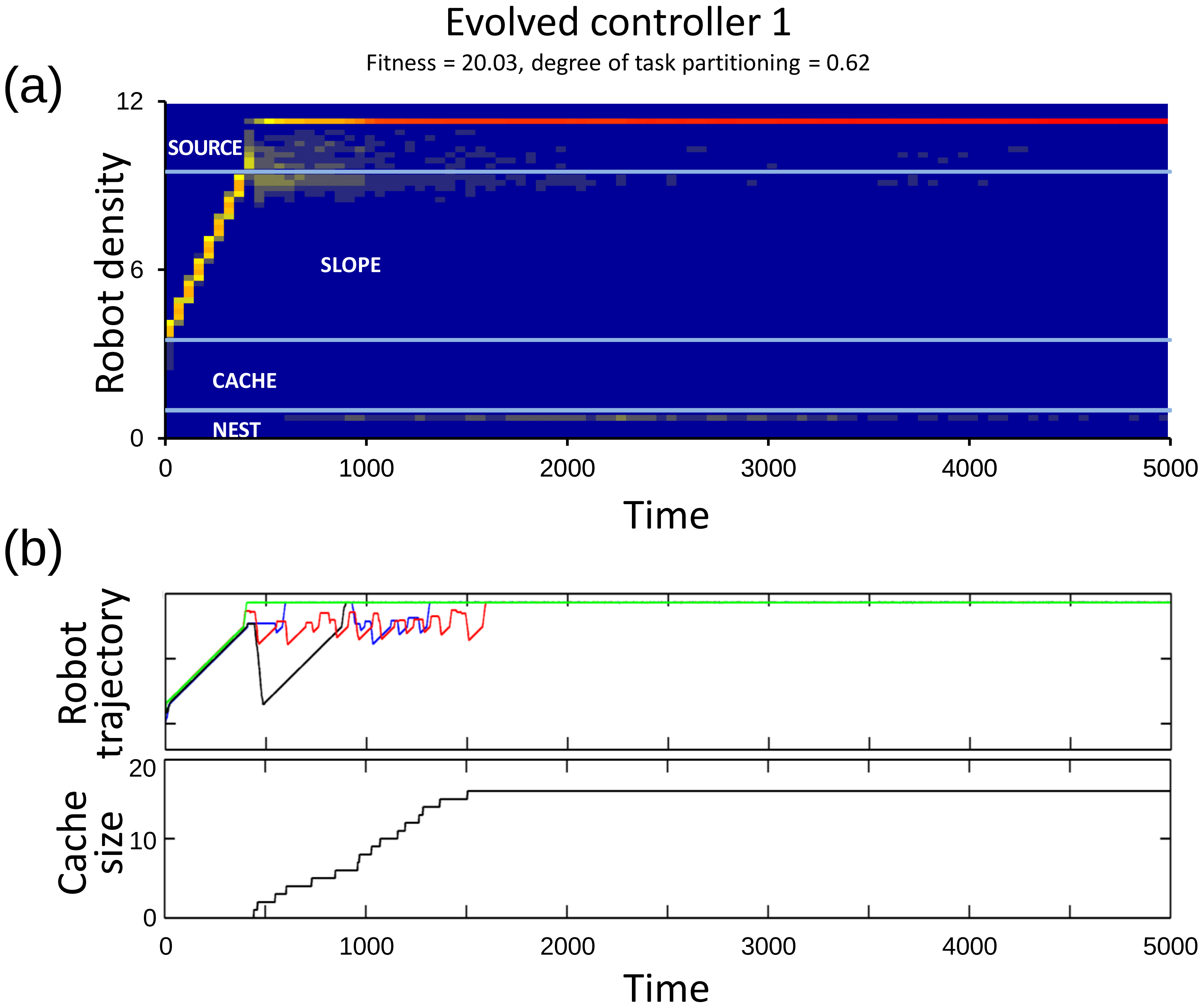

Evolved controller 1
Fitness = 20.03, degree of task partitioning = 0.62
(a)
Robot density
12
6
0
SOURCE
SLOPE
CACHE
NEST
0
1000
2000
3000
4000
5000
Time
(b)
Robot trajectory
Cache size
20
10
0
0
1000
2000
3000
4000
5000
Time

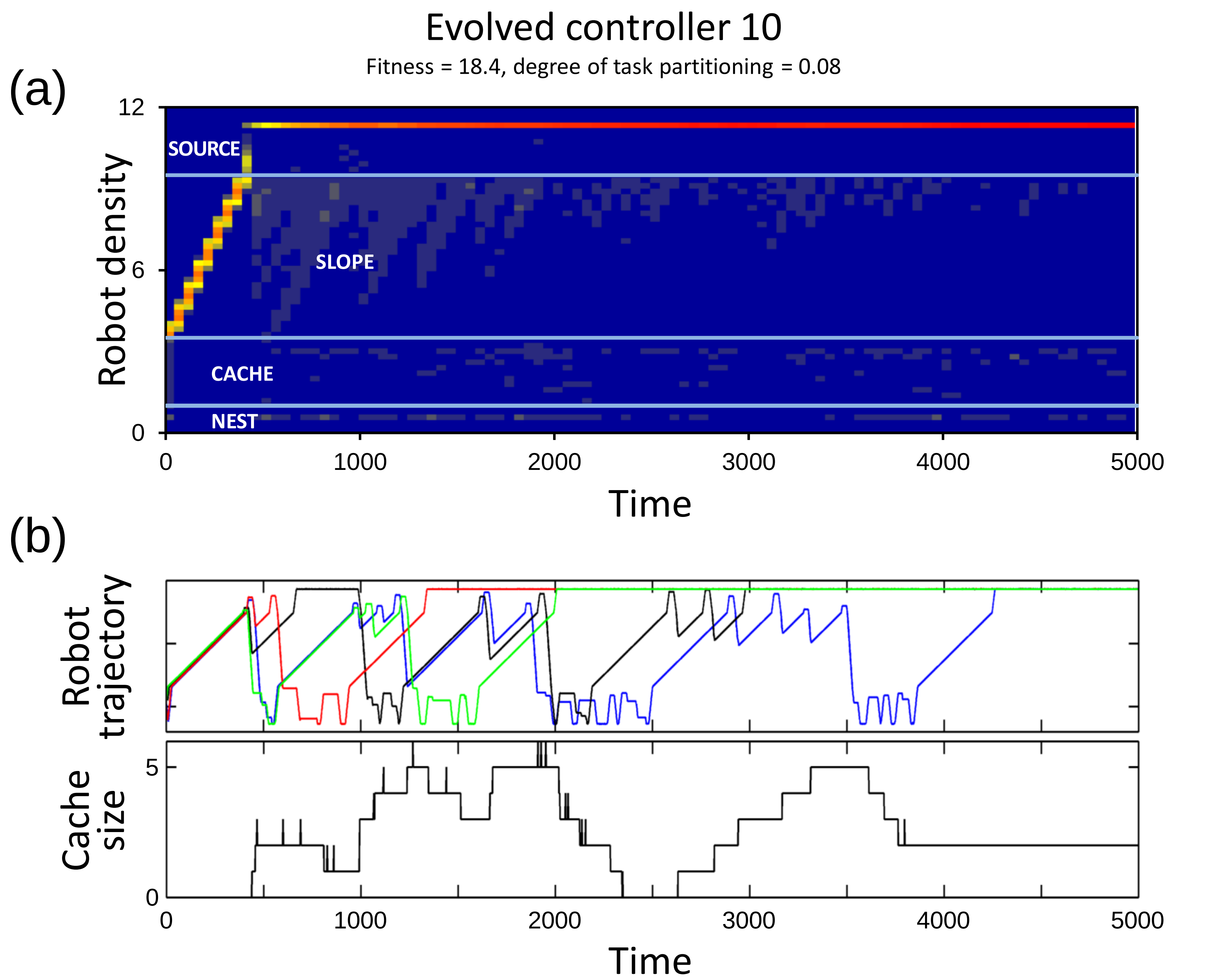

Evolved controller 10
Fitness = 18.4, degree of task partitioning = 0.08
(a)
Robot density
SOURCE
SLOPE
CACHE
NEST
12
6
0
0
1000
2000
3000
4000
5000
Time
(b)
Robot trajectory
Cache size
5
0
0
1000
2000
3000
4000
5000
Time
