# Supplementary material for: Evolution of Self-Organized Task Specialization in Robot Swarms
Source: PLoS Comput Biol. 2015 Aug 6;11(8):e1004273. doi: 10.1371/journal.pcbi.1004273 (PMC4527708; doi:10.1371/journal.pcbi.1004273)
Supplement: S1 Table — Most of the rules are used by more than one behavioral building block (rules R1 and R4-R6 are used by droppers, rules R2-R3, R5 and R7-R8 are used by collectors and rules R1, R4-R5 and R7-R8 are used by generalists). For each rule: the first row contains the list of preconditions, each denoted by the syntax P NAME = True|False where NAME is the intuitive name of the precondition; the second row contains the list of fine-grained behavioral building blocks (B RANDOM_WALK, B PHOTOTAXIS, B ANTI-PHOTOTAXIS, c.f. Materials and Methods); the remaining rows contain the list of actions (one per row), where the first column indicates the type of the action (A B are actions that change the currently-executed behavior, while A IS are all other actions), the second column indicates the execution probability, and the third column indicates the effect of the action (either the new behavior to switch to in case of A B or the new value of the internal state IS NAME in case of A IS). Memory states were set as follows: P STAY_DOWN = True and P STAY_UP = False for collectors, P STAY_DOWN = False and P STAY_UP = True for droppers and P STAY_DOWN = False and P STAY_UP = False for generalists. (PDF) [file pcbi.1004273.s002.pdf]

Table S1. Rules used to encode the dropper, collector and generalist foraging strategies in the experiments with pre-adapted building blocks.

R_1:	Generalist and Dropper		
$\mathcal{P}_1$	$P_{STAY\_DOWN} == False$	$P_{HAS\_FOOD} == False$	$P_{ON\_SOURCE} == False$
$\mathcal{B}_1$	B_{RANDOM_WALK}	$B_{GO\_TO\_NEST}$	$B_{GO\_TO\_SOURCE}$
$\mathcal{A}_1$	A_B	$p = 1.0$	$B_{GO\_TO\_SOURCE}$
R_2:	Collector		
$\mathcal{P}_2$	$P_{ON\_SLOPE} == True$	$P_{STAY\_DOWN} == True$	
$\mathcal{B}_2$	B_{RANDOM_WALK}	$B_{GO\_TO\_NEST}$	$B_{GO\_TO\_SOURCE}$
$\mathcal{A}_2$	A_B	$p = 1.0$	$B_{GO\_TO\_NEST}$
R_3:	Collector		
$\mathcal{P}_3$	$P_{STAY\_DOWN} == True$	$P_{HAS\_FOOD} == False$	$P_{ON\_GRASS} == True$
$\mathcal{B}_3$	$B_{GO\_TO\_NEST}$		
$\mathcal{A}_3$	A_B<br>A_{IS}	$p = 0.01$<br>$p = 1.0$	B_{RANDOM_WALK}<br>$IS_{WANT\_FOOD} \leftarrow True$
R_4:	Generalist or Dropper		
$\mathcal{P}_4$	$P_{ON\_SOURCE} == True$	$P_{HAS\_FOOD} == False$	
$\mathcal{B}_4$	$B_{GO\_TO\_SOURCE}$		
$\mathcal{A}_4$	A_B<br>A_{IS}	$p = 0.01$<br>$p = 1.0$	B_{RANDOM_WALK}<br>$IS_{WANT\_FOOD} \leftarrow True$
R_5:	Generalist, Dropper or Collector		
$\mathcal{P}_5$	$P_{HAS\_FOOD} == True$		
$\mathcal{B}_5$	B_{RANDOM_WALK}	$B_{GO\_TO\_SOURCE}$	
$\mathcal{A}_5$	A_B<br>A_{IS}	$p = 1.0$<br>$p = 1.0$	$B_{GO\_TO\_NEST}$<br>$IS_{WANT\_FOOD} \leftarrow False$
R_6:	Dropper		
$\mathcal{P}_6$	$P_{ON\_SLOPE} == True$	$P_{HAS\_FOOD} == True$	$P_{STAY\_UP} == True$
$\mathcal{B}_6$	B_{RANDOM_WALK}	$B_{GO\_TO\_NEST}$	$B_{GO\_TO\_SOURCE}$
$\mathcal{A}_6$	A_B<br>A_{IS}	$p = 1.0$<br>$p = 1.0$	$B_{GO\_TO\_SOURCE}$<br>$IS_{DROP\_FOOD} \leftarrow True$
R_7:	Generalist or Collector		
$\mathcal{P}_7$	$P_{HAS\_FOOD} == True$	$P_{ON\_NEST} == True$	
$\mathcal{B}_7$	B_{RANDOM_WALK}	$B_{GO\_TO\_NEST}$	
$\mathcal{A}_7$	A_B<br>A_{IS}<br>A_{IS}	$p = 1.0$<br>$p = 1.0$<br>$p = 1.0$	B_{RANDOM_WALK}<br>$IS_{DROP\_FOOD} \leftarrow True$<br>$IS_{WANT\_FOOD} \leftarrow True$
R_8:	Generalist or Collector		
$\mathcal{P}_8$	$P_{ON\_NEST} == True$	$P_{HAS\_FOOD} == False$	
$\mathcal{B}_8$	B_{RANDOM_WALK}	$B_{GO\_TO\_NEST}$	
$\mathcal{A}_8$	A_B	$p = 1.0$	$B_{GO\_TO\_SOURCE}$
