# Supplementary material for: Evolution of Self-Organized Task Specialization in Robot Swarms
Source: PLoS Comput Biol. 2015 Aug 6;11(8):e1004273. doi: 10.1371/journal.pcbi.1004273 (PMC4527708; doi:10.1371/journal.pcbi.1004273)
Supplement: S2 Table — Controllers are sorted from high to low group performance. (PDF) [file pcbi.1004273.s003.pdf]

Table S2. Rules evolved via Grammatical Evolution in the 22 evolutionary runs.

R_1:			
$\mathcal{P}_1$	$P_{ON\_NEST} == True$		
$\mathcal{B}_1$	B_{RANDOM_WALK}	$B_{GO\_TO\_NEST}$	
$\mathcal{A}_1$	A_B<br>A_B<br>A_{IS}<br>A_{IS}	$p = 0.05$<br>$p = 0.025$<br>$p = 0.05$<br>$p = 0.025$	$B_{GO\_TO\_SOURCE}$<br>B_{RANDOM_WALK}<br>$IS_{DROP\_FOOD} \leftarrow True$<br>$IS_{MOTIVATION_2} -$
R_2:			
$\mathcal{P}_2$	$P_{ON\_NEST} == False$		
$\mathcal{B}_2$	B_{RANDOM_WALK}	$B_{GO\_TO\_SOURCE}$	
$\mathcal{A}_2$	A_B	$p = 0.025$	B_{RANDOM_WALK}
R_3:			
$\mathcal{P}_3$	$P_{HAS\_FOOD} == True$		
$\mathcal{B}_3$	B_{RANDOM_WALK}	$B_{GO\_TO\_NEST}$	$B_{GO\_TO\_SOURCE}$
$\mathcal{A}_3$	A_B	$p = 0.01$	$B_{GO\_TO\_SOURCE}$
R_4:			
$\mathcal{P}_4$	$P_{HAS\_FOOD} == False$		
$\mathcal{B}_4$	B_{RANDOM_WALK}	$B_{GO\_TO\_SOURCE}$	
$\mathcal{A}_4$	A_{IS}<br>A_B<br>A_{IS}	$p = 0.005$<br>$p = 0.025$<br>$p = 1.0$	$IS_{WANT\_FOOD} \leftarrow False$<br>$B_{GO\_TO\_SOURCE}$<br>$IS_{WANT\_FOOD} \leftarrow True$
R_5:			
$\mathcal{P}_5$	$P_{HAS\_FOOD} == True$	$P_{ON\_NEST} == False$	$P_{MOTIVATION_2} <= 0.5$
$\mathcal{B}_5$	B_{RANDOM_WALK}	$B_{GO\_TO\_SOURCE}$	
$\mathcal{A}_5$	A_B<br>A_B<br>A_{IS}	$p = 1.0$<br>$p = 1.0$<br>$p = 0.005$	B_{RANDOM_WALK}<br>$B_{GO\_TO\_NEST}$<br>$IS_{MOTIVATION_1} +$
R_6:			
$\mathcal{P}_6$	$P_{ON\_SLOPE} == True$	$P_{ON\_NEST} == False$	$P_{MOTIVATION_2} <= 0.5$
$\mathcal{B}_6$	$B_{GO\_TO\_NEST}$	$B_{GO\_TO\_SOURCE}$	
$\mathcal{A}_6$	A_B<br>A_{IS}	$p = 1.0$<br>$p = 0.05$	$B_{GO\_TO\_SOURCE}$<br>$IS_{DROP\_FOOD} \leftarrow True$
R_7:			
$\mathcal{P}_7$	$P_{ON\_SOURCE} == False$		
$\mathcal{B}_7$	B_{RANDOM_WALK}		
$\mathcal{A}_7$	A_{IS}	$p = 0.1$	$IS_{MOTIVATION_2} -$
R_8:			
$\mathcal{P}_8$	$P_{ON\_SLOPE} == False$	$P_{ON\_SLOPE} == True$	$P_{MOTIVATION_2} > 0.5$
$\mathcal{B}_8$	$B_{GO\_TO\_NEST}$	$B_{GO\_TO\_SOURCE}$	
$\mathcal{A}_8$	A_{IS}	$p = 0.001$	$IS_{DROP\_FOOD} \leftarrow True$

Evolved controller 20

R_1:			
$\mathcal{P}_1$	$P_{ON\_GRASS} == False$	$P_{ON\_SOURCE} == False$	
$\mathcal{B}_1$	$B_{GO\_TO\_NEST}$		
$\mathcal{A}_1$	A_{IS}<br>A_{IS}<br>A_B	$p = 0.075$<br>$p = 0.025$<br>$p = 0.075$	$IS_{MOTIVATION_2}$ +<br>$IS_{MOTIVATION_1}$ −<br>$B_{GO\_TO\_SOURCE}$
R_2:			
$\mathcal{P}_2$	$P_{HAS\_FOOD} == True$	$P_{MOTIVATION_2} > 0.5$	
$\mathcal{B}_2$	B_{RANDOM_WALK}	$B_{GO\_TO\_NEST}$	$B_{GO\_TO\_SOURCE}$
$\mathcal{A}_2$	A_{IS}<br>A_B<br>A_{IS}	$p = 0.075$<br>$p = 0.05095$<br>$p = 0.1$	$IS_{MOTIVATION_1}$ +<br>$B_{GO\_TO\_NEST}$<br>$IS_{DROP\_FOOD} \leftarrow False$
R_3:			
$\mathcal{P}_3$	$P_{ON\_SLOPE} == False$		
$\mathcal{B}_3$	$B_{GO\_TO\_SOURCE}$		
$\mathcal{A}_3$	A_{IS}<br>A_B	$p = 1.0$<br>$p = 0.005$	$IS_{MOTIVATION_1}$ +<br>B_{RANDOM_WALK}
R_4:			
$\mathcal{P}_4$	$P_{ON\_GRASS} == False$	$P_{MOTIVATION_2} <= 0.5$	
$\mathcal{B}_4$	$B_{GO\_TO\_SOURCE}$		
$\mathcal{A}_4$	A_{IS}	$p = 0.01$	$IS_{MOTIVATION_2}$ +
R_5:			
$\mathcal{P}_5$	$P_{MOTIVATION_1} > 0.5$	$P_{ON\_GRASS} == False$	$P_{ON\_SOURCE} == False$
$\mathcal{B}_5$	B_{RANDOM_WALK}	$B_{GO\_TO\_NEST}$	
$\mathcal{A}_5$	A_B<br>A_{IS}<br>A_B<br>A_{IS}	$p = 0.1$<br>$p = 1.0$<br>$p = 0.005$<br>$p = 1.0$	$B_{GO\_TO\_SOURCE}$<br>$IS_{DROP\_FOOD} \leftarrow True$<br>$B_{GO\_TO\_NEST}$<br>$IS_{MOTIVATION_2}$ +
R_6:			
$\mathcal{P}_6$	$P_{ON\_GRASS} == False$	$P_{MOTIVATION_2} > 0.5$	
$\mathcal{B}_6$	B_{RANDOM_WALK}		
$\mathcal{A}_6$	A_B	$p = 0.01$	$B_{GO\_TO\_SOURCE}$

Evolved controller 9

R_1:			
$\mathcal{P}_1$	$P_{HAS\_FOOD} == False$		
$\mathcal{B}_1$	$B_{GO\_TO\_NEST}$		
$\mathcal{A}_1$	A_B<br>A_{IS}	$p = 1.0$<br>$p = 0.025$	$B_{GO\_TO\_SOURCE}$<br>$IS_{MOTIVATION_2}$ $-$
R_2:			
$\mathcal{P}_2$	$P_{ON\_GRASS} == False$	$P_{ON\_NEST} == True$	$P_{ON\_SOURCE} == False$
$\mathcal{B}_2$	$B_{GO\_TO\_NEST}$	$B_{GO\_TO\_SOURCE}$	
$\mathcal{A}_2$	A_B	$p = 0.075$	$B_{GO\_TO\_SOURCE}$
R_3:			
$\mathcal{P}_3$	$P_{ON\_NEST} == True$		
$\mathcal{B}_3$	$B_{GO\_TO\_NEST}$		
$\mathcal{A}_3$	A_B<br>A_{IS}	$p = 0.001$<br>$p = 0.1$	$B_{GO\_TO\_NEST}$<br>$IS_{DROP\_FOOD} \leftarrow True$
R_4:			
$\mathcal{P}_4$	$P_{HAS\_FOOD} == True$		
$\mathcal{B}_4$	B_{RANDOM_WALK}	$B_{GO\_TO\_SOURCE}$	
$\mathcal{A}_4$	A_{IS}<br>A_B<br>A_B<br>A_{IS}	$p = 0.005$<br>$p = 0.075$<br>$p = 0.001$<br>$p = 0.075$	$IS_{DROP\_FOOD} \leftarrow False$<br>$B_{GO\_TO\_NEST}$<br>$B_{RANDOM\_WALK}$<br>$IS_{DROP\_FOOD} \leftarrow True$
R_5:			
$\mathcal{P}_5$	$P_{ON\_GRASS} == False$		
$\mathcal{B}_5$	B_{RANDOM_WALK}		
$\mathcal{A}_5$	A_{IS}<br>A_{IS}	$p = 1.0$<br>$p = 0.005$	$IS_{MOTIVATION_1}$ $-$<br>$IS_{MOTIVATION_2}$ $-$
R_6:			
$\mathcal{P}_6$	$P_{ON\_NEST} == False$		
$\mathcal{B}_6$	B_{RANDOM_WALK}	$B_{GO\_TO\_SOURCE}$	
$\mathcal{A}_6$	A_{IS}<br>A_{IS}<br>A_B	$p = 0.001$<br>$p = 0.01$<br>$p = 0.075$	$IS_{MOTIVATION_1}$ $+$<br>$IS_{DROP\_FOOD} \leftarrow False$<br>$B_{GO\_TO\_SOURCE}$
R_7:			
$\mathcal{P}_7$	$P_{ON\_SOURCE} == True$	$P_{MOTIVATION_2} <= 0.5$	
$\mathcal{B}_7$	B_{RANDOM_WALK}	$B_{GO\_TO\_SOURCE}$	
$\mathcal{A}_7$	A_{IS}<br>A_{IS}<br>A_B	$p = 1.0$<br>$p = 0.01$<br>$p = 0.001$	$IS_{DROP\_FOOD} \leftarrow False$<br>$IS_{DROP\_FOOD} \leftarrow True$<br>$B_{RANDOM\_WALK}$
R_8:			
$\mathcal{P}_8$	$P_{HAS\_FOOD} == True$	$P_{MOTIVATION_1} <= 0.5$	
$\mathcal{B}_8$	$B_{GO\_TO\_SOURCE}$		
$\mathcal{A}_8$	A_B	$p = 0.001$	B_{RANDOM_WALK}
R_9:			
$\mathcal{P}_9$	$P_{ON\_SLOPE} == False$	$P_{ON\_NEST} == False$	
$\mathcal{B}_9$	$B_{GO\_TO\_SOURCE}$		
$\mathcal{A}_9$	A_B<br>A_{IS}<br>A_B	$p = 0.12125$<br>$p = 0.05$<br>$p = 0.005$	B_{RANDOM_WALK}<br>$IS_{DROP\_FOOD} \leftarrow False$<br>$B_{GO\_TO\_NEST}$
R_{10}:			
$\mathcal{P}_{10}$	$P_{HAS\_FOOD} == True$	$P_{MOTIVATION_1} <= 0.5$	$P_{ON\_NEST} == True$
$\mathcal{B}_{10}$	$B_{GO\_TO\_NEST}$	$B_{GO\_TO\_SOURCE}$	
$\mathcal{A}_{10}$	A_{IS}<br>A_{IS}	$p = 0.005$<br>$p = 0.1$	$IS_{WANT\_FOOD} \leftarrow True$<br>$IS_{MOTIVATION_2}$ $+$
R_{11}:			
$\mathcal{P}_{11}$	$P_{ON\_SLOPE} == True$	$P_{HAS\_FOOD} == True$	
$\mathcal{B}_{11}$	$B_{GO\_TO\_NEST}$		
$\mathcal{A}_{11}$	A_{IS}<br>A_B	$p = 1.0$<br>$p = 0.05$	$IS_{DROP\_FOOD} \leftarrow True$<br>$B_{GO\_TO\_NEST}$

Evolved controller 5

R_1:			
$\mathcal{P}_1$	$P_{MOTIVATION_1} <= 0.5$	$P_{ON\_GRASS} == False$	$P_{ON\_NEST} == True$
$\mathcal{B}_1$	B_{RANDOM_WALK}		
$\mathcal{A}_1$	A_{IS}	$p = 0.005$	$IS_{DROP\_FOOD} \leftarrow True$
	A_{IS}	$p = 1.0$	$IS_{MOTIVATION_1}$ $-$
	A_{IS}	$p = 0.001$	$IS_{DROP\_FOOD} \leftarrow False$
R_2:			
$\mathcal{P}_2$	$P_{HAS\_FOOD} == True$		
$\mathcal{B}_2$	B_{RANDOM_WALK}	$B_{GO\_TO\_SOURCE}$	
$\mathcal{A}_2$	A_B	$p = 0.05$	$B_{GO\_TO\_NEST}$
	A_B	$p = 0.001$	B_{RANDOM_WALK}
	A_B	$p = 0.075925$	$B_{GO\_TO\_SOURCE}$
R_3:			
$\mathcal{P}_3$	$P_{MOTIVATION_1} > 0.5$	$P_{ON\_GRASS} == False$	$P_{ON\_SOURCE} == False$
$\mathcal{B}_3$	$B_{GO\_TO\_NEST}$	$B_{GO\_TO\_SOURCE}$	
$\mathcal{A}_3$	A_B	$p = 0.1045$	$B_{GO\_TO\_SOURCE}$
	A_{IS}	$p = 1.0$	$IS_{DROP\_FOOD} \leftarrow True$
	A_{IS}	$p = 1.0$	$IS_{MOTIVATION_2}$ $+$
R_4:			
$\mathcal{P}_4$	$P_{ON\_SOURCE} == True$	$P_{MOTIVATION_1} <= 0.5$	
$\mathcal{B}_4$	$B_{GO\_TO\_NEST}$		
$\mathcal{A}_4$	A_B	$p = 0.01$	$B_{GO\_TO\_SOURCE}$
	A_{IS}	$p = 1.0$	$IS_{DROP\_FOOD} \leftarrow True$
R_5:			
$\mathcal{P}_5$	$P_{ON\_SLOPE} == False$		
$\mathcal{B}_5$	B_{RANDOM_WALK}	$B_{GO\_TO\_SOURCE}$	
$\mathcal{A}_5$	A_B	$p = 0.025$	B_{RANDOM_WALK}
	A_{IS}	$p = 1.0$	$IS_{MOTIVATION_1}$ $+$
R_6:			
$\mathcal{P}_6$	$P_{MOTIVATION_1} > 0.5$	$P_{HAS\_FOOD} == False$	
$\mathcal{B}_6$	B_{RANDOM_WALK}	$B_{GO\_TO\_NEST}$	$B_{GO\_TO\_SOURCE}$
$\mathcal{A}_6$	A_B	$p = 0.05$	$B_{GO\_TO\_SOURCE}$
R_7:			
$\mathcal{P}_7$	$P_{MOTIVATION_1} > 0.5$	$P_{MOTIVATION_1} <= 0.5$	$P_{ON\_SOURCE} == False$
$\mathcal{B}_7$	$B_{GO\_TO\_NEST}$		
$\mathcal{A}_7$	A_{IS}	$p = 0.05$	$IS_{WANT\_FOOD} \leftarrow True$
	A_{IS}	$p = 0.05$	$IS_{DROP\_FOOD} \leftarrow True$
	A_{IS}	$p = 0.01$	$IS_{WANT\_FOOD} \leftarrow False$
	A_{IS}	$p = 1.0$	$IS_{DROP\_FOOD} \leftarrow False$

Evolved controller 19

R_1:			
$\mathcal{P}_1$	$P_{ON\_SLOPE} == True$		
$\mathcal{B}_1$	$B_{GO\_TO\_NEST}$	$B_{GO\_TO\_SOURCE}$	
$\mathcal{A}_1$	A_B	$p = 0.1$	$B_{GO\_TO\_SOURCE}$
R_2:			
$\mathcal{P}_2$	$P_{ON\_NEST} == True$		
$\mathcal{B}_2$	$B_{GO\_TO\_NEST}$		
$\mathcal{A}_2$	A_{IS}<br>A_B<br>A_B	$p = 0.075$<br>$p = 0.001$<br>$p = 0.025$	$IS_{DROP\_FOOD} \leftarrow True$<br>$B_{GO\_TO\_SOURCE}$<br>$B_{GO\_TO\_NEST}$
R_3:			
$\mathcal{P}_3$	$P_{ON\_SLOPE} == False$		
$\mathcal{B}_3$	B_{RANDOM_WALK}	$B_{GO\_TO\_NEST}$	$B_{GO\_TO\_SOURCE}$
$\mathcal{A}_3$	A_{IS}<br>A_{IS}<br>A_{IS}<br>A_B	$p = 1.0$<br>$p = 0.005$<br>$p = 0.075$<br>$p = 0.01$	$IS_{MOTIVATION\_1} +$<br>$IS_{DROP\_FOOD} \leftarrow False$<br>$IS_{WANT\_FOOD} \leftarrow True$<br>$B_{GO\_TO\_NEST}$
R_4:			
$\mathcal{P}_4$	$P_{MOTIVATION\_1} > 0.5$	$P_{ON\_GRASS} == False$	
$\mathcal{B}_4$	$B_{GO\_TO\_SOURCE}$		
$\mathcal{A}_4$	A_B<br>A_{IS}	$p = 0.1$<br>$p = 0.075$	$B_{GO\_TO\_SOURCE}$<br>$IS_{DROP\_FOOD} \leftarrow True$
R_5:			
$\mathcal{P}_5$	$P_{ON\_SLOPE} == False$	$P_{HAS\_FOOD} == False$	
$\mathcal{B}_5$	B_{RANDOM_WALK}	$B_{GO\_TO\_NEST}$	
$\mathcal{A}_5$	A_B<br>A_B<br>A_{IS}	$p = 0.07375$<br>$p = 1.0$<br>$p = 0.1$	$B_{GO\_TO\_SOURCE}$<br>B_{RANDOM_WALK}<br>$IS_{DROP\_FOOD} \leftarrow True$
R_6:			
$\mathcal{P}_6$	$P_{ON\_SLOPE} == False$	$P_{HAS\_FOOD} == True$	
$\mathcal{B}_6$	$B_{GO\_TO\_SOURCE}$		
$\mathcal{A}_6$	A_B<br>A_B<br>A_{IS}	$p = 0.1225$<br>$p = 0.01$<br>$p = 0.005$	$B_{GO\_TO\_NEST}$<br>$B_{GO\_TO\_SOURCE}$<br>$IS_{DROP\_FOOD} \leftarrow True$

Evolved controller 11

R_1:			
$\mathcal{P}_1$	$P_{ON\_SLOPE} == False$	$P_{ON\_NEST} == False$	
$\mathcal{B}_1$	B_{RANDOM_WALK}	$B_{GO\_TO\_SOURCE}$	
$\mathcal{A}_1$	A_{IS}<br>A_B	$p = 0.1$<br>$p = 0.12125$	$IS_{MOTIVATION_2}$ +<br>$B_{GO\_TO\_SOURCE}$
R_2:			
$\mathcal{P}_2$	$P_{ON\_NEST} == True$	$P_{ON\_SOURCE} == False$	
$\mathcal{B}_2$	B_{RANDOM_WALK}	$B_{GO\_TO\_NEST}$	
$\mathcal{A}_2$	A_B<br>A_{IS}	$p = 0.075$<br>$p = 0.075$	$B_{GO\_TO\_SOURCE}$<br>$IS_{MOTIVATION_2}$ +
R_3:			
$\mathcal{P}_3$	$P_{ON\_SLOPE} == False$		
$\mathcal{B}_3$	$B_{GO\_TO\_NEST}$	$B_{GO\_TO\_SOURCE}$	
$\mathcal{A}_3$	A_{IS}<br>A_B<br>A_B	$p = 0.001$<br>$p = 0.1$<br>$p = 0.01$	$IS_{WANT\_FOOD} \leftarrow True$<br>$B_{RANDOM\_WALK}$<br>$B_{GO\_TO\_SOURCE}$
R_4:			
$\mathcal{P}_4$	$P_{ON\_SLOPE} == False$	$P_{HAS\_FOOD} == True$	
$\mathcal{B}_4$	$B_{GO\_TO\_NEST}$	$B_{GO\_TO\_SOURCE}$	
$\mathcal{A}_4$	A_{IS}<br>A_B<br>A_B<br>A_{IS}	$p = 0.01$<br>$p = 1.0$<br>$p = 0.005$<br>$p = 0.075$	$IS_{MOTIVATION_1}$ −<br>$B_{GO\_TO\_NEST}$<br>$B_{GO\_TO\_SOURCE}$<br>$IS_{MOTIVATION_1}$ +
R_5:			
$\mathcal{P}_5$	$P_{HAS\_FOOD} == False$	$P_{ON\_NEST} == True$	
$\mathcal{B}_5$	B_{RANDOM_WALK}	$B_{GO\_TO\_NEST}$	$B_{GO\_TO\_SOURCE}$
$\mathcal{A}_5$	A_B	$p = 0.01$	$B_{GO\_TO\_NEST}$
R_6:			
$\mathcal{P}_6$	$P_{MOTIVATION_2} > 0.5$	$P_{ON\_NEST} == True$	
$\mathcal{B}_6$	B_{RANDOM_WALK}	$B_{GO\_TO\_NEST}$	
$\mathcal{A}_6$	A_{IS}<br>A_{IS}<br>A_B	$p = 0.1$<br>$p = 0.05$<br>$p = 0.075$	$IS_{DROP\_FOOD} \leftarrow True$<br>$IS_{MOTIVATION_2}$ +<br>$B_{GO\_TO\_SOURCE}$
R_7:			
$\mathcal{P}_7$	$P_{ON\_SLOPE} == True$	$P_{ON\_SOURCE} == False$	
$\mathcal{B}_7$	$B_{GO\_TO\_NEST}$		
$\mathcal{A}_7$	A_{IS}<br>A_B	$p = 1.0$<br>$p = 0.1$	$IS_{DROP\_FOOD} \leftarrow True$<br>$B_{GO\_TO\_SOURCE}$

Evolved controller 2

R_1:			
$\mathcal{P}_1$	$P_{ON\_GRASS} == False$	$P_{ON\_SOURCE} == False$	
$\mathcal{B}_1$	$B_{GO\_TO\_NEST}$		
$\mathcal{A}_1$	A_B<br>A_{IS}	$p = 1.0$<br>$p = 1.0$	$B_{GO\_TO\_SOURCE}$<br>$IS_{DROP\_FOOD} \leftarrow True$
R_2:			
$\mathcal{P}_2$	$P_{ON\_SLOPE} == False$		
$\mathcal{B}_2$	B_{RANDOM_WALK}	$B_{GO\_TO\_SOURCE}$	
$\mathcal{A}_2$	A_{IS}<br>A_B<br>A_B	$p = 0.08425$<br>$p = 0.005$<br>$p = 0.001$	$IS_{DROP\_FOOD} \leftarrow True$<br>$B_{GO\_TO\_SOURCE}$<br>$B_{RANDOM\_WALK}$
R_3:			
$\mathcal{P}_3$	$P_{ON\_NEST} == True$	$P_{ON\_NEST} == False$	$P_{MOTIVATION_2} <= 0.5$
$\mathcal{B}_3$	B_{RANDOM_WALK}	$B_{GO\_TO\_SOURCE}$	
$\mathcal{A}_3$	A_B<br>A_{IS}<br>A_{IS}	$p = 1.0$<br>$p = 0.025$<br>$p = 1.0$	$B_{GO\_TO\_SOURCE}$<br>$IS_{WANT\_FOOD} \leftarrow True$<br>$IS_{DROP\_FOOD} \leftarrow False$
R_4:			
$\mathcal{P}_4$	$P_{ON\_SLOPE} == False$	$P_{HAS\_FOOD} == True$	$P_{ON\_NEST} == False$
$\mathcal{B}_4$	B_{RANDOM_WALK}	$B_{GO\_TO\_SOURCE}$	
$\mathcal{A}_4$	A_B<br>A_B<br>A_{IS}	$p = 0.144375$<br>$p = 0.005$<br>$p = 0.025$	$B_{GO\_TO\_NEST}$<br>B_{RANDOM_WALK}<br>$IS_{MOTIVATION_2} +$

Evolved controller 3

R_1:			
$\mathcal{P}_1$	$P_{MOTIVATION_1} <= 0.5$	$P_{ON\_SOURCE} == False$	
$\mathcal{B}_1$	$B_{GO\_TO\_NEST}$		
$\mathcal{A}_1$	A_B	$p = 0.19$	$B_{GO\_TO\_SOURCE}$
	A_{IS}	$p = 0.1$	$IS_{WANT\_FOOD} \leftarrow True$
R_2:			
$\mathcal{P}_2$	$P_{HAS\_FOOD} == True$	$P_{ON\_GRASS} == False$	
$\mathcal{B}_2$	$B_{GO\_TO\_SOURCE}$		
$\mathcal{A}_2$	A_B	$p = 0.1$	$B_{GO\_TO\_SOURCE}$
	A_{IS}	$p = 1.0$	$IS_{DROP\_FOOD} \leftarrow True$
R_3:			
$\mathcal{P}_3$	$P_{ON\_GRASS} == True$		
$\mathcal{B}_3$	B_{RANDOM_WALK}	$B_{GO\_TO\_NEST}$	
$\mathcal{A}_3$	A_B	$p = 1.0$	B_{RANDOM_WALK}
	A_B	$p = 0.025$	$B_{GO\_TO\_SOURCE}$
R_4:			
$\mathcal{P}_4$	$P_{ON\_SOURCE} == True$		
$\mathcal{B}_4$	$B_{GO\_TO\_SOURCE}$		
$\mathcal{A}_4$	A_B	$p = 0.001$	B_{RANDOM_WALK}
	A_{IS}	$p = 0.05$	$IS_{DROP\_FOOD} \leftarrow True$
R_5:			
$\mathcal{P}_5$	$P_{HAS\_FOOD} == True$		
$\mathcal{B}_5$	B_{RANDOM_WALK}	$B_{GO\_TO\_SOURCE}$	
$\mathcal{A}_5$	A_{IS}	$p = 0.1$	$IS_{WANT\_FOOD} \leftarrow True$
	A_B	$p = 1.0$	$B_{GO\_TO\_NEST}$
R_6:			
$\mathcal{P}_6$	$P_{ON\_SLOPE} == True$	$P_{ON\_GRASS} == True$	
$\mathcal{B}_6$	$B_{GO\_TO\_NEST}$		
$\mathcal{A}_6$	A_{IS}	$p = 0.001$	$IS_{DROP\_FOOD} \leftarrow False$

Evolved controller 14

R_1:			
$\mathcal{P}_1$	$P_{ON\_SLOPE} == True$	$P_{HAS\_FOOD} == True$	$P_{ON\_GRASS} == True$
$\mathcal{B}_1$	B_{RANDOM_WALK}		
$\mathcal{A}_1$	A_{IS}	$p = 0.1$	$IS_{MOTIVATION_1}$ −
R_2:			
$\mathcal{P}_2$	$P_{ON\_SOURCE} == True$	$P_{ON\_NEST} == False$	
$\mathcal{B}_2$	$B_{GO\_TO\_SOURCE}$		
$\mathcal{A}_2$	A_B<br>A_B	$p = 0.12125$<br>$p = 0.01099$	$B_{GO\_TO\_SOURCE}$<br>B_{RANDOM_WALK}
R_3:			
$\mathcal{P}_3$	$P_{ON\_SOURCE} == False$		
$\mathcal{B}_3$	B_{RANDOM_WALK}		
$\mathcal{A}_3$	A_B<br>A_{IS}	$p = 0.075$<br>$p = 0.075$	$B_{GO\_TO\_SOURCE}$<br>$IS_{DROP\_FOOD} \leftarrow True$
R_4:			
$\mathcal{P}_4$	$P_{HAS\_FOOD} == True$		
$\mathcal{B}_4$	$B_{GO\_TO\_NEST}$	$B_{GO\_TO\_SOURCE}$	
$\mathcal{A}_4$	A_B<br>A_B	$p = 0.01$<br>$p = 0.1009$	B_{RANDOM_WALK}<br>$B_{GO\_TO\_NEST}$

Evolved controller 15

R_1:			
$\mathcal{P}_1$	$P_{ON\_SLOPE} == False$	$P_{ON\_SOURCE} == True$	$P_{HAS\_FOOD} == True$
$\mathcal{B}_1$	B_{RANDOM_WALK}	$B_{GO\_TO\_SOURCE}$	
$\mathcal{A}_1$	A_B<br>A_{IS}<br>A_B	$p = 0.05$<br>$p = 0.025$<br>$p = 0.075$	$B_{GO\_TO\_SOURCE}$<br>$IS_{MOTIVATION_2}$ $-$<br>$B_{GO\_TO\_NEST}$
R_2:			
$\mathcal{P}_2$	$P_{ON\_SOURCE} == False$		
$\mathcal{B}_2$	B_{RANDOM_WALK}	$B_{GO\_TO\_NEST}$	$B_{GO\_TO\_SOURCE}$
$\mathcal{A}_2$	A_{IS}	$p = 0.075$	$IS_{MOTIVATION_1}$ $-$
R_3:			
$\mathcal{P}_3$	$P_{ON\_NEST} == True$		
$\mathcal{B}_3$	B_{RANDOM_WALK}	$B_{GO\_TO\_NEST}$	
$\mathcal{A}_3$	A_B<br>A_B<br>A_{IS}<br>A_{IS}	$p = 0.001$<br>$p = 0.1$<br>$p = 1.0$<br>$p = 0.001$	$B_{GO\_TO\_SOURCE}$<br>$B_{GO\_TO\_NEST}$<br>$IS_{DROP\_FOOD} \leftarrow True$<br>$IS_{MOTIVATION_2}$ $-$
R_4:			
$\mathcal{P}_4$	$P_{HAS\_FOOD} == False$		
$\mathcal{B}_4$	B_{RANDOM_WALK}	$B_{GO\_TO\_NEST}$	
$\mathcal{A}_4$	A_{IS}<br>A_B<br>A_{IS}	$p = 0.005$<br>$p = 0.025$<br>$p = 0.05$	$IS_{MOTIVATION_2}$ $-$<br>$B_{GO\_TO\_SOURCE}$<br>$IS_{WANT\_FOOD} \leftarrow True$
R_5:			
$\mathcal{P}_5$	$P_{ON\_SOURCE} == True$	$P_{ON\_GRASS} == False$	
$\mathcal{B}_5$	$B_{GO\_TO\_SOURCE}$		
$\mathcal{A}_5$	A_{IS}<br>A_B<br>A_{IS}	$p = 0.005$<br>$p = 0.01099$<br>$p = 0.005$	$IS_{MOTIVATION_1}$ $+$<br>B_{RANDOM_WALK}<br>$IS_{WANT\_FOOD} \leftarrow True$

Evolved controller 12

R_1:			
$\mathcal{P}_1$	$P_{ON\_SLOPE} == True$		
$\mathcal{B}_1$	B_{RANDOM_WALK}	$B_{GO\_TO\_NEST}$	
$\mathcal{A}_1$	A_{IS}<br>A_{IS}<br>A_{IS}	$p = 0.025$<br>$p = 0.01$<br>$p = 0.005$	$IS_{WANT\_FOOD} \leftarrow False$<br>$IS_{DROP\_FOOD} \leftarrow False$<br>$IS_{MOTIVATION_2}$ +
R_2:			
$\mathcal{P}_2$	$P_{MOTIVATION_2} <= 0.5$		
$\mathcal{B}_2$	$B_{GO\_TO\_SOURCE}$		
$\mathcal{A}_2$	A_{IS}	$p = 0.075$	$IS_{WANT\_FOOD} \leftarrow True$
R_3:			
$\mathcal{P}_3$	$P_{HAS\_FOOD} == True$		
$\mathcal{B}_3$	B_{RANDOM_WALK}	$B_{GO\_TO\_SOURCE}$	
$\mathcal{A}_3$	A_{IS}<br>A_B	$p = 0.005$<br>$p = 1.0$	$IS_{DROP\_FOOD} \leftarrow False$<br>$B_{GO\_TO\_NEST}$
R_4:			
$\mathcal{P}_4$	$P_{ON\_SLOPE} == False$		
$\mathcal{B}_4$	B_{RANDOM_WALK}	$B_{GO\_TO\_SOURCE}$	
$\mathcal{A}_4$	A_B<br>A_{IS}<br>A_B	$p = 0.01$<br>$p = 0.025$<br>$p = 0.075$	B_{RANDOM_WALK}<br>$IS_{MOTIVATION_1}$ +<br>$B_{GO\_TO\_SOURCE}$
R_5:			
$\mathcal{P}_5$	$P_{ON\_NEST} == True$		
$\mathcal{B}_5$	B_{RANDOM_WALK}	$B_{GO\_TO\_NEST}$	$B_{GO\_TO\_SOURCE}$
$\mathcal{A}_5$	A_B<br>A_{IS}	$p = 0.1009$<br>$p = 0.1675$	B_{RANDOM_WALK}<br>$IS_{DROP\_FOOD} \leftarrow True$
R_6:			
$\mathcal{P}_6$	$P_{ON\_SLOPE} == False$	$P_{ON\_GRASS} == False$	
$\mathcal{B}_6$	$B_{GO\_TO\_NEST}$	$B_{GO\_TO\_SOURCE}$	
$\mathcal{A}_6$	A_{IS}<br>A_B<br>A_{IS}	$p = 0.01$<br>$p = 0.01$<br>$p = 0.1$	$IS_{MOTIVATION_2}$ +<br>$B_{GO\_TO\_SOURCE}$<br>$IS_{WANT\_FOOD} \leftarrow True$
R_7:			
$\mathcal{P}_7$	$P_{ON\_SLOPE} == True$	$P_{MOTIVATION_2} > 0.5$	$P_{ON\_NEST} == True$
$\mathcal{B}_7$	B_{RANDOM_WALK}		
$\mathcal{A}_7$	A_{IS}<br>A_B	$p = 0.075$<br>$p = 0.1$	$IS_{MOTIVATION_2}$ −<br>$B_{GO\_TO\_NEST}$

Evolved controller 22

R_1:			
$\mathcal{P}_1$	$P_{ON\_NEST} == True$	$P_{MOTIVATION_2} <= 0.5$	
$\mathcal{B}_1$	B_{RANDOM_WALK}	$B_{GO\_TO\_NEST}$	
$\mathcal{A}_1$	A_B<br>A_B<br>A_{IS}	$p = 1.0$<br>$p = 0.075925$<br>$p = 1.0$	$B_{GO\_TO\_SOURCE}$<br>B_{RANDOM_WALK}<br>$IS_{DROP\_FOOD} \leftarrow True$
R_2:			
$\mathcal{P}_2$	$P_{ON\_NEST} == False$	$P_{ON\_GRASS} == True$	
$\mathcal{B}_2$	$B_{GO\_TO\_SOURCE}$		
$\mathcal{A}_2$	A_{IS}	$p = 1.0$	$IS_{DROP\_FOOD} \leftarrow True$
R_3:			
$\mathcal{P}_3$	$P_{ON\_SLOPE} == False$	$P_{HAS\_FOOD} == True$	
$\mathcal{B}_3$	B_{RANDOM_WALK}	$B_{GO\_TO\_SOURCE}$	
$\mathcal{A}_3$	A_B<br>A_{IS}<br>A_B	$p = 0.1$<br>$p = 1.0$<br>$p = 0.1$	B_{RANDOM_WALK}<br>$IS_{MOTIVATION_1} +$<br>$B_{GO\_TO\_NEST}$
R_4:			
$\mathcal{P}_4$	$P_{ON\_SOURCE} == True$	$P_{HAS\_FOOD} == False$	
$\mathcal{B}_4$	B_{RANDOM_WALK}	$B_{GO\_TO\_NEST}$	$B_{GO\_TO\_SOURCE}$
$\mathcal{A}_4$	A_B<br>A_B	$p = 0.098125$<br>$p = 0.05$	$B_{GO\_TO\_SOURCE}$<br>B_{RANDOM_WALK}

Evolved controller 4

R_1:			
$\mathcal{P}_1$	$P_{ON\_SLOPE} == True$		
$\mathcal{B}_1$	B_{RANDOM_WALK}	$B_{GO\_TO\_NEST}$	
$\mathcal{A}_1$	A_{IS}<br>A_B<br>A_{IS}	$p = 1.0$<br>$p = 0.025$<br>$p = 0.01$	$IS_{DROP\_FOOD} \leftarrow True$<br>$B_{RANDOM\_WALK}$<br>$IS_{DROP\_FOOD} \leftarrow False$
R_2:			
$\mathcal{P}_2$	$P_{MOTIVATION_1} > 0.5$		
$\mathcal{B}_2$	B_{RANDOM_WALK}	$B_{GO\_TO\_NEST}$	
$\mathcal{A}_2$	A_{IS}<br>A_{IS}<br>A_{IS}<br>A_B	$p = 0.025$<br>$p = 0.01$<br>$p = 0.005$<br>$p = 0.025$	$IS_{WANT\_FOOD} \leftarrow True$<br>$IS_{DROP\_FOOD} \leftarrow False$<br>$IS_{DROP\_FOOD} \leftarrow True$<br>$B_{GO\_TO\_SOURCE}$
R_3:			
$\mathcal{P}_3$	$P_{HAS\_FOOD} == True$	$P_{MOTIVATION_1} > 0.5$	
$\mathcal{B}_3$	$B_{GO\_TO\_SOURCE}$		
$\mathcal{A}_3$	A_B<br>A_{IS}	$p = 1.0$<br>$p = 0.075$	$B_{GO\_TO\_NEST}$<br>$IS_{DROP\_FOOD} \leftarrow False$
R_4:			
$\mathcal{P}_4$	$P_{MOTIVATION_1} <= 0.5$		
$\mathcal{B}_4$	B_{RANDOM_WALK}	$B_{GO\_TO\_NEST}$	
$\mathcal{A}_4$	A_B<br>A_{IS}<br>A_{IS}	$p = 0.144375$<br>$p = 0.075$<br>$p = 0.001$	B_{RANDOM_WALK}<br>$IS_{MOTIVATION_1}$ +<br>$IS_{MOTIVATION_2}$ −
R_5:			
$\mathcal{P}_5$	$P_{ON\_SOURCE} == True$	$P_{MOTIVATION_1} > 0.5$	$P_{ON\_GRASS} == False$
$\mathcal{B}_5$	B_{RANDOM_WALK}	$B_{GO\_TO\_SOURCE}$	
$\mathcal{A}_5$	A_B<br>A_{IS}<br>A_B	$p = 0.1$<br>$p = 0.001$<br>$p = 0.1$	$B_{GO\_TO\_SOURCE}$<br>$IS_{MOTIVATION_1}$ +<br>B_{RANDOM_WALK}

Evolved controller 8

R_1:			
$\mathcal{P}_1$	$P_{ON\_SLOPE} == True$	$P_{HAS\_FOOD} == True$	$P_{ON\_GRASS} == True$
$\mathcal{B}_1$	B_{RANDOM_WALK}		
$\mathcal{A}_1$	A_{IS}	$p = 0.1$	$IS_{MOTIVATION_2}$ −
R_2:			
$\mathcal{P}_2$	$P_{ON\_SLOPE} == False$	$P_{ON\_SOURCE} == True$	
$\mathcal{B}_2$	$B_{GO\_TO\_SOURCE}$		
$\mathcal{A}_2$	A_B<br>A_B	$p = 0.144375$<br>$p = 0.01099$	$B_{GO\_TO\_SOURCE}$<br>B_{RANDOM_WALK}
R_3:			
$\mathcal{P}_3$	$P_{ON\_SOURCE} == False$		
$\mathcal{B}_3$	B_{RANDOM_WALK}		
$\mathcal{A}_3$	A_B<br>A_{IS}	$p = 0.025$<br>$p = 0.025$	$B_{GO\_TO\_SOURCE}$<br>$IS_{DROP\_FOOD} \leftarrow True$
R_4:			
$\mathcal{P}_4$	$P_{HAS\_FOOD} == True$		
$\mathcal{B}_4$	$B_{GO\_TO\_NEST}$	$B_{GO\_TO\_SOURCE}$	
$\mathcal{A}_4$	A_B<br>A_B	$p = 0.01$<br>$p = 0.1009$	B_{RANDOM_WALK}<br>$B_{GO\_TO\_NEST}$

Evolved controller 16

R_1:			
$\mathcal{P}_1$	$P_{ON\_SLOPE} == False$	$P_{ON\_GRASS} == False$	
$\mathcal{B}_1$	$B_{GO\_TO\_SOURCE}$		
$\mathcal{A}_1$	A_B	$p = 1.0$	B_{RANDOM_WALK}
R_2:			
$\mathcal{P}_2$	$P_{ON\_SLOPE} == False$	$P_{HAS\_FOOD} == True$	$P_{HAS\_FOOD} == False$
$\mathcal{B}_2$	$B_{GO\_TO\_SOURCE}$		
$\mathcal{A}_2$	A_{IS}<br>A_B<br>A_{IS}<br>A_{IS}	$p = 0.1$<br>$p = 0.075$<br>$p = 0.01$<br>$p = 0.005$	$IS_{WANT\_FOOD} \leftarrow False$<br>$B_{GO\_TO\_NEST}$<br>$IS_{MOTIVATION_1} -$<br>$IS_{DROP\_FOOD} \leftarrow True$
R_3:			
$\mathcal{P}_3$	$P_{ON\_SOURCE} == False$	$P_{ON\_NEST} == True$	$P_{MOTIVATION_2} <= 0.5$
$\mathcal{B}_3$	B_{RANDOM_WALK}	$B_{GO\_TO\_NEST}$	
$\mathcal{A}_3$	A_{IS}<br>A_{IS}	$p = 0.1$<br>$p = 0.005$	$IS_{DROP\_FOOD} \leftarrow True$<br>$IS_{MOTIVATION_2} +$
R_4:			
$\mathcal{P}_4$	$P_{HAS\_FOOD} == True$		
$\mathcal{B}_4$	B_{RANDOM_WALK}	$B_{GO\_TO\_SOURCE}$	
$\mathcal{A}_4$	A_{IS}	$p = 0.005$	$IS_{MOTIVATION_2} +$
R_5:			
$\mathcal{P}_5$	$P_{MOTIVATION_2} <= 0.5$	$P_{ON\_GRASS} == False$	$P_{HAS\_FOOD} == False$
$\mathcal{B}_5$	$B_{GO\_TO\_SOURCE}$		
$\mathcal{A}_5$	A_{IS}	$p = 0.01$	$IS_{MOTIVATION_1} -$
R_6:			
$\mathcal{P}_6$	$P_{HAS\_FOOD} == False$		
$\mathcal{B}_6$	B_{RANDOM_WALK}	$B_{GO\_TO\_NEST}$	
$\mathcal{A}_6$	A_{IS}<br>A_B	$p = 0.01$<br>$p = 1.0$	$IS_{MOTIVATION_1} -$<br>$B_{GO\_TO\_SOURCE}$
R_7:			
$\mathcal{P}_7$	$P_{ON\_SLOPE} == False$		
$\mathcal{B}_7$	B_{RANDOM_WALK}	$B_{GO\_TO\_SOURCE}$	
$\mathcal{A}_7$	A_B<br>A_{IS}<br>A_B	$p = 0.049375$<br>$p = 1.0$<br>$p = 0.075$	$B_{GO\_TO\_NEST}$<br>$IS_{DROP\_FOOD} \leftarrow False$<br>$B_{RANDOM\_WALK}$
R_8:			
$\mathcal{P}_8$	$P_{ON\_SLOPE} == True$	$P_{MOTIVATION_1} > 0.5$	$P_{ON\_NEST} == True$
$\mathcal{B}_8$	$B_{GO\_TO\_NEST}$	$B_{GO\_TO\_SOURCE}$	
$\mathcal{A}_8$	A_B	$p = 0.075$	$B_{GO\_TO\_NEST}$

Evolved controller 18

R_1:			
$\mathcal{P}_1$	$P_{ON\_SOURCE} == True$	$P_{HAS\_FOOD} == True$	$P_{ON\_GRASS} == False$
$\mathcal{B}_1$	$B_{GO\_TO\_NEST}$	$B_{GO\_TO\_SOURCE}$	
$\mathcal{A}_1$	A_{IS}<br>A_B<br>A_B	$p = 0.1$<br>$p = 0.01$<br>$p = 1.0$	$IS_{MOTIVATION_1}$ +<br>$B_{GO\_TO\_SOURCE}$<br>$B_{GO\_TO\_NEST}$
R_2:			
$\mathcal{P}_2$	$P_{ON\_SLOPE} == False$	$P_{HAS\_FOOD} == False$	$P_{ON\_SOURCE} == False$
$\mathcal{B}_2$	B_{RANDOM_WALK}		
$\mathcal{A}_2$	A_B<br>A_{IS}<br>A_B	$p = 0.075$<br>$p = 0.075$<br>$p = 0.005$	$B_{GO\_TO\_SOURCE}$<br>$IS_{DROP\_FOOD} \leftarrow True$<br>$B_{GO\_TO\_NEST}$
R_3:			
$\mathcal{P}_3$	$P_{ON\_NEST} == False$	$P_{ON\_SOURCE} == False$	
$\mathcal{B}_3$	B_{RANDOM_WALK}	$B_{GO\_TO\_SOURCE}$	
$\mathcal{A}_3$	A_B	$p = 0.075$	$B_{GO\_TO\_SOURCE}$
R_4:			
$\mathcal{P}_4$	$P_{ON\_SLOPE} == False$	$P_{ON\_NEST} == True$	
$\mathcal{B}_4$	$B_{GO\_TO\_NEST}$		
$\mathcal{A}_4$	A_{IS}<br>A_B<br>A_{IS}	$p = 0.075$<br>$p = 0.1$<br>$p = 1.0$	$IS_{WANT\_FOOD} \leftarrow True$<br>$B_{GO\_TO\_SOURCE}$<br>$IS_{DROP\_FOOD} \leftarrow True$

Evolved controller 21

R_1:			
$\mathcal{P}_1$	$P_{ON\_SLOPE} == False$	$P_{ON\_SOURCE} == True$	$P_{HAS\_FOOD} == True$
$\mathcal{B}_1$	$B_{GO\_TO\_NEST}$	$B_{GO\_TO\_SOURCE}$	
$\mathcal{A}_1$	A_{IS}<br>A_B<br>A_B	$p = 0.025$<br>$p = 0.001$<br>$p = 1.0$	$IS_{WANT\_FOOD} \leftarrow True$<br>$B_{GO\_TO\_SOURCE}$<br>$B_{GO\_TO\_NEST}$
R_2:			
$\mathcal{P}_2$	$P_{ON\_GRASS} == False$	$P_{ON\_NEST} == True$	$P_{ON\_SOURCE} == False$
$\mathcal{B}_2$	B_{RANDOM_WALK}	$B_{GO\_TO\_NEST}$	
$\mathcal{A}_2$	A_{IS}<br>A_B<br>A_{IS}<br>A_{IS}	$p = 0.05$<br>$p = 1.0$<br>$p = 1.0$<br>$p = 0.1$	$IS_{MOTIVATION_2} -$<br>$B_{GO\_TO\_SOURCE}$<br>$IS_{DROP\_FOOD} \leftarrow True$<br>$IS_{WANT\_FOOD} \leftarrow True$
R_3:			
$\mathcal{P}_3$	$P_{ON\_SOURCE} == True$	$P_{HAS\_FOOD} == True$	$P_{HAS\_FOOD} == False$
$\mathcal{B}_3$	$B_{GO\_TO\_NEST}$	$B_{GO\_TO\_SOURCE}$	
$\mathcal{A}_3$	A_{IS}<br>A_{IS}<br>A_B	$p = 0.075$<br>$p = 0.01$<br>$p = 0.05475$	$IS_{MOTIVATION_1} -$<br>$IS_{MOTIVATION_2} +$<br>$B_{GO\_TO\_NEST}$

Evolved controller 7

R_1:			
$\mathcal{P}_1$	$P_{ON\_NEST} == True$	$P_{ON\_NEST} == False$	
$\mathcal{B}_1$	B_{RANDOM_WALK}		
$\mathcal{A}_1$	A_B	$p = 0.001$	$B_{GO\_TO\_NEST}$
R_2:			
$\mathcal{P}_2$	$P_{ON\_SLOPE} == False$		
$\mathcal{B}_2$	$B_{GO\_TO\_NEST}$	$B_{GO\_TO\_SOURCE}$	
$\mathcal{A}_2$	A_{IS}<br>A_B<br>A_{IS}	$p = 0.005$<br>$p = 0.01$<br>$p = 0.075$	$IS_{DROP\_FOOD} \leftarrow True$<br>$B_{GO\_TO\_NEST}$<br>$IS_{MOTIVATION_1} -$
R_3:			
$\mathcal{P}_3$	$P_{ON\_SLOPE} == False$	$P_{HAS\_FOOD} == False$	$P_{ON\_SOURCE} == False$
$\mathcal{B}_3$	B_{RANDOM_WALK}	$B_{GO\_TO\_NEST}$	$B_{GO\_TO\_SOURCE}$
$\mathcal{A}_3$	A_{IS}<br>A_B	$p = 0.01$<br>$p = 1.0$	$IS_{MOTIVATION_1} -$<br>$B_{GO\_TO\_SOURCE}$
R_4:			
$\mathcal{P}_4$	$P_{HAS\_FOOD} == False$		
$\mathcal{B}_4$	$B_{GO\_TO\_NEST}$	$B_{GO\_TO\_SOURCE}$	
$\mathcal{A}_4$	A_{IS}<br>A_B	$p = 0.1$<br>$p = 0.1$	$IS_{MOTIVATION_1} +$<br>$B_{GO\_TO\_SOURCE}$

Evolved controller 13

R_1:			
$\mathcal{P}_1$	$P_{ON\_SLOPE} == True$	$P_{MOTIVATION_2} > 0.5$	$P_{ON\_GRASS} == True$
$\mathcal{B}_1$	B_{RANDOM_WALK}	$B_{GO\_TO\_NEST}$	$B_{GO\_TO\_SOURCE}$
$\mathcal{A}_1$	A_{IS}<br>A_B<br>A_B	$p = 0.025$<br>$p = 0.025$<br>$p = 0.05$	$IS_{MOTIVATION_2} +$<br>$B_{GO\_TO\_SOURCE}$<br>$B_{GO\_TO\_NEST}$
R_2:			
$\mathcal{P}_2$	$P_{HAS\_FOOD} == True$		
$\mathcal{B}_2$	$B_{GO\_TO\_NEST}$	$B_{GO\_TO\_SOURCE}$	
$\mathcal{A}_2$	A_B	$p = 0.0595$	$B_{GO\_TO\_NEST}$
R_3:			
$\mathcal{P}_3$	$P_{ON\_SOURCE} == False$		
$\mathcal{B}_3$	B_{RANDOM_WALK}	$B_{GO\_TO\_NEST}$	
$\mathcal{A}_3$	A_{IS}<br>A_B<br>A_B	$p = 0.005$<br>$p = 0.1$<br>$p = 0.005$	$IS_{DROP\_FOOD} \leftarrow True$<br>$B_{GO\_TO\_NEST}$<br>$B_{GO\_TO\_SOURCE}$

Evolved controller 6

R_1:			
$\mathcal{P}_1$	$P_{HAS\_FOOD} == True$	$P_{HAS\_FOOD} == False$	$P_{MOTIVATION_2} <= 0.5$
$\mathcal{B}_1$	B_{RANDOM_WALK}	$B_{GO\_TO\_SOURCE}$	
$\mathcal{A}_1$	A_B	$p = 0.1$	$B_{GO\_TO\_NEST}$
R_2:			
$\mathcal{P}_2$	$P_{ON\_SOURCE} == True$	$P_{MOTIVATION_1} > 0.5$	$P_{HAS\_FOOD} == False$
$\mathcal{B}_2$	B_{RANDOM_WALK}	$B_{GO\_TO\_NEST}$	$B_{GO\_TO\_SOURCE}$
$\mathcal{A}_2$	A_{IS}	$p = 0.025$	$IS_{DROP\_FOOD} \leftarrow False$
R_3:			
$\mathcal{P}_3$	$P_{HAS\_FOOD} == True$		
$\mathcal{B}_3$	$B_{GO\_TO\_SOURCE}$		
$\mathcal{A}_3$	A_B<br>A_{IS}	$p = 0.049375$<br>$p = 0.1$	$B_{GO\_TO\_NEST}$<br>$IS_{DROP\_FOOD} \leftarrow True$
R_4:			
$\mathcal{P}_4$	$P_{ON\_NEST} == True$		
$\mathcal{B}_4$	B_{RANDOM_WALK}	$B_{GO\_TO\_NEST}$	
$\mathcal{A}_4$	A_B<br>A_{IS}<br>A_B	$p = 0.029875$<br>$p = 0.1$<br>$p = 0.005$	$B_{GO\_TO\_SOURCE}$<br>$IS_{MOTIVATION_2}\ +$<br>$B_{GO\_TO\_NEST}$
R_5:			
$\mathcal{P}_5$	$P_{MOTIVATION_1} > 0.5$	$P_{MOTIVATION_1} <= 0.5$	
$\mathcal{B}_5$	$B_{GO\_TO\_NEST}$	$B_{GO\_TO\_SOURCE}$	
$\mathcal{A}_5$	A_B<br>A_{IS}	$p = 1.0$<br>$p = 0.01$	B_{RANDOM_WALK}<br>$IS_{MOTIVATION_2}\ -$
R_6:			
$\mathcal{P}_6$	$P_{ON\_SLOPE} == True$	$P_{HAS\_FOOD} == True$	
$\mathcal{B}_6$	$B_{GO\_TO\_NEST}$	$B_{GO\_TO\_SOURCE}$	
$\mathcal{A}_6$	A_B<br>A_{IS}<br>A_B<br>A_B	$p = 0.01$<br>$p = 0.075$<br>$p = 0.075$<br>$p = 0.05$	B_{RANDOM_WALK}<br>$IS_{MOTIVATION_1}\ -$<br>$B_{GO\_TO\_SOURCE}$<br>$B_{GO\_TO\_NEST}$
R_7:			
$\mathcal{P}_7$	$P_{ON\_SLOPE} == True$		
$\mathcal{B}_7$	B_{RANDOM_WALK}	$B_{GO\_TO\_SOURCE}$	
$\mathcal{A}_7$	A_{IS}<br>A_B	$p = 0.05$<br>$p = 0.075$	$IS_{MOTIVATION_2}\ -$<br>$B_{GO\_TO\_SOURCE}$
R_8:			
$\mathcal{P}_8$	$P_{ON\_SLOPE} == False$	$P_{ON\_SOURCE} == True$	$P_{ON\_SLOPE} == True$
$\mathcal{B}_8$	B_{RANDOM_WALK}	$B_{GO\_TO\_NEST}$	
$\mathcal{A}_8$	A_{IS}	$p = 0.1$	$IS_{WANT\_FOOD} \leftarrow False$

Evolved controller 17

R_1:			
$\mathcal{P}_1$	$P_{ON\_GRASS} == False$		
$\mathcal{B}_1$	$B_{GO\_TO\_NEST}$		
$\mathcal{A}_1$	A_B<br>A_B<br>A_{IS}	$p = 0.075$<br>$p = 0.005$<br>$p = 0.001$	$B_{GO\_TO\_NEST}$<br>$B_{GO\_TO\_SOURCE}$<br>$IS_{WANT\_FOOD} \leftarrow True$
R_2:			
$\mathcal{P}_2$	$P_{ON\_SLOPE} == False$	$P_{MOTIVATION_2} <= 0.5$	
$\mathcal{B}_2$	B_{RANDOM_WALK}	$B_{GO\_TO\_SOURCE}$	
$\mathcal{A}_2$	A_B	$p = 0.025$	$B_{GO\_TO\_SOURCE}$
R_3:			
$\mathcal{P}_3$	$P_{ON\_SLOPE} == True$	$P_{MOTIVATION_1} <= 0.5$	
$\mathcal{B}_3$	B_{RANDOM_WALK}	$B_{GO\_TO\_NEST}$	
$\mathcal{A}_3$	A_B<br>A_{IS}	$p = 0.075$<br>$p = 0.075$	B_{RANDOM_WALK}<br>$IS_{MOTIVATION_1} +$
R_4:			
$\mathcal{P}_4$	$P_{MOTIVATION_1} <= 0.5$		
$\mathcal{B}_4$	$B_{GO\_TO\_NEST}$	$B_{GO\_TO\_SOURCE}$	
$\mathcal{A}_4$	A_{IS}<br>A_{IS}<br>A_{IS}	$p = 0.05$<br>$p = 0.001$<br>$p = 0.01$	$IS_{MOTIVATION_2} -$<br>$IS_{DROP\_FOOD} \leftarrow True$<br>$IS_{MOTIVATION_1} -$
R_5:			
$\mathcal{P}_5$	$P_{HAS\_FOOD} == True$		
$\mathcal{B}_5$	B_{RANDOM_WALK}	$B_{GO\_TO\_SOURCE}$	
$\mathcal{A}_5$	A_B<br>A_{IS}<br>A_{IS}<br>A_B	$p = 1.0$<br>$p = 0.01$<br>$p = 0.075$<br>$p = 0.05$	$B_{GO\_TO\_SOURCE}$<br>$IS_{MOTIVATION_2} -$<br>$IS_{DROP\_FOOD} \leftarrow True$<br>$B_{GO\_TO\_NEST}$
R_6:			
$\mathcal{P}_6$	$P_{ON\_SOURCE} == True$		
$\mathcal{B}_6$	$B_{GO\_TO\_NEST}$		
$\mathcal{A}_6$	A_{IS}	$p = 0.025$	$IS_{MOTIVATION_1} +$

Evolved controller 1

R_1:			
$\mathcal{P}_1$	$P_{ON\_SLOPE} == True$	$P_{ON\_GRASS} == False$	$P_{ON\_NEST} == False$
$\mathcal{B}_1$	B_{RANDOM_WALK}		
$\mathcal{A}_1$	A_{IS}	$p = 0.001$	$IS_{WANT\_FOOD} \leftarrow False$
R_2:			
$\mathcal{P}_2$	$P_{MOTIVATION_1} <= 0.5$	$P_{ON\_SOURCE} == False$	
$\mathcal{B}_2$	B_{RANDOM_WALK}	$B_{GO\_TO\_NEST}$	
$\mathcal{A}_2$	A_B<br>A_{IS}<br>A_B<br>A_{IS}	$p = 0.1$<br>$p = 0.1$<br>$p = 0.01$<br>$p = 0.05$	$B_{GO\_TO\_NEST}$<br>$IS_{MOTIVATION_2}$ +<br>$B_{GO\_TO\_SOURCE}$<br>$IS_{MOTIVATION_1}$ −
R_3:			
$\mathcal{P}_3$	$P_{HAS\_FOOD} == True$	$P_{MOTIVATION_1} <= 0.5$	
$\mathcal{B}_3$	$B_{GO\_TO\_SOURCE}$		
$\mathcal{A}_3$	A_{IS}<br>A_B	$p = 0.1$<br>$p = 0.075$	$IS_{DROP\_FOOD} \leftarrow True$<br>$B_{GO\_TO\_NEST}$

Evolved controller 10
