# Supplementary material for: Evolution of Self-Organized Task Specialization in Robot Swarms
Source: PLoS Comput Biol. 2015 Aug 6;11(8):e1004273. doi: 10.1371/journal.pcbi.1004273 (PMC4527708; doi:10.1371/journal.pcbi.1004273)
Supplement: S3 Table — Controllers are sorted from high to low group performance. (PDF) [file pcbi.1004273.s004.pdf]

Table S3. Performance of the 22 evolved controllers and degree of task partitioning observed in the 4 robot teams and in the 20 robot ones used during post-validation.

	**4 robots**		**20 robots**	
**Evolution Run**	**Degree of task partitioning** $(\bar{x} \pm SD)$	**Fitness** $(\bar{x} \pm SD)$	**Degree of task partitioning** $(\bar{x} \pm SD)$	**Fitness** $(\bar{x} \pm SD)$
20	1 ± 0	134.53 ± 13.8	0.97 ± 0.02	596.07 ± 35.24
9	0.98 ± 0.02	125.3 ± 11.01	1 ± 0	577.9 ± 47.55
5	0.94 ± 0.04	122.57 ± 15.82	1 ± 0	542.63 ± 36.01
19	1 ± 0	119.93 ± 16.26	0.95 ± 0.02	536.67 ± 58.71
11	0.91 ± 0.05	106.67 ± 11.36	1 ± 0	451.73 ± 43.54
2	0.9 ± 0.08	103.07 ± 23.97	1 ± 0	457.03 ± 71.57
3	0.76 ± 0.32	102.87 ± 45.57	1 ± 0	448.43 ± 78.27
14	0.88 ± 0.1	97.2 ± 19.44	1 ± 0	406.5 ± 48.29
15	0.94 ± 0.03	39.23 ± 3.88	0.78 ± 0.03	194.3 ± 10.64
12	0 ± 0	38.73 ± 1.05	0 ± 0	172.9 ± 4.44
22	0 ± 0	35.9 ± 2.28	0 ± 0	150 ± 8.54
4	0 ± 0	35.87 ± 1.53	0 ± 0	134.07 ± 7.83
8	0.81 ± 0.29	35.6 ± 16.08	1 ± 0	157.7 ± 30.07
16	0.91 ± 0.05	33.43 ± 3.01	0.75 ± 0.03	169.97 ± 7.3
18	0 ± 0	32.93 ± 4.65	0 ± 0	129.27 ± 17.5
21	0 ± 0	29.7 ± 5.91	0 ± 0	116.07 ± 12.09
7	0 ± 0	27.57 ± 6.56	0 ± 0	79.93 ± 15.87
13	0 ± 0.01	26.83 ± 2.61	0.91 ± 0.03	116.13 ± 8.33
6	0.06 ± 0.08	22.87 ± 4.88	0.87 ± 0.04	89.3 ± 15.91
17	0.97 ± 0.18	22.43 ± 17.15	0.58 ± 0.14	58.93 ± 21.75
1	0.62 ± 0.31	20.03 ± 11.89	0.99 ± 0.01	71.37 ± 28.2
10	0.08 ± 0.11	18.4 ± 8.05	0.96 ± 0.02	80.9 ± 19.46
